# RIP140 deficiency enhances cardiac fuel metabolism and protects mice from heart failure

**DOI:** 10.1172/JCI162309

**Published:** 2023-05-01

**Authors:** Tsunehisa Yamamoto, Santosh K. Maurya, Elizabeth Pruzinsky, Kirill Batmanov, Yang Xiao, Sarah M. Sulon, Tomoya Sakamoto, Yang Wang, Ling Lai, Kendra S. McDaid, Swapnil V. Shewale, Teresa C. Leone, Timothy R. Koves, Deborah M. Muoio, Pieterjan Dierickx, Mitchell A. Lazar, E. Douglas Lewandowski, Daniel P. Kelly

**Affiliations:** 1 Cardiovascular Institute, Department of Medicine, Perelman School of Medicine at the University of Pennsylvania, Philadelphia, Pennsylvania, USA.; 2 Davis Heart and Lung Research Institute and Department of Internal Medicine, The Ohio State University College of Medicine, Columbus, Ohio, USA.; 3 Institute for Diabetes, Obesity and Metabolism, and Division of Endocrinology, Diabetes, and Metabolism, Perelman School of Medicine at the University of Pennsylvania, Philadelphia, Pennsylvania, USA.; 4 Departments of Medicine and Pharmacology and Cancer Biology, and Duke Molecular Physiology Institute, Duke University, Durham, North Carolina, USA.

**Keywords:** Cardiology, Heart failure, Mitochondria, Transcription

## Abstract

During the development of heart failure (HF), the capacity for cardiomyocyte (CM) fatty acid oxidation (FAO) and ATP production is progressively diminished, contributing to pathologic cardiac hypertrophy and contractile dysfunction. Receptor-interacting protein 140 (RIP140, encoded by *Nrip1*) has been shown to function as a transcriptional corepressor of oxidative metabolism. We found that mice with striated muscle deficiency of RIP140 (str*Nrip1^–/–^*) exhibited increased expression of a broad array of genes involved in mitochondrial energy metabolism and contractile function in heart and skeletal muscle. str*Nrip1^–/–^* mice were resistant to the development of pressure overload–induced cardiac hypertrophy, and CM-specific RIP140-deficient (cs*Nrip1^–/–^*) mice were protected against the development of HF caused by pressure overload combined with myocardial infarction. Genomic enhancers activated by RIP140 deficiency in CMs were enriched in binding motifs for transcriptional regulators of mitochondrial function (estrogen-related receptor) and cardiac contractile proteins (myocyte enhancer factor 2). Consistent with a role in the control of cardiac fatty acid oxidation, loss of RIP140 in heart resulted in augmented triacylglyceride turnover and fatty acid utilization. We conclude that RIP140 functions as a suppressor of a transcriptional regulatory network that controls cardiac fuel metabolism and contractile function, representing a potential therapeutic target for the treatment of HF.

## Graphical abstract

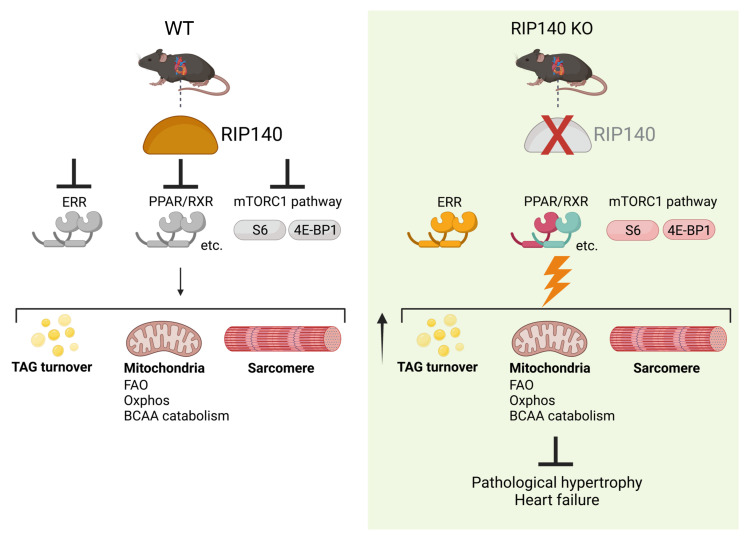

## Introduction

The remarkable energy demands of the heart are largely met by the oxidation of fatty acids and glucose in high-capacity mitochondrial networks. Fatty acid oxidation (FAO) is the chief source of adenosine triphosphate (ATP) in normal adult heart. The capacity for cardiomyocyte (CM) mitochondria to burn lipid fuels is dynamically regulated during development and by diverse physiological and pathophysiological conditions. For example, cardiac mitochondrial FAO capacity increases following birth and with exercise training (1, 2). Conversely, mitochondrial FAO is diminished in the hypertrophied and failing heart (3).

Mitochondrial respiratory capacity and fuel oxidation preferences are defined in a tissue-specific manner during postnatal developmental maturation. In the heart, this process begins shortly after birth with a dramatic mitochondrial biogenesis event driven by the transcriptional coregulator PPAR coactivator 1 (PGC-1), acting upon downstream transcription factor effectors including the nuclear receptors estrogen-related receptor (ERR) and PPAR (3). Following the mitochondrial biogenic response, cardiac mitochondria undergo maturational remodeling during the postnatal period, resulting in an enhanced capacity for FAO together with a shift from fetal to adult contractile proteins. Recently, we have shown that ERRα and -γ are critical for metabolic and contractile postnatal maturation in heart (4, 5). During the development of pathologic cardiac hypertrophy and in the failing heart, the PGC-1/ERR/PPAR circuitry is downregulated, reducing the capacity for mitochondrial FAO and respiration, resulting in a reliance on glycolytic and glucose oxidation pathways (3, 6). This mitochondrial reprogramming sets the stage for an “energy-starved” failing heart.

In vivo approaches to augment the transcriptional control of cardiac mitochondrial oxidative capacity in animal models of heart failure (HF) have largely involved overexpression strategies. For example, overexpression of PGC-1α in mouse heart triggers a mitochondrial biogenesis response that ultimately becomes uncontrolled, leading to cardiomyopathy (7, 8). From a translational perspective, a preferred strategy might involve boosting the activity of central components of the PGC-1–regulated cascade without artificial overexpression strategies. One approach could be to inhibit endogenous suppressors or natural “brakes” on this transcriptional regulatory circuitry. To this end, we have focused on the transcriptional corepressor receptor interacting protein 1 (RIP140, encoded by *Nrip1*), which has been shown to serve as a corepressor of ERR and PPAR signaling in noncardiac cells (9). Mice with generalized *Nrip1* deficiency exhibit a lean phenotype with enhanced mitochondrial respiratory function in adipose tissue and skeletal muscle (10, 11). However, the role of RIP140 as a tissue-autonomous regulator of mitochondrial fuel and energy metabolism in heart has not, to our knowledge, been assessed. Here, we demonstrate that cardiac RIP140 deficiency protected against ventricular pressure overload–induced cardiac hypertrophy and HF. Nuclear magnetic resonance–based (NMR-based) interrogation of RIP140-deficient hearts demonstrated enhanced rates of myocardial triacylglyceride (TAG) turnover and utilization. Thus, inhibition of RIP140 in heart maintained a high capacity for oxidation of fatty acids and preserved contractile function under pathophysiologic conditions that cause HF in WT hearts.

## Results

### Increased expression of genes involved in mitochondrial energy metabolism and contractile function in RIP140-deficient heart and skeletal muscle.

To explore the role of RIP140 in striated muscle, *Nrip1*-floxed (*Nrip1^fl/fl^*) mice were crossed with mice expressing Cre recombinase under the direction of the muscle creatine kinase (MCK) promoter (12) to generate cardiac and skeletal muscle RIP140-deficient mice (str*Nrip1^–/–^* mice) (Supplemental Figure 1, A–C; supplemental material available online with this article; https://doi.org/10.1172/JCI162309DS1). str*Nrip1^–/–^* mice were born at the expected Mendelian ratios and exhibited normal growth and survival during the postnatal period (data not shown).

**Figure 1:**
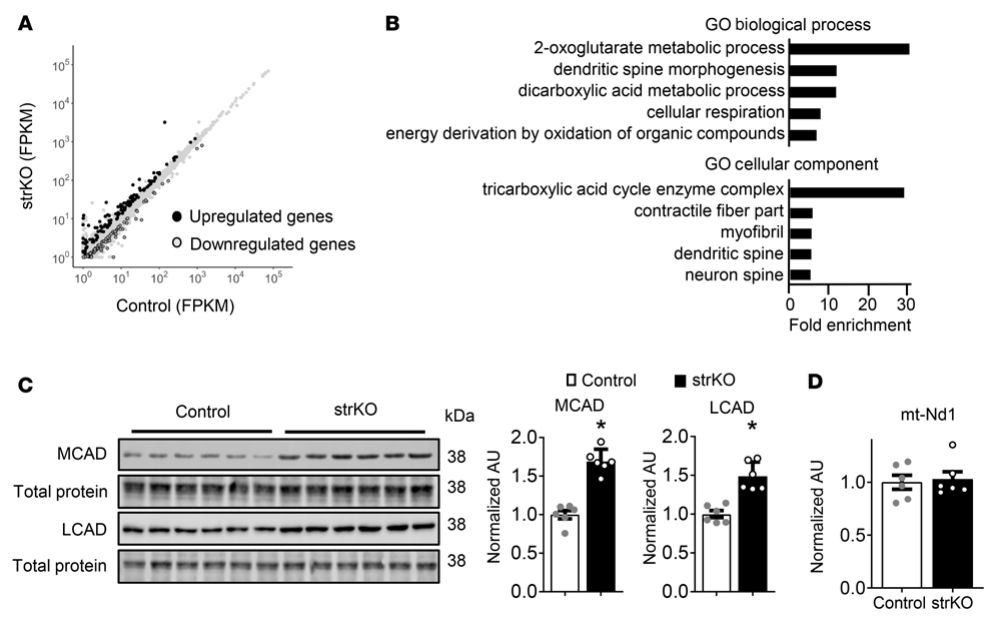
Genes involved in mitochondrial fuel oxidation, OXPHOS, and the adult sarcomere program are upregulated in str*Nrip1^–/–^* hearts. Global RNA-Seq was performed on biventricular heart samples from 8-week-old male control and str*Nrip1^–/–^* (strKO) mice (*n* = 3 per group). (**A**) Scatter plot represents upregulated (black circles) and downregulated (gray circles) DEGs in strKO hearts compared with control hearts. Fold change (FC) >1.2; FDR <0.05. (**B**) KEGG pathways enriched in strKO upregulated genes. (**C**) Left: Representative immunoblots of MCAD and LCAD in 8-week-old male heart. Right: Bar graphs show quantification of the immunoblots in normalized AU (*n* = 6 per group). (**D**) Relative levels of the mtDNA marker (mt-Nd1) normalized to the nuclear DNA amount (*n* = 6 per group). Values are the mean ± SEM. *P < 0.05 versus control, by 2-tailed, unpaired Student’s *t* test.

To determine the impact of *Nrip1* gene deletion on cardiac gene expression, we conducted genome-wide RNA-Seq on cardiac ventricles of 8-week-old male str*Nrip1^–/–^* and littermate control mice. RNA-Seq analysis identified 190 upregulated genes and 101 downregulated genes in str*Nrip1^–/–^* hearts compared with control hearts (Figure 1A). Kyoto Encyclopedia of Genes and Genomes (KEGG) pathway analysis of the upregulated genes revealed enrichment in pathways and processes involved in mitochondrial oxidative energy metabolism and respiration (Figure 1B and Supplemental Table 1). Further analysis of differentially expressed genes (DEGs) in the RIP140-deficient heart demonstrated upregulated expression of genes involved in FAO (*Cpt1b, Acox1*, *Acadm*, *Acadl*, *Acacb*, *Acss1*, *Slc27a1*, *Slc25a20*, *Hadha*, *Ech1*, and *Echdc2*); the TCA cycle (*Idh3g*, *Idh2*, *Ogdh*, *Suclg2*, *Odghl*, and *Sdhc*); oxidative phosphorylation (OXPHOS) (*Cox4i1*, *Cox6a2*, *Cox7a1*, and *Ndufv1*); and branched-chain amino acid (BCAA) catabolism (*Bcat2,* *Bckdha, Bckdk, Hadha, Auh, Aldh2, Ivd,* and *Mccc1*). In addition, the expression of a number of genes involved in the adult program of contractile function (*Tnni3*, *Myh6*, *Myl3*, *Atp1a1*, *Cacng6*, and *Mybpc3*) was also increased in str*Nrip1^–/–^* hearts. We confirmed the regulation of a subset of DEGs by quantitative reverse transcription PCR (qRT-PCR) (Supplemental Figure 1D) and immunoblot analyses (Figure 1C). Despite the increased expression of genes involved in mitochondrial fuel and energy metabolism, mitochondrial DNA (mtDNA) was not significantly increased in the str*Nrip1^–/–^* hearts (Figure 1D). In addition, the expression of genes encoding PPARα and -δ, ERRα and -δ, and the coactivator PGC-1α, known regulators of genes involved in mitochondrial biogenesis and metabolism, were not changed in str*Nrip1^–/–^* hearts (data not shown).

Despite normal body growth, the hearts of adult (8-week-old) str*Nrip1^–/–^* mice were slightly larger than those of controls, as evidenced by an increased biventricular weight to tibia length (BV/TL) ratio, left ventricular (LV) mass index (LVMI), LV wall thickness, and CM size (Supplemental Figure 2, A–C, and Supplemental Table 2). However, LV systolic function (LV fractional shortening) was normal in str*Nrip1^–/–^* mice, and cardiac stress gene markers (*Nppa* and *Nppb*) were not elevated in str*Nrip1^–/–^* mice (Supplemental Figure 2, B and D).

The effect of RIP140 deficiency on skeletal muscle gene expression was also determined. RNA-Seq of str*Nrip1^–/–^* gastrocnemius muscle identified 1,045 upregulated genes and 923 downregulated genes in str*Nrip1^–/–^* compared with control muscle (Supplemental Figure 3A). Similar to that of the str*Nrip1^–/–^* heart, KEGG pathway analysis of upregulated genes revealed strong enrichment in pathways involved in mitochondrial fuel oxidation and OXPHOS (Supplemental Figure 3B and Supplemental Table 3). We confirmed the regulation of a subset of DEGs by qRT-PCR (Supplemental Figure 3C). In addition, protein levels of representative enzymes involved in fatty acid utilization including FAO (MCAD, LCAD) and TAG dynamics (DGAT2, ATGL) were increased in RIP140-deficient muscle, as determined by immunoblot analyses (Supplemental Figure 3D). RNA-Seq of str*Nrip1^–/–^* soleus muscle showed a similar general pattern compared with that of gastrocnemius muscle, but the degree of differential expression of genes (and pathway enrichments) was less pronounced (Supplemental Table 4). Interestingly, however, the expression of genes involved in glycolysis was downregulated in soleus muscle (Supplemental Table 4).

### RIP140 deficiency protects against pathological cardiac hypertrophy remodeling and dysfunction.

**Figure 2:**
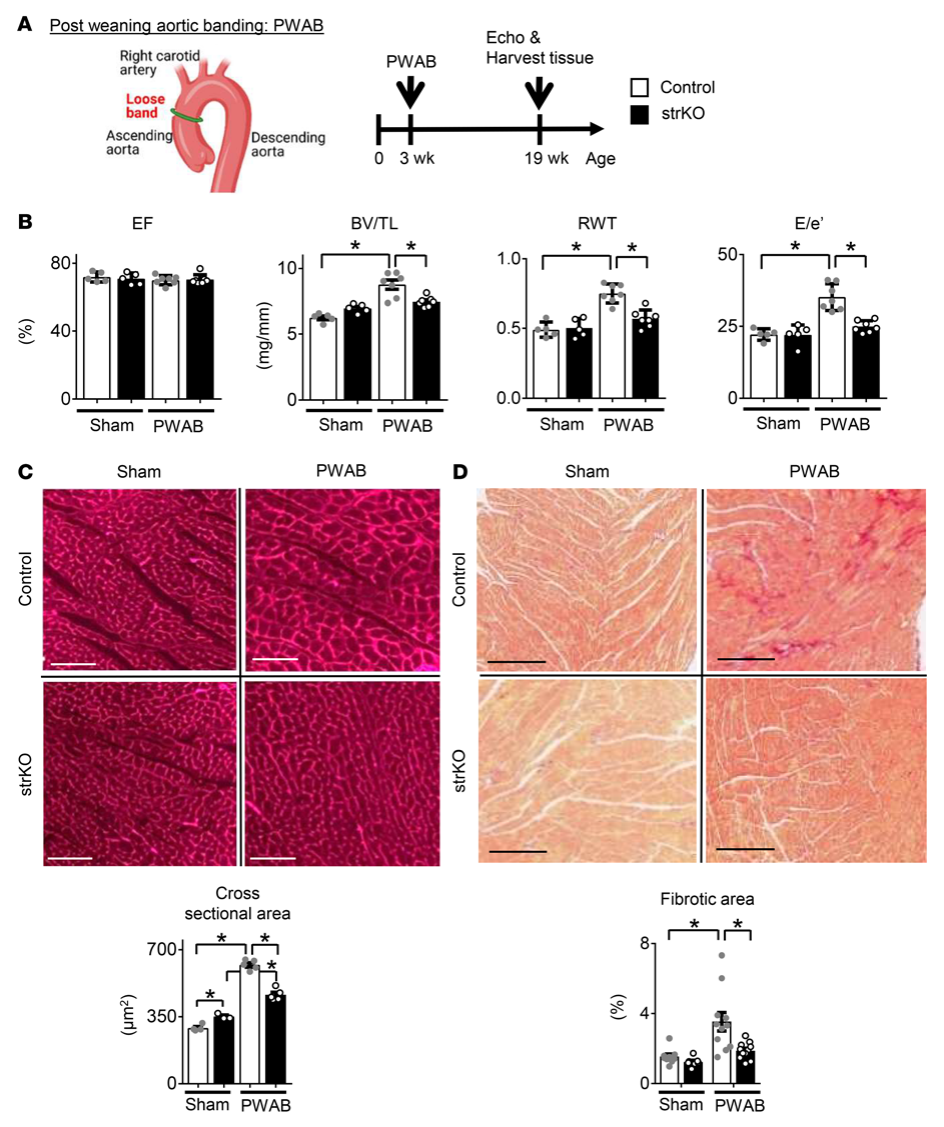
str*Nrip1^–/–^* mice are protected against cardiac hypertrophy and diastolic dysfunction in response to chronic pressure overload. (**A**) Schematic depicts PWAB surgery and the experimental timeline (*n* = 6–9 mice per group). (**B**) LVEF, the BV/TL ratio, LV RWT, and the E/e′ wave ratio. (**C**) Representative images of heart sections stained with wheat germ agglutinin (WGA). Scale bars: 100 μm. Quantification of CM size is shown in the graph. (**D**) Representative images of heart sections stained with Picrosirius red. Scale bars: 300 μm. Quantification of fibrosis area is shown in the graph. Values are the mean ± SEM. *P < 0.05, by 2-way ANOVA with Tukey’s multiple-comparison test. The PWAB illustration was created with BioRender.com.

Mitochondrial oxidative capacity and FAO are downregulated during the development of pathological cardiac hypertrophy (3) coincident with increased glucose utilization. Evidence has emerged that this fuel shift is necessary for cardiac hypertrophic growth by diverting carbons from glycolysis and pyruvate to biosynthetic pathways (13, 14). Given the observed upregulated expression of genes involved in mitochondrial FAO in the RIP140-deficient hearts, we sought to assess the cardiac hypertrophic growth response in str*Nrip1^–/–^* mice. For these studies, chronic pressure overload was imposed using post-weaning aortic banding (PWAB) (15). The PWAB model involves placement of a “loose” band around the ascending aorta in 3-week-old male mice, which produces gradual and progressive LV hypertrophy and diastolic dysfunction over the ensuing 16 weeks (Figure 2A) with maintenance of systolic LV function, a phenotype that shares similarities with HF with preserved ejection fraction (HFpEF). The control mice displayed a robust hypertrophic response, as indicated by increases in the LVMI, BV/TL, and relative wall thickness (RWT) with pEF (Figure 2B and Supplemental Table 4). Remarkably, the str*Nrip1^–/–^* mice did not develop LV hypertrophy despite being exposed to a pressure overload similar to that of control mice, based on peak velocity measurements across the aortic band (Supplemental Table 5). Moreover, whereas LV diastolic dysfunction developed in the control PWAB group, as reflected by an increased ratio of early LV filling (E wave)/tissue Doppler imaging of mitral annular motion (e′ wave), E/e′ was not increased in the str*Nrip1^–/–^* mice (Figure 2B and Supplemental Table 5). Histologic analysis of CM size demonstrated that the cell area was increased to a greater extent in str*Nrip1^–/–^* hearts in response to PWAB (Figure 2C). In addition, the str*Nrip1^–/–^* mice were protected against PWAB-induced myocardial fibrosis (Figure 2D). We observed a similar resistance to hypertrophic cardiac remodeling in female str*Nrip1^–/–^* mice (Supplemental Table 6). These results indicate that str*Nrip1^–/–^* mice were protected against LV hypertrophy and diastolic dysfunction in response to chronic progressive LV pressure overload.

To further investigate the cardiac phenotype of RIP140 deficiency, we generated mice with CM-selective RIP140 deficiency to avoid the confounding effects of genetic manipulation in skeletal muscle. We crossed the *Nrip1^fl/fl^* mouse line with mice expressing Cre recombinase under the control of the cardiac-specific NKX2.5 promoter (Nkx2.5-Cre) to generate CM-specific RIP140-deficient (cs*Nrip1^–/–^*) mice (Supplemental Figure 4A). cs*Nrip1^–/–^* mice had a phenotype similar to that of str*Nrip1^–/–^* mice with normal survival, normal body growth, and mild cardiac hypertrophy with normal LV systolic function without induction of pathological hypertrophic gene markers (Supplemental Figure 4, B–D, and Supplemental Table 7). We also confirmed that the expression of genes involved in FAO (*Cpt1b*, *Acadm*, *Slc27a1*, *Hadha*, and *Ech1*), the TCA cycle and OXPHOS (*Idh2*, *Ndufv1*, and *Cox6a2*), and BCAA catabolism (*Bckdha* and *Ivd*) was increased (Supplemental Figure 4E), similar to what we observed in str*Nrip1^–/–^* hearts. The protein levels of 2 representative FAO genes, MCAD and LCAD, were increased in cs*Nrip1^–/–^* hearts (Supplemental Figure 4F). Notably, protein levels of PPARα, ERRα, ERRγ, and PGC-1α were similar in cs*Nrip1^–/–^* and control hearts (data not shown).

**Figure 3:**
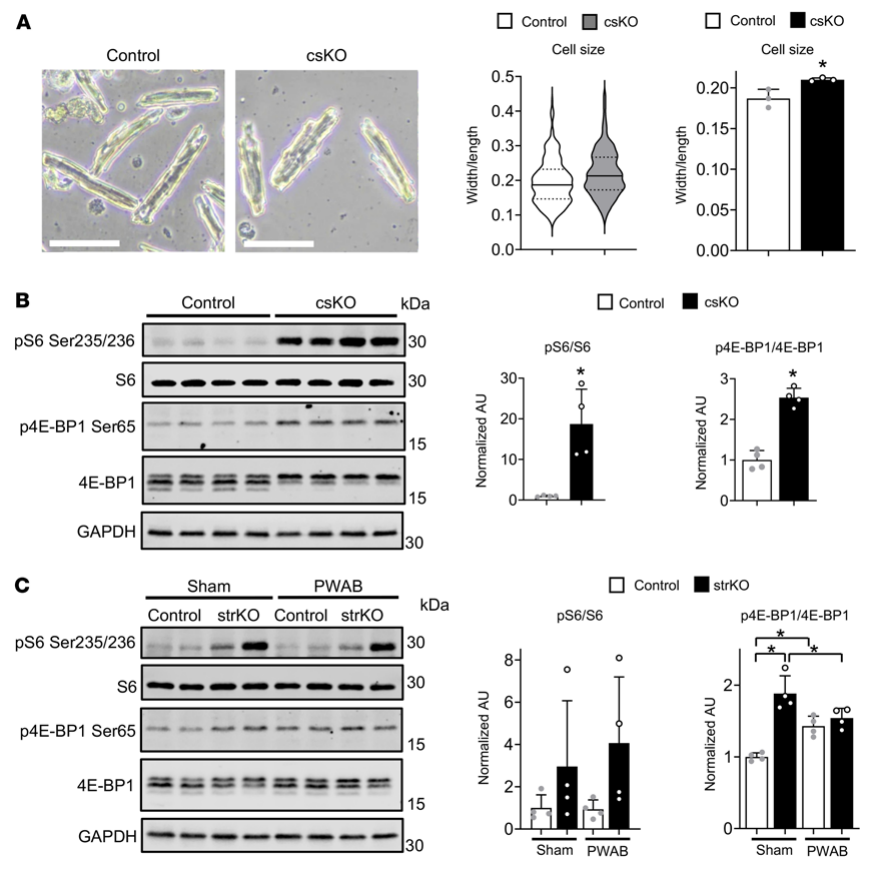
RIP140-deficient CMs exhibit hypertrophy and activation of the mTORC1 signaling pathway. (**A**) Representative images of isolated CMs from 8- to 10-week-old male cs*Nrip1^–/–^* (csKO) and WT (Control) mouse hearts. Scale bars: 50 μm. Graphs show quantification of CM size (*n* = 3 per group, 100 cells per sample). (**B**) Representative immunoblots of mTORC1 signaling targets S6 ribosomal protein and 4E-BP1 from 10- to 13-week-old male control and csKO hearts. Graphs show quantification of immunoblots in normalized AU (*n* = 4 per group). (**C**) Representative immunoblots of mTORC1 signaling targets S6 ribosomal protein and 4E-BP1 from male str*Nrip1^–/–^* (strKO) and WT (Control) mouse hearts following PWAB or sham surgery. Graphs show quantification of immunoblots in normalized AU (*n* = 4 per group). Values are the mean ± SEM. *P < 0.05 versus control, by 2-tailed, unpaired Student’s *t* test (**A** and **B**) or 2-way ANOVA with Tukey’s multiple-comparison test (**C**). p, phosphorylated.

The modest cardiac hypertrophy with normal function observed with echocardiographic analysis of the cs*Nrip1^–/–^* hearts suggested an adaptive or physiological form of cardiac hypertrophy such as occurs with exercise training. CMs isolated from cs*Nrip1^–/–^* mice were modestly but significantly larger in size (width/length ratio), further confirming a hypertrophic phenotype at baseline (Figure 3A). To further explore the basis for this phenotype, we probed candidate cellular signaling pathways known to be involved in CM hypertrophic growth in the hearts of cs*Nrip1^–/–^* mice. Phosphorylation of S6 and 4E-BP1, downstream markers of mTORC1 activation, was significantly increased in cs*Nrip1^–/–^* hearts (Figure 3B). In contrast, phosphorylation levels of AKT, AMPK, ERK, and STAT3 were unchanged or undetected (data not shown), suggesting that the activation of mTORC1 activity was independent of these pathways. Notably, mTORC1 signaling has been implicated in the mechanism of physiological hypertrophy and its protection against pathological insult (16, 17). We also probed the activity of the mTORC1 pathway in the heart samples harvested from the PWAB study conducted in the str*Nrip1^–/–^* mice (Figure 2). Interestingly, we found that the increased phosphorylation of 4E-BP1 was unchanged with chronic pressure overload in the str*Nrip1^–/–^* hearts, whereas phosphorylation was increased in the WT controls, while S6 phosphorylation was unchanged (Figure 3C). These collective results demonstrate that mTORC1 signaling was activated in RIP140-deficient hearts, consistent with a physiological hypertrophy phenotype, and identify a potential mechanism involved in the defense of the str*Nrip1^–/–^* hearts against PWAB-induced pathologic hypertrophic growth.

**Figure 4:**
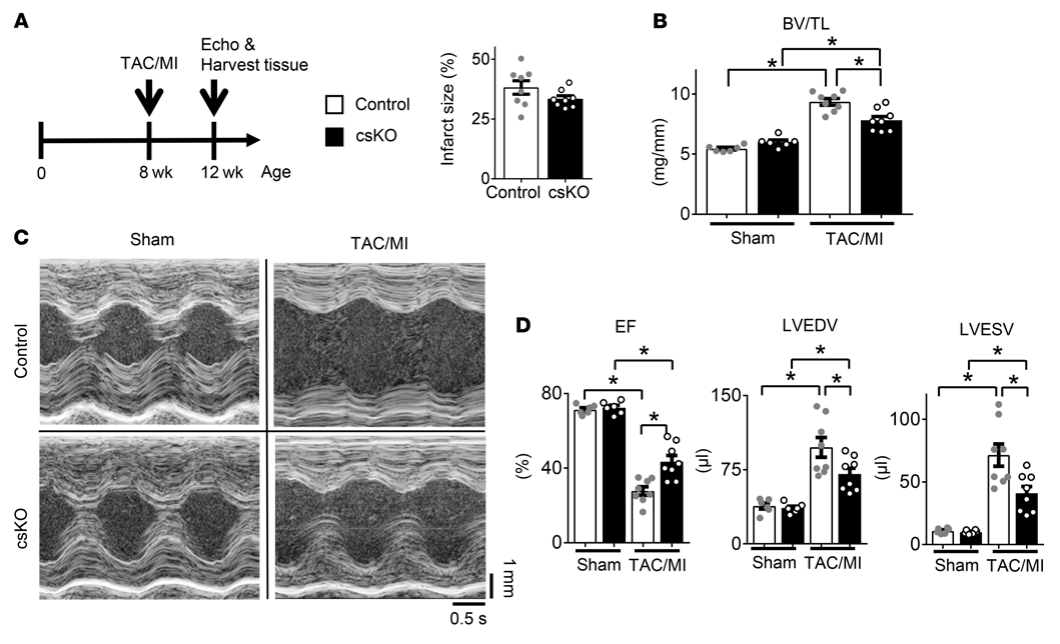
Cardiac-specific RIP140 deficiency (csKO) attenuates TAC/MI-induced LV remodeling and systolic dysfunction. (**A**) Schematic shows the experimental timeline for studies in which 8-week-old male mice were subjected to TAC combined with TAC/MI to induce HF (*n* = 6–8 per group). Graphs show LV infarct size in cs*Nrip1^–/–^* and littermate WT mice as determined via echocardiographic assessment of wall motion defects (*n* = 8 mice per group). (**B**) BV/TL ratio. (**C**) Representative LV M-mode echocardiographic tracings. (**D**) LVEF, LVEDV, and LVESV. Values are the mean ± SEM. *P < 0.05, by 2-way ANOVA with Tukey’s multiple-comparison test.

The observed protection against pathologic cardiac hypertrophy and diastolic dysfunction exhibited by the str*Nrip1^–/–^* mice in response to PWAB led us to explore the impact of cardiac RIP140 deficiency in HF with reduced EF. To this end, the cs*Nrip1^–/–^* mice were subjected to transverse aortic constriction combined with small apical myocardial infarction (TAC/MI) surgery, which results in progressive LV dilatation and systolic dysfunction over the ensuing 4 weeks (18, 19) (Figure 4A). The MI size, as determined by echocardiographic assessment of wall motion abnormalities (see Methods), was not different between the groups (Figure 4A). Similar to the response of the str*Nrip1^–/–^* mice subjected to PWAB, the LV hypertrophic response was significantly diminished in the cs*Nrip1^–/–^* male mice after TAC/MI (Figure 4B and Supplemental Table 8). In addition, we noted substantially less LV dilatation and dysfunction in the cs*Nrip1^–/–^* mice. Specifically, LV end-diastolic volume (LVEDV) and LV end-systolic volume (LVESV) were increased to a lesser extent in cs*Nrip1^–/–^* mice following TAC/MI compared with controls (Figure 4, C and D, and Supplemental Table 8), and the reduction in LVEF was significantly less in the RIP140-deficient mice (Figure 4, C and D, and Supplemental Table 8). These results demonstrate that cs*Nrip1^–/–^* mice were partially protected against the development of HF caused by chronic LV pressure overload and ischemic insult.

### Genomic interrogation identifies cardiac RIP140-suppressed pathways relevant to the cardioprotective phenotype of csNrip1^–/–^ mice.

**Figure 5:**
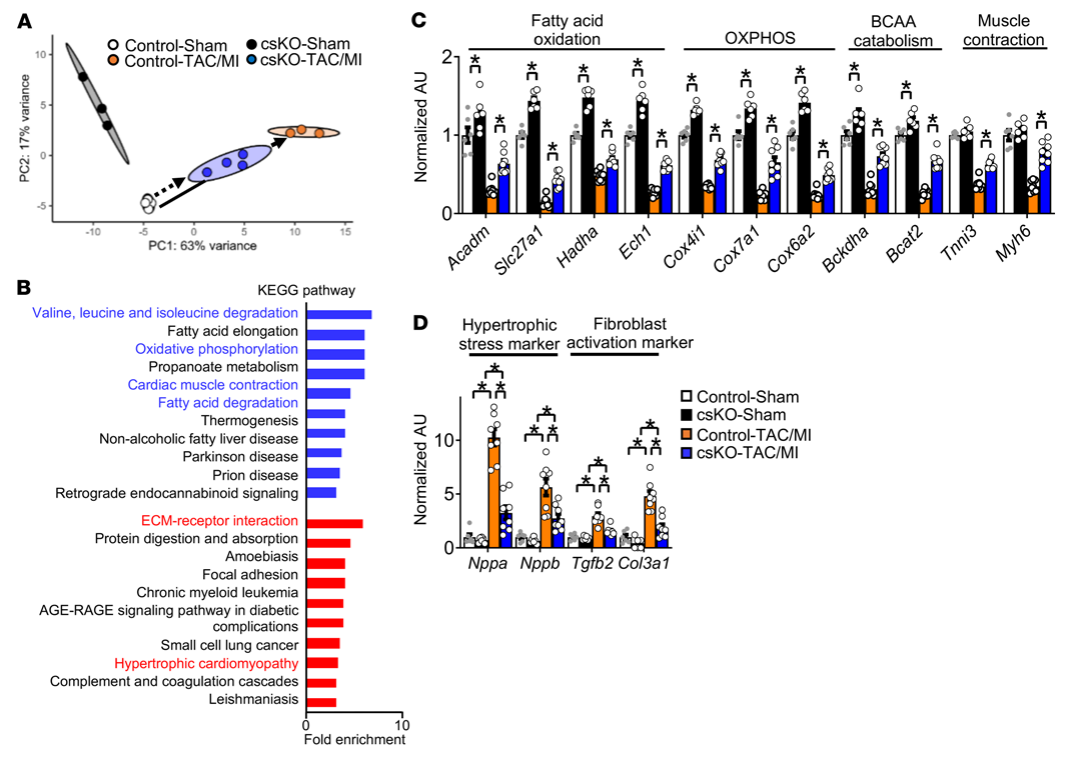
TAC/MI-mediated differential expression of genes involved in mitochondrial energy transduction and partial protection of adult cardiac sarcomeres by RIP140 deficiency after TAC/MI. (**A**) PCA plots for RNA-Seq data obtained from biventricles of sham- and TAC/MI-operated cs*Nrip1^–/–^* and WT littermate mice. Solid arrow indicates gene expression changes, comparing the control sham-operated and control TAC/MI groups. Dashed arrow indicates a comparison of csKO TAC/MI with the control sham group. (**B**) KEGG pathways enriched in genes protected from downregulation (blue) and upregulation (red) by cardiac RIP140 deficiency during TAC/MI. “Protected” genes are defined as significantly less upregulated or downregulated following TAC/MI in csKO versus control hearts, with both compared with that of control sham-operated mouse hearts as described in the Results (difference between solid and dotted lines in panel **A**) using the cutoff: FC >1.2, FDR <0.05. (**C**) qRT-PCR validation of genes involved in mitochondrial fuel and energy metabolic pathways including FAO genes (*Acadm*, *Slc27a1*, *Hadha*, and *Ech1)*; OXPHOS genes (*Cox4i1*, *Cox7a1*, and *Cox6a2)*; BCAA catabolism genes (*Bckdha* and *Bcat2),* as well as adult contractile protein genes (*Tnni3* and *Myh6*) (*n* = 6–8 per group). (**D**) qRT-PCR validation of cardiac hypertrophic stress marker genes (*Nppa* and *Nppb*) and fibroblast activation marker genes (*Tgfb2* and *Col3a1*) (*n* = 6–9 per group). Values are the mean ± SEM. *P < 0.05, by 2-way ANOVA with Tukey’s multiple-comparison test.

As an initial step to define gene-regulatory events involved in the cardioprotective effect of cardiac *Nrip1* deletion, we conducted RNA-Seq on cs*Nrip1^–/–^* and cs*Nrip1*^+/+^ littermate control hearts 4 weeks after sham or TAC/MI surgery. Principal component analysis (PCA) of the gene expression patterns delineated the 4 distinct groups (Figure 5A). As expected, TAC/MI surgery resulted in significant gene expression reprogramming compared with sham-operated hearts in the cs*Nrip1^+/+^* groups, including, as expected, many DEGs involved in mitochondrial energy metabolism and contractile function (Figure 5A; solid arrow). Notably, this effect was substantially attenuated for a large number of genes in the cs*Nrip1^–/–^* group (Figure 5A; dashed arrow). We quantified this attenuation statistically by identifying genes that had (a) differential expression between the sham and TAC/MI groups for the control genotype; (b) differential expression between the control and cs*Nrip1^–/–^* groups with TAC/MI; and (c) differential expression between the control and TAC/MI groups and were diminished with RIP140 deficiency. This approach defined a set of “protected” genes, in which the effect of TAC/MI surgery on DEGs was significantly (FDR <0.05) reduced. We identified 2,206 genes protected by RIP140 deficiency using this approach (1,213 genes significantly less upregulated, 993 genes less downregulated). Genes that were less downregulated were enriched in FAO, BCAA catabolism, OXPHOS, and cardiac muscle contraction pathways (Figure 5, B and D, and Supplemental Table 9), whereas genes protected from upregulation were enriched in fibrosis and hypertrophy pathways, among others (Figure 5, B and C, and Supplemental Table 9). A more extensive list of the pathway analysis of the “protected genes” is shown in Supplemental Figure 5 and Supplemental Table 10.

Genes that were less downregulated by TAC/MI in cs*Nrip1^–/–^* hearts were of particular interest, given that the majority were involved in mitochondrial fuel metabolism and respiratory function. Specifically, genes encoding enzymes in mitochondrial fatty acid import and nearly every step of the β-oxidation cycle were protected by RIP140 deficiency (see pathway schematic in Supplemental Figure 6). Similarly, genes encoding components in each of the respiratory complexes (Supplemental Figure 7) and enzymes involved in BCAA degradation (Supplemental Figure 8) were less differentially expressed in the cs*Nrip1^–/–^* hearts subjected to TAC/MI. A subset of these protected gene expression changes in FAO, BCAA catabolism, OXPHOS, and muscle contraction were validated at the transcript (Figure 5D) and protein (Supplemental Figure 9, A and B) levels. Notably, in contrast to RIP140-deficient hearts at baseline, post-TAC/MI cs*Nrip1^–/–^* hearts had higher levels of mtDNA than did controls (Supplemental Figure 9C).

**Figure 6:**
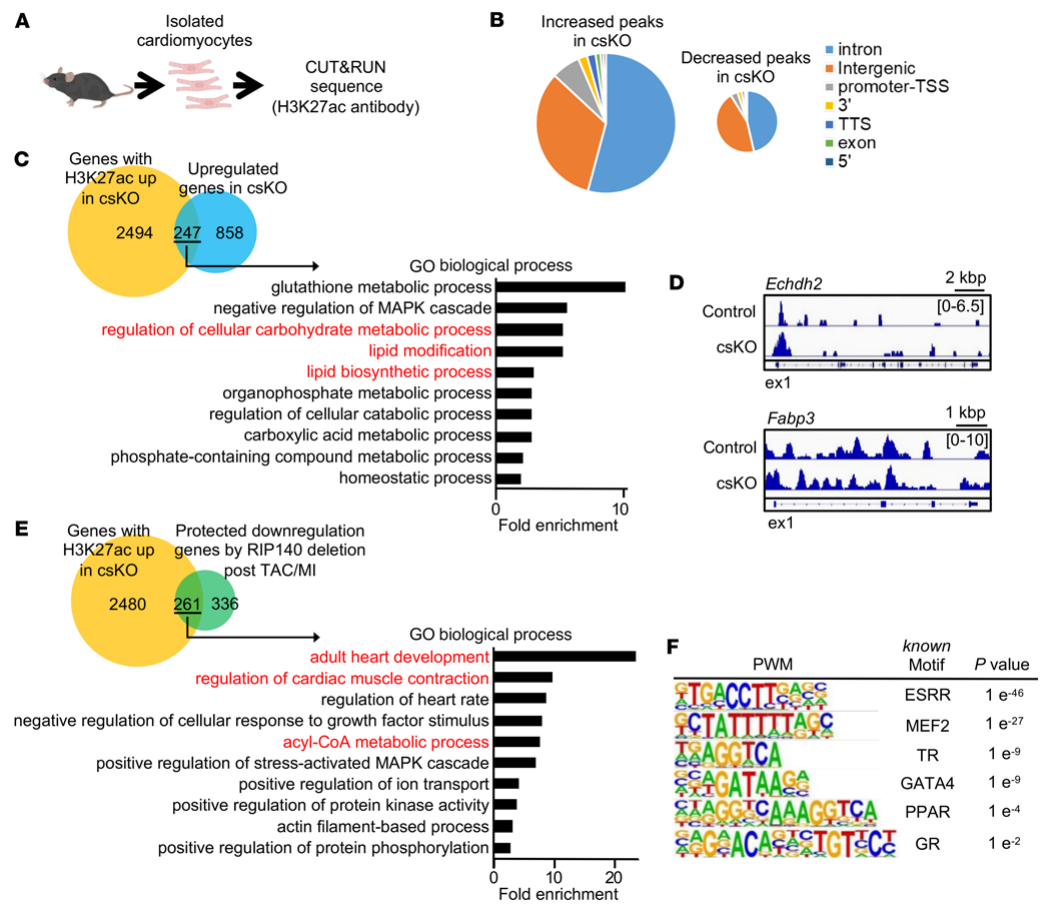
Identification of candidate RIP140 target transcription factor effectors via enhancer interrogation. (**A**) CUT&RUN sequencing was performed on CMs isolated from 10- to 12-week-old male cs*Nrip1^–/–^* and littermate WT control mice. (**B**) Pie chart of the genomic distribution of H3K27ac binding peaks that were significantly increased or reduced in CMs from cs*Nrip1^–/–^* mice versus control CMs. The promoter region was defined as –1 kb to +100 bp from the transcription start site (TSS). TTS, transcription termination site. (**C**) Venn diagram demonstrates the overlap between annotated genes associated with increased H3K27ac deposition (H3K27ac up) in csKO CMs and genes with upregulated expression in csKO CMs based on RNA-Seq. Graph shows GO biological process pathways enriched in the 247 overlapping genes. (**D**) Representative genome browser tracks of H3K27ac deposition peaks on selected target genes in control and cs*Nrip1^–/–^* samples. Numbers in brackets indicate reads per million (RPM). (**E**) Venn diagram demonstrates the overlap between annotated genes associated with increased H3K27ac deposition (H3K27ac up) in csKO CMs and genes that were protected (as defined in the text) against downregulation in csKO CMs following TAC/MI. Graph shows GO biological process pathways enriched in the 261 overlapping genes. (**F**) H3K27ac binding motifs as defined by position weight matrix motif analysis of H3K27ac binding sites that exhibited increased deposition with csKO.

We next sought to identify downstream target transcription factor effectors of RIP140 in CMs. To this end, we performed cleavage under targets and release using nuclease (CUT&RUN) sequencing on adult CMs isolated from hearts of cs*Nrip1^–/–^* mice and cs*Nrip1*^+/+^ littermate controls using immunoprecipitation with an H3K27ac antibody to focus on enhancer regions (Figure 6A). For this analysis, we focused on regions in which the level of H3K27ac deposition was significantly altered in cs*Nrip1^–/–^* versus control CMs. The number of H3K27ac peaks that were increased in RIP140-null CMs (*n* = 2,494) was substantially higher than that of decreased peaks (*n* = 858) (Figure 6B), consistent with a role as a transcriptional corepressor. Genes nearest the H3K27ac-increased peaks were subjected to Gene Ontology (GO) analyses, which revealed enrichment pathways for genes such as *Acacb*, *Cpt1a*, *Prkn*, *Fabp3*, *Acsm5*, and *Echdc2* involved in metabolic processes including lipid metabolism (Figures 6C and Supplemental Table 11; representative genomics viewer tracks, Figure 6D). Similar analyses were conducted for the genes that were protected by RIP140 deficiency in the TAC/MI data set. The latter analysis (Figure 6E and Supplemental Table 12) revealed genes involved in processes such as muscle contraction (*Tcap*, *Kcnj2*, *Adra1a*, and *Adrb1*) and fatty acid metabolism (acyl-CoA metabolic processes including *Acot7*, *Gpam*, *Dgat2*, and *Suclg2*). Motif analysis of peaks with increased H3K27ac deposition in cs*Nrip1^–/–^* CMs revealed strong enrichment of ERR–binding site genes, as has been shown previously (20–22), and the cardiogenic factor MEF2 (Figure 6F). Additional sites for nuclear receptors (thyroid hormone receptor [TR], PPAR, glucocorticoid receptor [GR]) and the cardiogenic factor GATA-binding protein 4 (GATA4) were also identified, although with significantly less binding site enrichment (Figure 6F). These data suggest that a potential network of transcription factor RIP140 targets involved in both energy metabolism (ERR, PPAR, TR) and cardiac sarcomeric function (MEF2, GATA4) were more active in the *Nrip1* hearts following TAC/MI.

### Increased FAO and storage dynamics in RIP140-deficient hearts under basal conditions and following chronic pressure overload.

**Figure 7:**
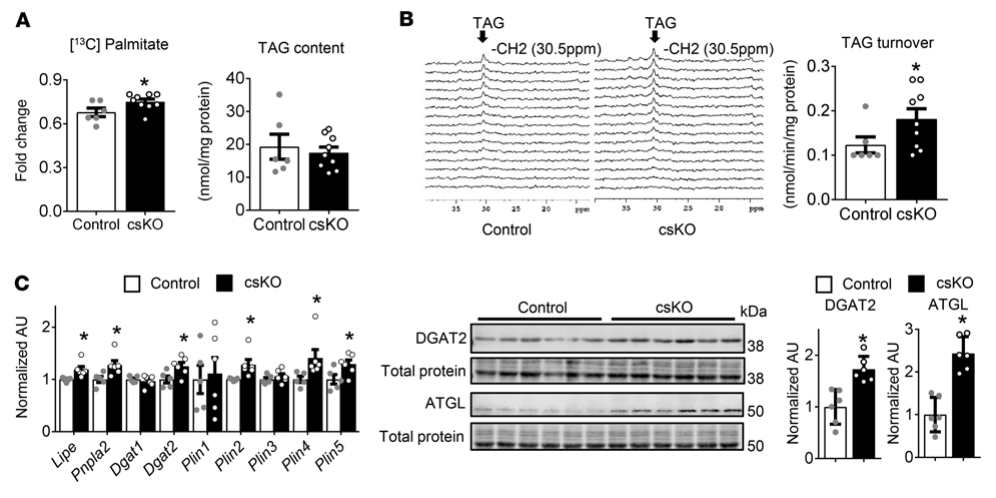
RIP140-deficient mouse hearts exhibit enhanced palmitate utilization and TAG turnover. (**A**) Fractional enrichment of acetyl-CoA from ^13^C-labeled palmitate into the TCA cycle (Fc) is shown in control and cs*Nrip1^–/–^* isolated perfused mouse hearts (16- to 18-week-old male littermates) (*n* = 6–9 per group) and TAG content in control and csKO hearts (*n* = 6–9 per group). (**B**) Representative selected ^13^C NMR spectra (from bottom to top, 2-min acquisition each) from an individual isolated heart perfused with ^13^C palmitate. Graph shows turnover rates of the intramyocardial TAG pool in control and csKO hearts (*n* = 6–9 per group). (**C**) Expression of TAG-remodeling genes (left) and protein levels (right) in 8 week-old male control and csKO hearts (*n* = 5–6 per group). Values are the mean ± SEM. *P < 0.05 versus control, by 2-tailed, unpaired Student’s *t* test.

The genomic interrogation of cs*Nrip1^–/–^* hearts demonstrated that genes involved in fatty acid utilization pathways were significantly less downregulated compared with controls (Supplemental Figure 6). ^13^C–NMR studies were conducted to assess fatty acid metabolism in the cs*Nrip1^–/–^* hearts. For these studies, the mouse hearts were perfused in the Langendorff mode with ^13^C-palmitate in a buffer containing a physiological substrate mix as previously described (23). The contribution of palmitate to carbon entry into the TCA cycle was significantly greater in cs*Nrip1^–/–^* hearts than in littermate control hearts, with no difference in TAG levels (Figure 7A). Notably, TAG turnover, a substantial source of fatty acids for both the substrate for mitochondrial β-oxidation and the ligand for PPARα activation (24), was significantly increased in the cs*Nrip1^–/–^* mice, consistent with increased fatty acid utilization (Figure 7B). We found that entry of anaplerotic carbon flux into the TCA cycle was unchanged by RIP140 deletion (data not shown). Consistent with the observed increase in TAG turnover rates, the expression of genes and proteins involved in TAG and lipid droplet remodeling, including *Lipe*, *Pnpla2*, *Dgat2*, and *Plin*, was increased in cs*Nrip1^–/–^* mice (Figure 7C). These latter results are of interest, given that *Pnpla2* (ATGL), a PPAR target, has been previously shown to play a critical role in TAG homeostasis in the heart (24, 25). Taken together, these results are consistent with the gene expression data indicating that RIP140 deficiency derepresses cardiac genes involved in lipid utilization pathways including genes for TAG hydrolysis and FAO.

**Figure 8:**
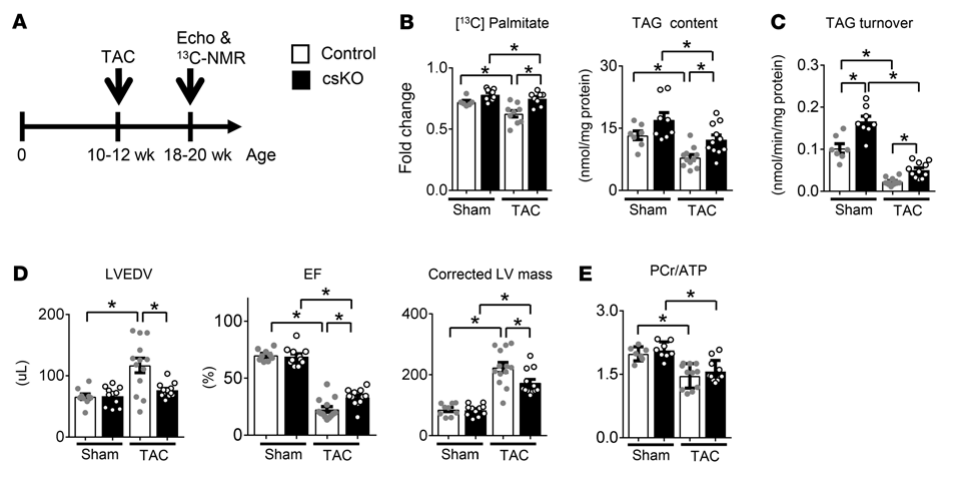
Cardiac-specific RIP140 deletion ameliorates impaired palmitate oxidation, TAG turnover, and LV remodeling induced by TAC surgery. (**A**) Schematic depicting the experimental timeline. Ten- to 12-week-old male mice were subjected to TAC to induce HF. Eight weeks after TAC surgery, echocardiography and ^13^C-NMR were performed on cs*Nrip1^–/–^* and WT littermate control mice (*n* = 7–11 per group). (**B**) Fractional enrichment of acetyl-CoA (Fc) and TAG content (*n* = 7–11 per group). (**C**) TAG turnover rates of the intramyocardial TAG pool in control and csKO hearts after TAC surgery (*n* = 7–11 per group). (**D**) LVEDV, EF, and corrected LV mass (*n* = 9–13 per group). Values are the mean ± SEM. (**E**) PCr/ATP ratio in control and csKO hearts after TAC surgery (*n* = 7–11 per group). *P < 0.05, by 2-way ANOVA with Tukey’s multiple-comparison test.

^13^C-NMR studies were repeated 8 weeks after a TAC protocol that produces cardiac remodeling and ventricular dysfunction (Figure 8A). As we reported previously (23, 25, 26), TAG content, TAG turnover, and long-chain FAO were all significantly decreased by TAC in the WT control group (Figure 8, B and C). In contrast, compared with controls, the RIP140-deficient hearts exhibited augmented TAG content and turnover, together with increased oxidation of palmitate following TAC (Figure 8, B and C). Consistent with the results of the PWAB and TAC/MI studies, the degree of LV hypertrophy, dilatation, and dysfunction (EF percentage) was significantly lower in the TAC-treated cs*Nrip1^–/–^* hearts (Figure 8D and Supplemental Table 13).

Despite the increased work exerted by the *Nrip1* hearts compared with control hearts, the ratio of phosphocreatine (PCr)/ATP levels, as measured by NMR, were similarly reduced in both groups after TAC (Figure 8E), consistent with the known reduced bioenergetic potential in pathologically stressed hearts (23, 26, 27). These results demonstrate that, although the steady-state energy reserve was compromised, ATP production and utilization remained sufficient to support the elevated function of the stressed *Nrip1* hearts.

### CM Nrip1 expression is regulated during perinatal development and in the failing heart by an ERRγ-mediated feedback mechanism.

Given the impact of RIP140 deficiency on pathologic cardiac remodeling, we sought to determine the expression of *Nrip1* in the failing heart. To this end, we assessed *Nrip1* expression in cardiac tissue from the PWAB and TAC/MI studies. *Nrip1* transcript levels were downregulated in both PWAB (Supplemental Figure 10A) and TAC/MI (Supplemental Figure 10B) LV tissues compared with sham-operated control samples. These results were corroborated in published human RNA-Seq data sets comparing failing and control normal-functioning hearts (https://zenodo.org/record/4114617#.YWrnTNnMJ0w) (28). Specifically, *NRIP1* levels were decreased in myocardial samples from patients with HF with reduced EF (HFrEF) as well as from those with HFpEF compared with expression levels in the respective control samples (Supplemental Figure 10C).

The results of the expression of *Nrip1* and *NRIP1* in failing hearts suggested that the expression of this may be regulated in accordance with the expression and activity of its downstream target ERR, as previously shown in adipocytes (29). Indeed, we found that *Nrip1* expression paralleled the known induction of *Esrrg* (4) after birth in mouse heart coincident with the known perinatal mitochondrial biogenesis response and switch to oxidative fuel metabolism (Supplemental Figure 10D). In addition, overexpression of ERRγ in human induced pluripotent stem cell–derived CMs (hiPSC-CMs) induced the expression of *NRIP1* (Supplemental Figure 10E). Last, we identified a highly conserved ERRγ occupation site within an intron between noncoding 5′ exons of *NRIP1* in the ERRγ cistrome (Supplemental Figure 10F) defined previously (4). Taken together, these results indicate that *NRIP1* expression was regulated in parallel with changes in CM oxidative capacity, driven at least in part by a feedback transcriptional regulation mediated by ERRγ.

## Discussion

PGC-1 and the downstream effectors ERR and PPAR comprise an inducible transcriptional regulatory circuitry that maintains mitochondrial oxidative capacity in tissues with high energy demand such as heart and skeletal muscle. Previous strategies to modulate this transcriptional regulatory network in vivo have depended largely on artificial overexpression strategies in mice. Although these approaches provide a mechanistic proof of concept that these factors regulate cardiac mitochondrial biogenesis and maturation, they have not conferred orchestrated activation of the components in this network within a physiological range of activity. Here, we describe the impact of releasing endogenous suppression on this circuitry by targeting the transcriptional corepressor RIP140 in mouse hearts. The results of releasing this transcriptional regulatory “brake” were dramatic and included protection against the development of pathologic cardiac hypertrophy and HF.

Global RNA-Seq conducted on cs*Nrip1^–/–^* hearts demonstrated an upregulation of a broad array of genes involved in mitochondrial fuel metabolism and OXPHOS. Similar results were observed in skeletal muscle in the str*Nrip1^–/–^* mice. In addition, we found that the expression of a subset of adult cardiac contractile genes was upregulated in the hearts of the str*Nrip1*-null mice. The increased expression of genes involved in cardiac fatty acid metabolism, including TAG turnover and FAO genes, prompted us to measure fatty acid and TAG metabolism using ^13^C-labeled palmitate via NMR in isolated *Nrip1* and WT control hearts. Consistent with the gene expression profile, we found that fatty acid utilization and TAG turnover dynamics were increased in the RIP140-deficient heart. The RNA-Seq data also demonstrated upregulated expression of genes involved in electron transport/OXPHOS and BCAA degradation in the *Nrip1* heart, although flux through these pathways was not directly measured. Taken together, these results strongly suggest that the capacity for mitochondrial fatty acid utilization and ATP synthesis was increased in the RIP140-deficient heart.

The effect of RIP140 deficiency on cardiac remodeling and function in the context of chronic pathophysiological drivers of HF proved very interesting. Both striated and cardiac-specific *Nrip1* mouse lines exhibited mild cardiac hypertrophy at baseline with normal function and an absence of histologic or gene markers of pathologic growth. These results suggested that this mild cardiac hypertrophic phenotype reflected a physiologic form of growth such as occurs during development or with exercise training. To this end, we probed known hypertrophic signaling pathways in the left ventricles of cs*Nrip1^–/–^* mice and found that mTORC1 signaling was markedly activated. These results are of interest, given that mTORC1 signaling has been implicated in physiologic forms of cardiac hypertrophy (16, 17). S6 kinase (S6K) regulates protein synthesis and is required for ribosome biogenesis downstream of mTORC1 (30). It is tempting to speculate that the adaptive hypertrophy of the csRIP140 heart exerts a degree of cardioprotection similar to that of the exercise-trained heart, though additional studies will be necessary.

Both RIP140-deficient mouse lines exhibited resistance to the development of pathological ventricular remodeling in 2 independent LV pressure overload models. RNA-Seq analysis demonstrated that the canonical downregulation of genes involved in cardiac mitochondrial fuel metabolism and OXPHOS that occurs in the hypertrophied and failing heart was less pronounced in the RIP140-deficient heart. Moreover, the expression levels of a subset of adult contractile isoform genes were partially protected from downregulation. Conversely, genes involved in inflammation and fibrosis were significantly (FDR <0.05) less upregulated in the RIP140-deficient heart in the HF models. Importantly, TCA cycle flux in the intact, functioning heart is driven by the energy demands of the mechanical work output by the heart and has been shown to correlate closely to the rate pressure product (RPP) of normal and failing hearts, with lower TCA cycle flux rates in failing hearts commensurate with reduced RPP (25, 27). Therefore, the increased oxidation of long-chain fats in RIP140-deficient hearts subjected to TAC, compared with controls, represents a shift in substrate preference at a metabolic flux rate matched to the rate of increased mechanical work output. Thus, with TAC, RIP140-deficient hearts oxidized more fat than did control hearts with TAC, consistent with the metabolic gene expression profile. Taken together, these results indicate that RIP140 serves to coordinately repress a network of genes involved in fatty acid utilization, bioenergetics, and contractile proteins and that pathological cardiac remodeling can be ameliorated by maintenance of these adult CM energy metabolic and contractile gene programs.

The RIP140-deficient heart exhibited a remarkable resistance to LV pressure overload–induced cardiac hypertrophic growth. This noteworthy, given emerging evidence that a switch away from FAO to glycolysis is required for cardiac hypertrophy to ensue (3, 6, 31). Carbon flux from the glucose utilization pathway provides anabolic inputs for nucleotide synthesis and lipogenesis, among other biosynthetic pathways that are necessary for myocyte proliferation or hypertrophic growth. This concept is akin to the Warburg model of cancer proliferation (32). Recent studies have shown that maintenance of cardiac FAO in the context of LV pressure overload results in a reduction in cardiac hypertrophic growth and the development of HF (13, 33). We have previously shown that myocardial TAG turnover, the significant source of fatty acid substrate for mitochondrial FAO and the source of ligand for PPARα activation (24), is downregulated in pressure overload (25). Consistent with this conceptual model, we found that *Nrip1* hearts had increased TAG turnover and augmented contribution of palmitate to oxidative energy metabolism in the mitochondria under basal conditions and in the context of severe LV pressure overload.

We found that the expression of *Nrip1/NRIP1* was downregulated in the failing mouse and human heart. These results are different from those of a previous study showing that RIP140 protein expression levels were increased in the chronically infarcted rat heart (34). The basis for the discordant results is not clear, although they could reflect differences in species or HF models . Our results suggested that RIP140 expression was regulated in parallel with the state of mitochondrial oxidative capacity. In other words, the “brake” function of RIP140 was modulated in accordance with the corresponding levels of ERR and other RIP140 target transcription factors in various conditions of physiological stress. Consistent with this hypothesis, we found that *Nrip1* expression was induced in parallel with that of ERRγ following birth in mouse hearts, coincident with the known switch to a greater reliance on oxidative fuel metabolism. In addition, we demonstrate that ERRγ activated *NRIP1* transcription, suggesting a feedback mechanism to modulate *Nrip1* levels in parallel with the level of ERR signaling in CMs. Importantly, however, the RIP140-repressive function remained operative in the hypertrophied and failing heart, given the cardiac-protective phenotype of the cs*Nrip1^–/–^* mouse following PWAB and TAC/MI.

The gene-regulatory effects of RIP140 deactivation in heart and skeletal muscle were likely the result of both direct and indirect effects on downstream transcriptional regulators. Previous studies, largely based on overexpression strategies, have indicated that RIP140 interacts with a variety of transcription factors including the nuclear receptors ERR and PPAR (20–22). In some contexts, RIP140 may also serve as a coactivator (21, 35–38). However, none of the studies interrogating RIP140 targets have been conducted in cardiac or skeletal myocytes, nor have they been conducted in an unbiased manner. Ideally, RIP140 immunoprecipitation (ChIP) with massively parallel DNA Sequencing (ChIP-Seq) would be conducted on *Nrip1*^+/+^ and *Nrip1^–/–^* CMs to identify RIP140 occupied regions of chromatin and corresponding DNA binding motifs for candidate transcription factor targets of RIP140. However, the currently available RIP140 antibodies are not suitable for such studies. As an alternative, we conducted CUT&RUN studies using an H3K27ac antibody to identify RIP140-occupied enhancers by comparing peaks in *Nrip1^+/+^* and *Nrip1* CMs followed by DNA binding-site motif analysis. GO analysis of genes predicted to be associated with the enhancers was compared with that of the pathways that were “protected” by RIP140 deficiency in response to TAC/MI based on RNA-Seq analysis. This approach demonstrated significant overlap for processes involved in lipid metabolism and contractile function, further supporting the conclusion that RIP140 directly suppresses the expression of genes in these key pathways. Moreover, motif analysis of the DNA sequence of the enhancers that exhibited increased H3K27ac deposition in *Nrip1* CMs demonstrated very significant enrichment for ERR and MEF2 binding sites. These results provide evidence that the known RIP140-ERR interaction is relevant in the CM and identifies MEF2A/C as a putative RIP140 target that may be involved in the RIP140-mediated upregulation of adult contractile protein genes in the *Nrip1* heart. The potential role of MEF2A as downstream target of RIP140 is also consistent with its known role in the control of both contractile and energy metabolic genes in the heart (39). Additional motifs that were enriched in this analysis, albeit at less significant levels, included binding sites for the nuclear receptors PPAR, TR, and GR as well as the cardiogenic factor GATA4. These collective data suggest that RIP140 corepresses a network of CM transcription factors involved in energy metabolism and contractile function. However, our data do not distinguish between direct and indirect RIP140 targets.

Significant evidence indicates that derangements in mitochondrial fuel utilization and energetics contribute to the development of cardiac hypertrophy and HF (3, 6, 31). In addition, skeletal muscle energetics is similarly reprogrammed in patients with HF (40, 41). Current therapies for HF are not aimed at these energetic derangements. The concept of inhibiting an endogenous suppressor of the transcriptional regulatory network that controls mitochondrial and contractile function is appealing, given that it would modulate activity within a more physiological range. In addition, our results indicate that RIP140 likely modulates multiple components of the circuitry that controls mitochondrial fuel and respiration together with reactivation of the adult contractile program, making it an appealing therapeutic target.

## Methods

### General protocol for mouse studies.

Mouse studies were performed on male and female mice 3–17 weeks of age (see below for further detail). Mice were housed in a facility under a 12-hour light/12-hour dark cycle. Tissues were harvested between 11 am and 12 pm (4-hour-fasted). Prior to tissue removal, animals were anesthetized with pentobarbital (Sagent, 100 mg/kg). TAC combined with TAC/MI cardiac surgeries were performed to induce HF, and PWAB surgery was performed to induce hypertrophy, as described previously (15, 18, 42). Ultrasound examination of the cardiovascular system was noninvasively performed using a Vevo 2100 Ultrasound System (VisualSonics), as described previously (42).

### Generation of MCK-Cre–driven striated muscle, RIP140-deficient mice.

*Nrip1*-floxed (RIP140-floxed) mice (on a C57BL/6J background) were generated and provided by Zhenji Gan (National Resource Center for Mutant Mice, Model Animal Research Center of Nanjing University, Nanjing, China). Briefly, RIP140-floxed mice were generated via the CRISPR/Cas9 system. The *Nrip1* (RIP140) gene has 4 exons, with the ATG start codon in exon 4 and the TAA stop codon in exon 4. Cas9 mRNA, sgRNA, and donor template were coinjected into zygotes. The sgRNA directed Cas9 endonuclease cleavage in intron flanked by exons 3–4 (intron 3) and downstream of 3′-UTR to create a double-stranded break. This double-stranded break was repaired and resulted in loxP sites inserted into intron 3 and downstream of 3′-UTR, respectively, by homologous recombination. The RIP140-floxed mice were bred with C57BL/6NJ mice expressing Cre recombinase under control of the MCK promoter (12) (obtained from The Jackson Laboratory, stock no. 006475) to generate striated muscle–specific RIP140-deficient mice (str*Nrip1^–/–^*). Male or female littermate Cre-positive (str*Nrip1^–/–^*) and Cre-negative (littermate controls) mice were then group housed.

### Generation of Nkx2.5-Cre–driven cardiac-specific RIP140-deficient mice.

The *Nrip1*-floxed (RIP140) mice were bred with C57BL/6J mice expressing Cre recombinase under control of the Nkx2.5 promoter (43) (obtained from The Jackson Laboratory, stock no. 024637) to generate cardiac-specific RIP140-deficient mice (cs*Nrip1^–/–^*). Male or female littermate Cre-positive (cs*Nrip1^–/–^*) and Cre-negative (littermate controls) mice were then group housed.

### Mouse cardiac surgery.

TAC combined with TAC/MI surgeries were performed on 8-week-old male mice. Mice were anesthetized with ketamine (90 mg/kg)/xylazine (10 mg/kg)/acepromazine (2 mg/kg) and then intubated and ventilated (Harvard Apparatus). TAC was performed on anesthetized mice by first freeing the aortic arch by blunt dissection. A 7-0 silk suture was passed under the aorta. The suture was then tied around a blunt needle (26–27 gauge depending on the size of the animal) lying on the artery, and the blunt needle was removed to induce constriction. Second, to induce a small MI, the chest wall was retracted through the fourth intercostal space to clearly visualize the left ventricle and the left anterior descending artery (LAD). The left anterior descending branch (LAD) of the left coronary artery was ligated with a 8-0 nylon suture. Prior to analysis of all TAC/MI samples, the infarcted apical region was removed from the heart (and the corresponding cardiac tissue region was removed from the sham-operated controls). PWAB surgeries were performed on 21- to 28-day-old mice (weighing 7–13 g) that underwent ascending aorta constriction as described above (26 gauge needle).

To study palmitate oxidation and triglyceride turnover in the heart, mice underwent TAC or sham surgery at 10 to 12 weeks of age. Pathological cardiac hypertrophy was induced by TAC as described earlier (23, 44). Briefly, a titanium hemoclip measuring 0.015 inches in diameter was used to constrict the transverse aortic arch. Eight weeks after surgery, echocardiography (Vevo 2100) was performed to assess the degree of cardiac dysfunction as manifested by increased LV mass and impaired systolic and diastolic function.

### Mouse echocardiography.

Ultrasound examination of the left ventricle was performed at the Rodent Cardiovascular Phenotyping Core of the University of Pennsylvania (Cardiovascular Institute) using a Fujifilm VisualSonics Ultrasound System. Adult mice were anesthetized with an i.p. injection (0.05 mg/g) of 2% avertin (to maintain a heart rate [HR] close to 600 beats per minute or higher for the evaluation of LV systolic function). Hair was removed from the anterior chest using chemical hair remover, and the animals were placed on a warming pad in a left lateral decubitus position to maintain normothermia, which was monitored by a rectal thermometer. Ultrasound gel was applied to the chest. Care was taken to maintain adequate contact while avoiding excessive pressure on the chest. 2D long-axis and short-axis M-mode images were obtained. For echocardiography following PWAB surgery, in addition to systolic function, diastolic function–related parameters were evaluated using a modified 4-chamber view. Pulsed-wave Doppler was used to determine transmitral inflow velocities (E and A waves), and tissue Doppler was used to obtain mitral annular tissue velocities (e′ and a′ waves) following i.p. injection of zatebradine (hyperpolarization-activated cyclic nucleotide–gated [HCN] channel blocker, given in the range of approximately 80–100 μL injection [0.008 mg/gm] of 2 mg/mL stock to avoid volume overload). Zatebradine is short acting and is used for acute (acts within 2–5 minutes) reduction of the HR to 450–475 bpm (15%–25 % HR reduction compared with baseline) for better separation of E and A waves, which were continuously monitored visually for up to 5 minutes after zatebradine. No spontaneous death was observed during echocardiography after zatebradine administration, and mice with obvious systolic dysfunction were not subjected to zatebradine injection. After completion of the imaging studies, mice were allowed to recover from anesthesia and zatebradine (typically within 15 min of the last injection) and returned to their cages. Images were analyzed using Vevo Lab software (VisualSonics).

The infarct size was assessed visually in the parasternal long-axis view. The percentage of infarct area was measured in end-diastole using the following equation: percentage of infarct = (distance from apex to border zone of infarct [mm]/distance from apex to base [mm]) × 100. To measure the distance, a straight line was drawn using calipers in VisualSonics Echo Analysis Software from the mid apex to the mid base up to the mitral annulus and from the mid apex to the end of the border zone.

### CM isolation.

Mouse CM isolations were performed by the Langendorff-free method, as described previously (45). In brief, following mouse anesthetization, the inferior vena cava and descending aorta were cut, and the right ventricle was injected with EDTA buffer. The aorta was clamped, the heart was removed, and the left ventricle was injected with EDTA and perfusion and collagenase buffers. The heart tissue was then cut into small pieces and further dissociated by pipette. Stop buffer was added to halt collagenase digestion, and cells were passed through a 100 μm strainer. CMs were collected by gravity sedimentation and washed 3 times with perfusion buffer containing increasing concentrations of calcium. Cells were plated on laminin-coated tissue culture plates and allowed to adhere for 1 hour prior to imaging. Images were collected using a Nikon Eclipse Ts2 inverted microscope equipped with a Nikon DS-Fi3 camera. CM size was quantified using ImageJ (NIH) (46) by measuring cell width and length. The EDTA buffer consisted of 130 mM NaCl, 5 mM KCl, 0.5 mM NaH2PO4, 10 mM HEPES, 10 mM glucose, 10 mM 2,3-butanedione monoxime (BDM), 10 mM taurine, and 5 mM EDTA. The perfusion buffer contained 130 mM NaCl, 5 mM KCl, 0.5 mM NaH2PO4, 10 mM HEPES, 10 mM glucose, 10 mM BDM, 10 mM taurine, and 10 mM MgCl_2_. The collagenase buffer consisted of 0.5 mg/mL collagenase II, 0.5 mg/mL collagenase IV, and 0.05 mg/mL protease XIV, dissolved in perfusion buffer. The stop buffer contained 5% FBS in perfusion buffer.

### RNA isolation and qRT-PCR.

Total RNA was isolated using the RNeasy Mini Kit (QIAGEN) and the RNase-Free DNase Set (QIAGEN) according to the manufacturer’s instructions. cDNAs were synthesized using the Affinity Script cDNA Synthesis Kit (Agilent Technologies) with 0.5 μg total RNA. PCR reactions were performed using Brilliant III Ultra-Fast SYBR Green QPCR Master Mix (Agilent Technologies) on a QuantStudio 6 Flex Real-Time PCR System (Applied Biosystems) with specific primers for each gene. The primer sets are listed in Supplemental Table 14. The expression of target mRNAs was normalized by that of *Rplp0* (36B4). All experiments were repeated 3 times in either triplicate or quadruplicate.

### RNA-Seq library preparation and its sequencing.

RNA library preparations and Sequencing reactions were conducted at GENEWIZ. RNA samples were quantified using Qubit 2.0 Fluorometer (Life Technologies, Thermo Fisher Scientific), and RNA integrity was checked using Agilent TapeStation 4200 (Agilent Technologies). RNA-Seq libraries were prepared using the NEBNext Ultra RNA Library Prep Kit for Illumina following the manufacturer’s instructions (New England BioLabs [NEB]). Briefly, mRNAs were initially enriched with Oligo(dT) beads (NEB). Enriched mRNAs were fragmented for 15 minutes at 94°C. First-strand and second-strand cDNAs were subsequently synthesized. cDNA fragments were end repaired and adenylated at the 3′ends, and universal adapters were ligated to the cDNA fragments, followed by index addition and library enrichment by PCR with limited cycles. The sequencing library was validated on the Agilent TapeStation (Agilent Technologies) and quantified by using a Qubit 2.0 Fluorometer (Invitrogen, Thermo Fisher Scientific) as well as by qRT-PCR (KAPA Biosystems). The sequencing libraries were clustered on a single (2 lanes in 30-209309547) lane of a flowcell. After clustering, the flowcell was loaded onto the Illumina HiSeq instrument (model 4000 or equivalent) according to manufacturer’s instructions. The samples were sequenced using a 2 × 150 bp paired-end (PE) configuration. Image analysis and base calling were conducted using HiSeq Control Software (HCS). Raw sequence data (.bcl files) generated by the Illumina HiSeq were converted into fastq files and demultiplexed using Illumina’s bcl2fastq 2.17 software. One mismatch was allowed for index sequence identification.

### RNA-Seq analysis.

Expression of transcripts of the GRCm38 (mm10) genome was quantified using Salmon (47). Transcript expression was then summarized at the gene level, and DEGs were found using DESeq2 (48) with the ashr adaptive shrinkage estimator (49). Genes with a FDR below 0.05, when comparing conditions of interest, were selected as differentially expressed. Only those genes with at least a 1.2-fold expression change in any direction were considered.

### GO analysis.

GO term, including KEGG pathway, enrichment analysis was performed using the g:Profiler web service API (50). Terms with a statistically significant (adjusted *P* < 0.05 by the Benjamini-Hochberg FDR method) enrichment of greater than 2 were presented. Colored maps of KEGG pathways were made using R package pathview, version 1.34.0 (51).

### Data availability.

The sequencing data sets discussed in this publication have been deposited in the NCBI’s Gene Expression Omnibus (GEO) database as a SuperSeries (GEO GSE205317).

### Analysis of published human RNA-Seq data.

DeSeq2 (48) was used to calculate fragments per kilobase of transcript per million mapped reads (FPKM) values for *NRIP1* expression in the previously published RNA-Seq data set from right ventricular septal endomyocardial biopsies (28) (https://zenodo.org/record/4114617#.YWrnTNnMJ0w). The box plots to represent the FPKM of *NRIP1* for each healthy donor control, HFrEF, and HFpEF group were created with the seaborn package, version 0.11.2.

### ChIP-Seq analysis.

The ERRγ ChIP-Seq data set was obtained from GEO GSE113784 (4). ERRγ-specific peaks were called on pooled replicate data sets with Homer’s findPeaks (52) using ERRγ KO with default parameters. The ERRγ peaks at the *NRIP1* locus were visualized with Integrative Genomics Viewer 2.8.9 (53). JASPAR (http://jaspar.genereg.net/) (54) was used to determine putative ERR binding sites in the ERRγ peak on the *NRIP1* locus with the default setting. The conservation between species was confirmed with the UCSC Genome Browser on Human (GRCh38/hg38) (55).

### Quantification of mtDNA.

Genomic/mtDNA was isolated using QIAzol (QIAGEN), followed by back extraction with 4 mol/L guanidine thiocyanate, 50 mM sodium citrate, and 1 mol/L tris, and an isopropanol precipitation. mtDNA content was determined by SYBR green analysis (Stratagene). To this end, the levels of NADH dehydrogenase subunit 1 (*mt-Nd1*), coded in mtDNA, were normalized to the levels of lipoprotein lipase (*Ldl*), coded in genomic DNA.

### Immunoblot analysis.

Whole-tissue lysates or nuclear protein lysates were subjected to SDS-PAGE and transferred onto a nitrocellulose membrane as previously described (56). Nuclear protein lysate was prepared using NE-PER Nuclear and Cytoplasmic Extraction Reagents (Thermo Fisher Scientific). The binding of primary antibodies was detected by donkey anti-rabbit or mouse (IRDye 800 or 700 conjugates; LI-COR Biosciences) secondary antibodies (1:7,500) and scanned with a LI-COR Odyssey infrared imaging system or an Odyssey Fc (LI-COR Biosciences). REVERT Total Protein Stain Kits (LI-COR Biosciences) were used to stain the whole protein on the Western blotting membrane. The following antibodies were used: RIP140 (Santa Cruz Biotechnology, catalog sc-81370, dilution 1:200); ATGL (Cell signaling, catalog 2138, dilution 1:1,000); DGAT2 (Novus Biologicals, catalog MB100-57851, dilution 1:1,000); total OXPHOS (Abcam, catalog ab110413, dilution 1:100); and lamin A/C (Cell Signaling Technology, catalog 2032, dilution 1:1,000); MCAD antibody and LCAD antibody (provided by Arnold W. Strauss, University of Cincinnati College of Medicine, Cincinnati, Ohio, USA); monoclonal ANTI-FLAG M2 (MilliporeSigma, catalog F1804, dilution 1:1,000); TBP (D5C9H) XP rabbit (Cell Signaling Technology, catalog 44059, dilution 1:1,000); pS6 S235/S236 (Cell Signaling Technology, catalog 2211, dilution 1:1,000); S6 (Cell Signaling Technology, catalog 2217, dilution 1:1,000); 4E-BP1 (Cell Signaling Technology, catalog 9644, dilution 1:1,000); p4E-BP1 (Cell Signaling Technology, catalog 9451, dilution 1:1,000); and GAPDH (Cell Signaling Technology, catalog 2118, dilution 1:20,000).

### H3K27ac CUT&RUN in CM.

Adult CMs were isolated from 10- to 12-week-old mice by Langendorff perfusion. The CM fraction was enriched by low-speed centrifugation at 50*g* for 5 minutes. H3K27ac CUT&RUN was performed in CMs as previously described (57). A total of 200,000 CMs were used for each CUT&RUN experiment. H3K27ac (Cell Signaling Technology, catalog 8173) or rabbit IgG (Cell Signaling Technology, catalog 3900) at 1:100 was incubated with CMs, which were attached to concanavalin A–coated magnetic beads (Bangs Laboratories, catalog BP531) overnight at 4°C. pA-MNase (2.5 μL) from a 1:10 dilution of the original stock provided by the Steven Henikoff laboratory at the Fred Hutch Cancer Center was applied to the cell-bead mixture and incubated at room temperature for 10 minutes. Pull-down DNA was extracted in phase-lock tubes using the phenol-chloroform method. The DNA library was prepared using the Hyper-Prep kit (KAPA Biosystems KK8502) and submitted to the Next-Generation Sequencing Core at the University of Pennsylvania for sequencing. PE sequencing at 40 bp × 40 bp was adopted.

### CUT&RUN data analysis.

Reads were aligned to the GRCm38 reference genome with bowtie2, version 2.4.2 (58) using the command line parameters -N 1. Duplicate reads were removed with samtools rmdup (59). Further processing was performed with Homer, version 4.11.1 (52). Tag directories were created with Homer’s makeTagDirectory. H3K27ac-enriched regions were called for each biological replicate separately with Homer’s findPeaks, against the IgG background, using the parameters -style histone. Reliable H3K27ac-high regions were identified as those where enrichment was detected in at least 2 of 3 replicates, using Homer’s mergePeaks. Regions with differential H3K27ac occupation between the control and csRIP140 genotypes were called with Homer’s getDifferentialPeaks, taking the identified H3K27ac-high regions in control and KO and comparing CUT&RUN tag counts between pooled control and KO samples within them, with the parameters -F 1.5. Thus, a 1.5-fold difference between control and KO H3K27ac signal is required to call a region H3K27ac differential. The selected regions were annotated with nearest genes and genomic region kinds using Homer’s annotatePeaks.pl. DNA binding motifs enriched inside the selected regions were found with Homer’s findMotifsGenome.pl using the parameters -size 200 -S 10 -len 10,12,14,16. GO enrichment analysis with nearest genes was performed as described in the GO analysis subsection.

### Isolated heart perfusion experiments for NMR studies.

Euthanasia by cardiectomy was performed on heparinized (50 IU, i.p.), anesthetized mice (80 mg/kg ketamine plus 12 mg/kg xylazine, i.p.) for isolated heart perfusions as described previously (25). Hearts were perfused with a modified Krebs Henseleit buffer and oxygenated at 37°C. Buffer initially contained: 10 mmol/L glucose, 1.0 mmol/L lactate, 0.1 mmol/L pyruvate, and 0.4 mmol/L palmitate bound to BSA (3:1) to reach metabolic steady state during preparation for the NMR experiment (60, 61). Hearts spontaneously contracted against a fluid-filled intraventricular balloon connected to a pressure transducer and set to 5 mm Hg end-diastolic pressure to provide a workload. LV developed pressure, HR, and change in pressure/change in time (dP/dt) were recorded (PowerLab; AD Instruments). Perfusate was never recirculated. Hearts were situated in a 10 mm NMR probe within a 600 MHz wide-bore magnet. After stabilization, the perfusion media were switched to similar buffer containing 0.2 mmol/L [U-^13^C_16_]palmitate. A 2-minute ^31^P NMR spectrum was acquired to confirm a stable energetic state. After completion of each perfusion, hearts were rapidly frozen using liquid nitrogen–cooled tongs.

### TAG assays.

TAG extracts were prepared from perfused heart samples for liquid chromatography–mass spectrometry (LC-MS) analysis to determine TAG content as described previously with some modifications (25, 62). Briefly, approximately 10 mg frozen heart tissue was homogenized in 500 μL PBS, and 25 μL homogenate was added to a 16 × 100 mm glass culture tube with 5 μL deuterated TAG as an internal standard (LM6000, Avanti Polar Lipids). After addition of 2 mL chloroform/methanol (2:1), followed by the addition of 400 μL methanol, the extract was vortexed and placed on ice. After 30 minutes, the sample was centrifuged at 3,000 rpm (1,962*g*) for 10 minutes. The pellet was discarded, and 400 μL 4% CaCl_2_ was added to the supernatant, which was then centrifuged at 2,000 rpm (872*g*) for 10 minutes. The lower phases were washed 3 times with 1 mL pure solvent (chloroform/methanol/water, 1.5:24:23.5). The lower phase was collected and dried in a RapidVac (Labconco Corporation). The extracted lipid was reconstituted with 500 μL 2-propanol/methanol/heptane (45:45:10), and the TAG concentration and fractional ^13^C enrichment of the long-chain acyl groups were assessed by neutral loss LC-MS analysis according to previously published methods (62). LC-MS was performed using Vanquigh UHPLC+ and TSQ Altis (Thermo Fisher Scientific), and samples were separated on a NUCLEODUR 100-3 C8 ec column (Macherey-Nagel) at a flow rate of 0.3 mL/min at 40^o^C. Mobile phases consisted of solvent A (8:1:1 acetonitrile/2-propanol/water, 10 mmol/L ammonium acetate) and solvent B (2-propanol, 10 mmol/L ammonium acetate) with the following gradient: 0 minutes 35% B, 3 minutes 35% B, 18 minutes 50% B, 23 minutes 65% B, 24 minutes 65% B, 25 minutes 35% B, and 28 minutes 35% B.

### TAG turnover in functioning hearts.

The incorporation of ^13^C palmitate into the steady-state TAG pool was assessed according to ^13^C-NMR detection of the TAG methylene resonances at 30.5 ppm, as described previously (25–27, 63, 64). A biphasic exponential fit of dynamic ^13^C-NMR data combined with ^13^C endpoint enrichment and total TAG content in myocardium measured via LC-MS enabled quantification of the TAG turnover rate.

### In vitro NMR spectroscopy of myocardial extracts.

The fractional contribution (Fc) of ^13^C palmitate to acetyl–coenzyme A (acetyl-CoA) entering the TCA cycle was determined as described previously from glutamate isotopomer and isotopolog analysis to detect relative multiplet signals within the NMR resonance signals from the glutamate 3 and 4 carbons, as previously described (26, 27, 63, 64).

### hiPSC culture system.

hiPSCs (α-Skin), provided by the laboratory of Huei-Sheng Vincent Chen (Indiana University School of Medicine, Indianapolis, Indiana, USA), were cultured in TeSR-E8 (Stem Cell Technologies, catalog 05990). CDM3 hiPSC-CM differentiation (65, 66) was conducted with minor modifications as previously described (5). Adenoviral infection was conducted after enriching hiPSC CMs with metabolic selection (5). Two days after the adenoviral infection, the hiPSC CMs were harvested to assess protein and gene expression.

### Adenoviral construct.

Adenovirus expressing the human *ESRRG* variant1 (NM_001438.3) with a FLAG tag was generated with pAdTrack-CMV and the AdEasy system (Agilent Technologies) as previously described (5).

### Statistics.

For 2-group comparisons, a Student’s *t* test was performed when the data were normally distributed based on a Shapiro-Wilk test (α = 0.05). Mann-Whitney *U* test was used to assess non-normally distributed data. For multiple comparisons, 2-way ANOVA with Tukey’s post hoc test was performed. We report the FDR as computed by the Database for Annotation, Visualization, and Integrated Discovery (DAVID), which performs the correction for each gene set (result table) separately. GraphPad Prism 7.04 or 8.03 (GraphPad Software) was used for graphing and statistical analysis.

### Study approval.

All animal studies were performed in accordance with NIH guidelines for the humane treatment of animals and approved by the IACUCs at the University of Pennsylvania and The Ohio State University.

## Author contributions

TY and DPK conceptualized the project. TY, SKM, EP, YX, SMS, TS, YW, LL, and TRK conducted experiments. All authors participated in scientific discussions including data analysis. KB specifically conducted bioinformatics analyses. KSM performed the cardiac surgeries, and SVS performed the echocardiography studies. TY and DPK wrote the manuscript. All authors edited and approved the manuscript.

## Supplementary Material

Supplemental data

## Version 1 03/16/2023

In-Press Preview

## Version 2 05/01/2023

Electronic publication
