# Supplementary material for: RIP140 deficiency enhances cardiac fuel metabolism and protects mice from heart failure
Source: J Clin Invest. 2023 May 1;133(9):e162309. doi: 10.1172/JCI162309 (PMC10145947; doi:10.1172/JCI162309)
Supplement: Supplemental data [file jci-133-162309-s043.pdf]

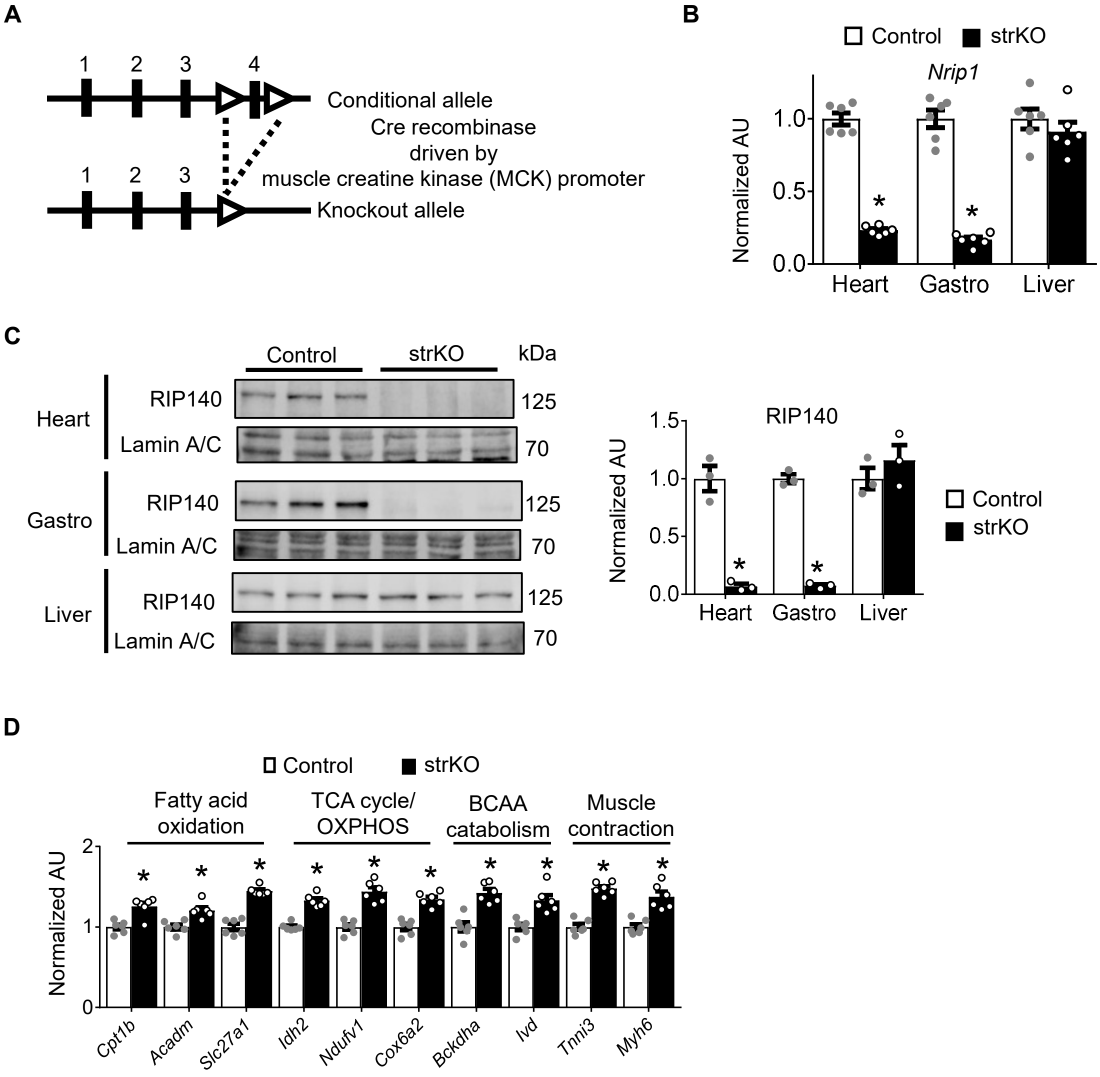

Supplemental Figure 1. Generation of striated muscle-specific RIP140-deficient mice (strKO). (**A**) Schematic diagram of conditional and knockout of *Nrip1* alleles in mice. Black boxes depict exons and white triangles denote LoxP sites. Targeted coding sequence is located in exon 4. (**B**) qRT-PCR analysis of *Nrip1* expression in heart, gastrocnemius (Gastro) and liver (n=6 per group). (**C**) Left; representative immunoblots of RIP140 and Lamin A/C (control) in 8-week old male heart, gastrocnemius, and liver. Right; bar graphs show the relative amount of RIP140 protein expression normalized (=1) to wild-type control littermates (Control) in Arbitrary Units (AU). (**D**) qRT-PCR analysis of representative DE genes shown as normalized to littermate control levels (n=6 per group). Values are shown as mean ± SEM. * $p < 0.05$ vs control using two-tailed, unpaired t-test.

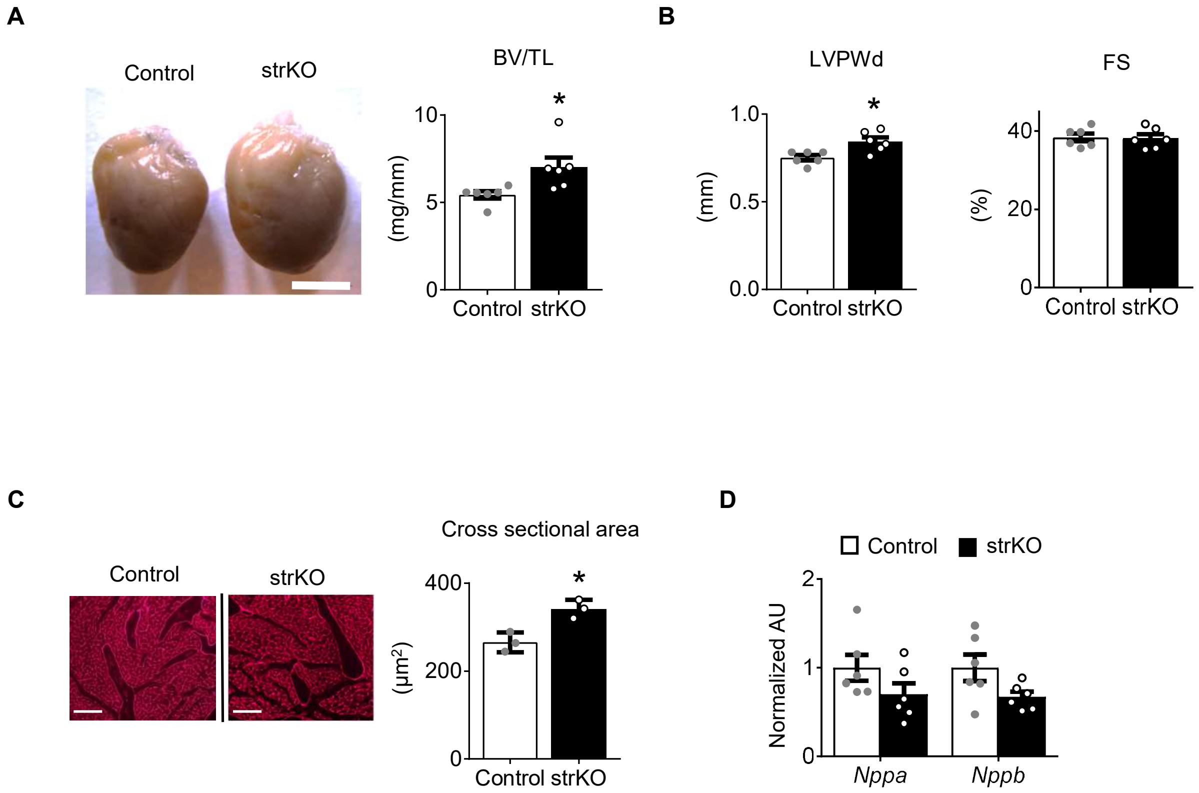

Supplemental Figure 2. str*Nrip1*$^{-/-}$ mice (strKO) exhibit mild cardiac hypertrophy with normal systolic function. (**A**) Left; representative gross biventricle photo for 8 week-old male mice. Scale bar = 5 mm. Right; biventricular weight (BV) to tibia length (TL) ratio (n=6 per group). (**B**) Left ventricular posterior wall thickness at end-diastole (LVPWd), and left ventricular fractional shortening (FS) determined by echocardiography in 8 week-old male (n=6 per group). (**C**) Left; representative image of heart sections stained with Wheat Germ Agglutinin (WGA). Right; quantification of cardiomyocyte size. Scale bars = 100 µm. (**D**) Expression of cardiac hypertrophic/stress markers, *Nppa* and *Nppb*, in 8 week-old male mice (n=6 per group). Values are shown as mean ± SEM. * $p < 0.05$ vs control using two-tailed, unpaired t-test.

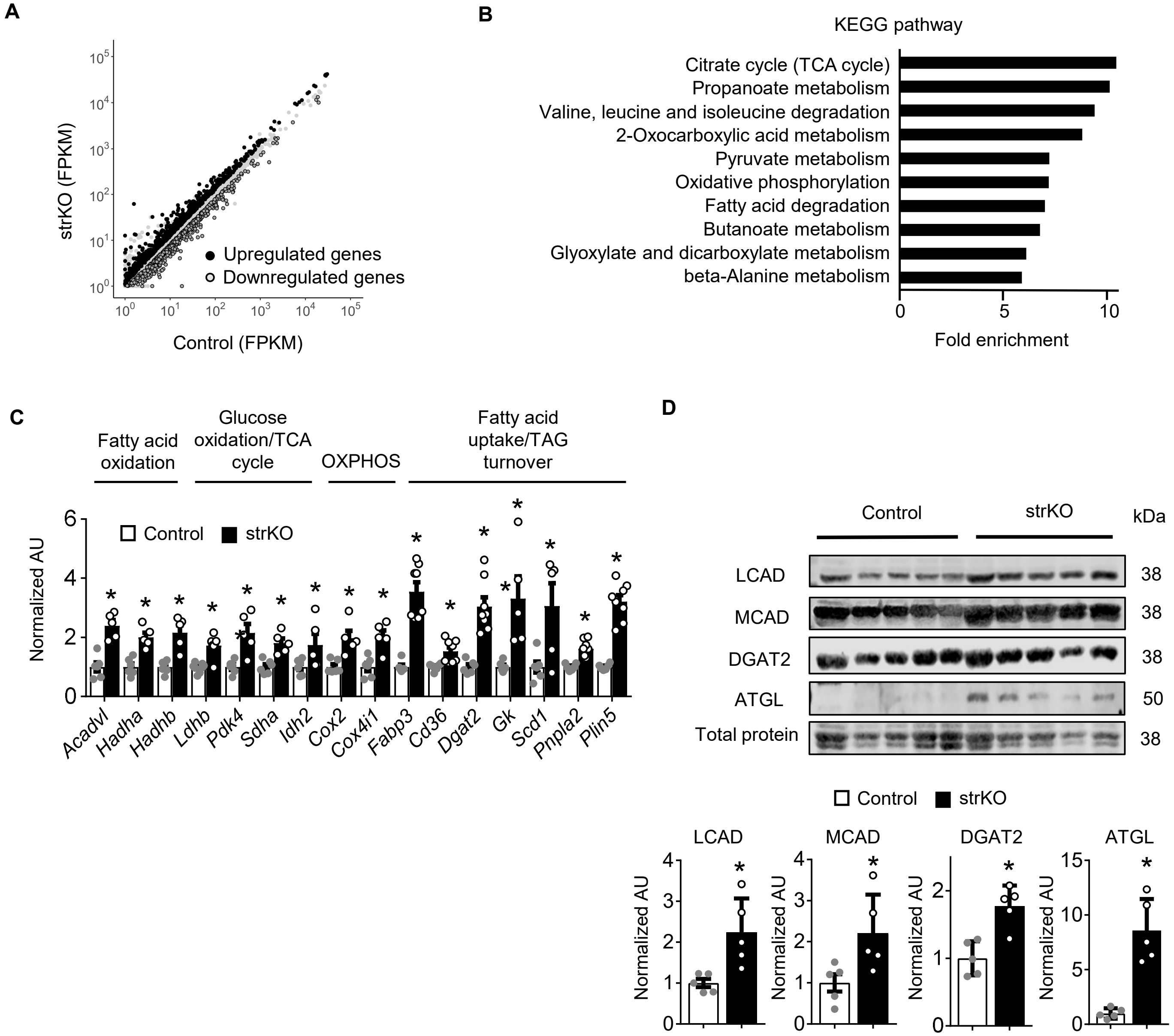

Supplemental Figure 3. Increased expression of a wide array of mitochondrial energy transduction genes in str*Nrip1*$^{-/-}$ skeletal muscle. Global RNA-seq was performed with wild-type littermate control and str*Nrip1*$^{-/-}$ (strKO) 8 week-old male gastrocnemius muscle (n=3 per group). (**A**) Scatter plot representing upregulated (black circles) or downregulated (grey circles) genes in strKO muscle vs control. Cutoff: FC>1.2 and FDR<0.05. (**B**) KEGG pathways enriched in strKO upregulated genes. (**C**) Validation of a subset of RNA-seq target genes by qPCR. (**D**) Top; representative immunoblots of fatty acid oxidation (LCAD and MCAD), triacylglyceride (TAG) synthesis (DGAT2), and hydrolysis (ATGL) genes in 8-week old male gastrocnemius. Bottom; Bar graphs demonstrating quantification of the immunoblots (n = 5 per group). Values are mean ± SEM. * $p < 0.05$ using unpaired t-test.

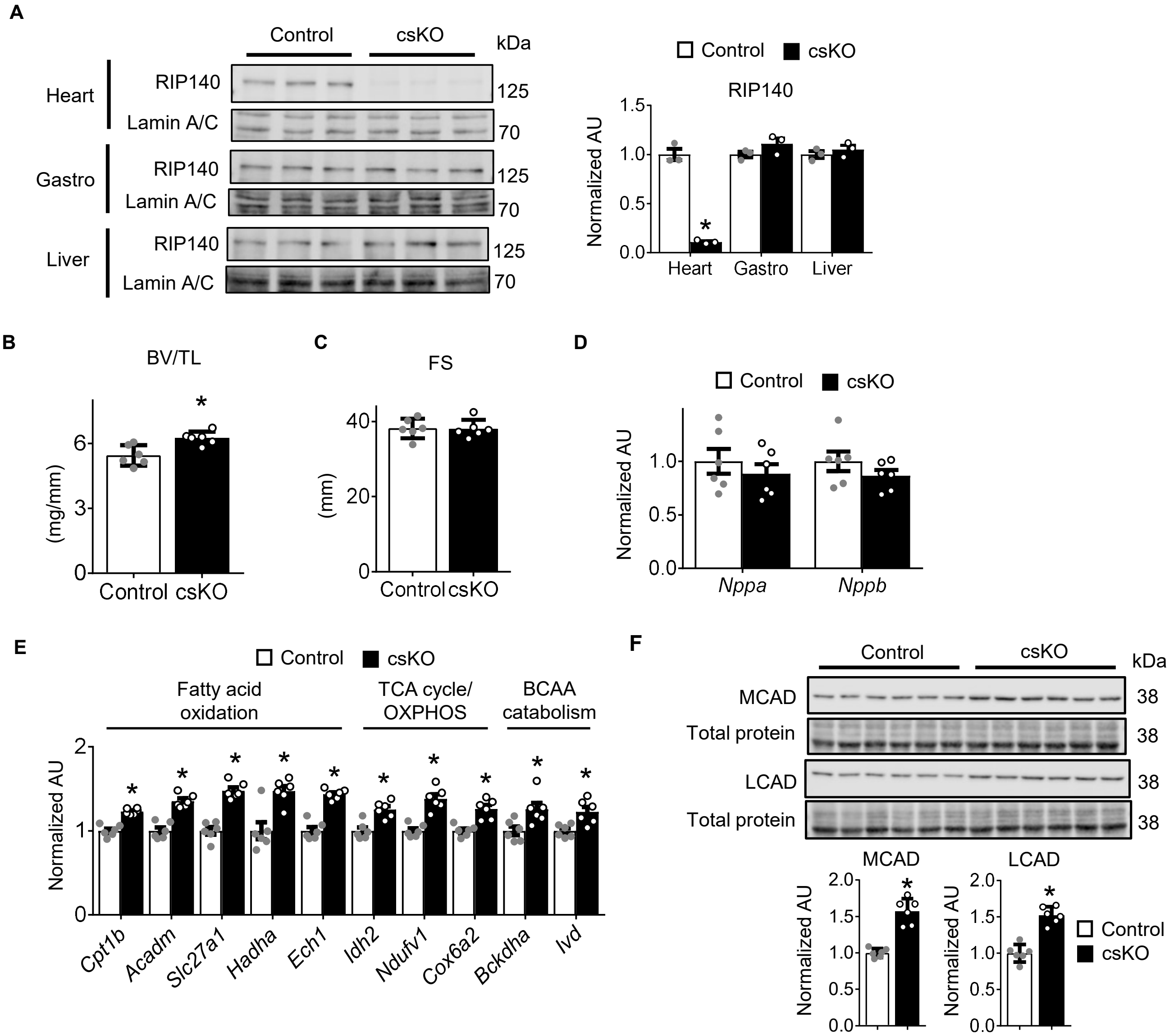

Supplemental Figure 4. Generation of cardiac-specific *Nrip1*$^{-/-}$ mice (csKO). (**A**) Left; representative immunoblots of RIP140 and Lamin A/C (control) performed on 8-week old male mouse heart, gastrocnemius (Gastro), and liver. Right; quantification of immunoblots (n=3 per group). (**B**) Biventricular weight (BV) to tibia length (TL) ratio (n=6 per group). (**C**) Left ventricular fractional shortening (FS) (n=6 per group). (**D**) Expression of cardiac hypertrophic/stress markers normalized to wild-type littermate control levels by qRT-PCR (n=6 per group). (**E**) qRT-PCR analysis of representative expression in heart (n=6 per group). (**F**) Top; representative immunoblots to assess MCAD and LCAD levels in biventricle. Bottom; quantification of immunoblots normalized to Control (=1). Values are mean ± SEM. * $p < 0.05$ vs control using two-tailed, unpaired t-test.

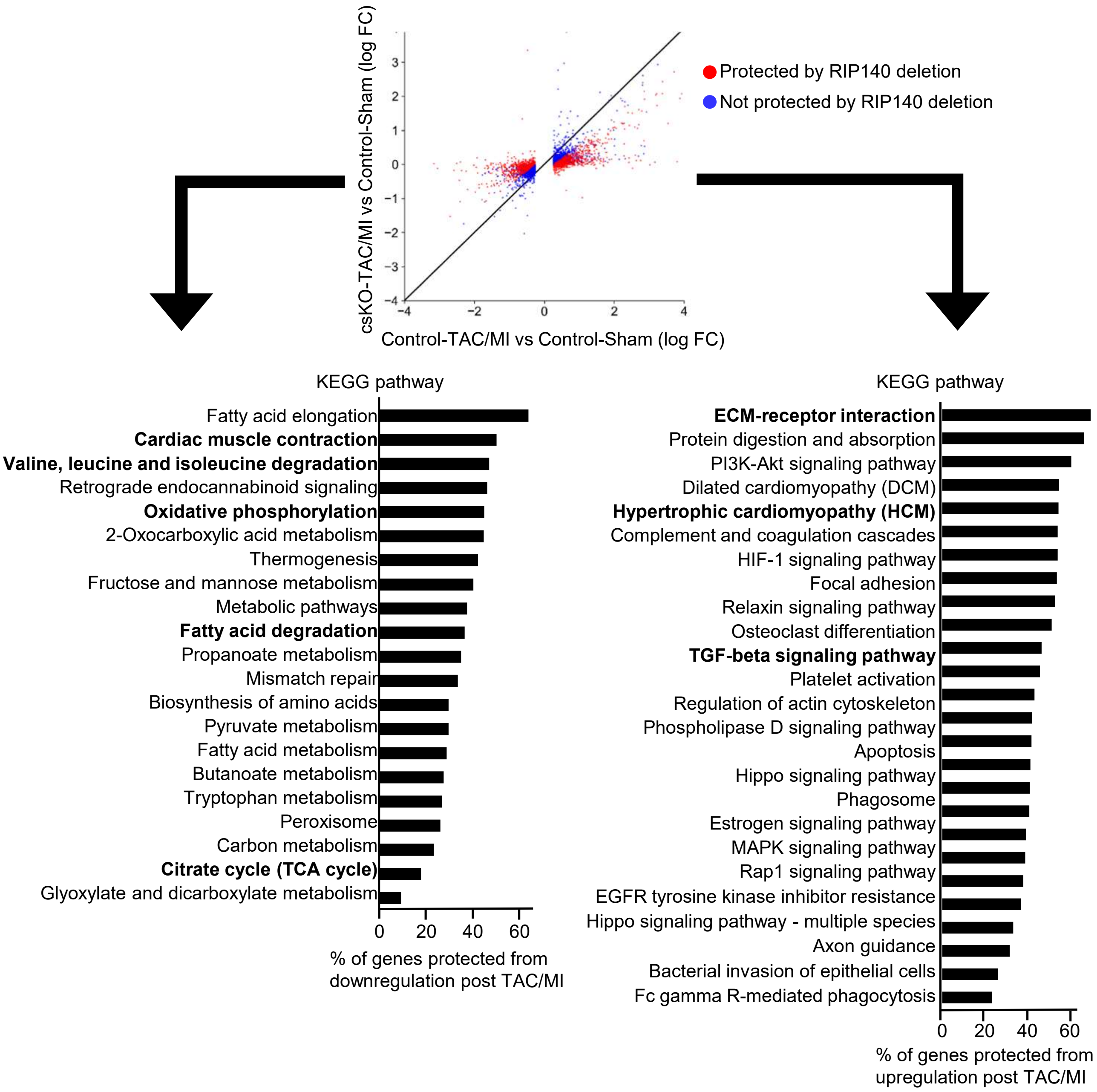

Supplemental Figure 5. Pathway analysis of the impact of cardiac RIP140 deficiency on differential expression of genes post-TAC/MI. "Protected" genes are defined as significantly less upregulated/downregulated post-TAC/MI in csKO vs Control both compared to Control-Sham using the cutoff: FC>1.2, FDR<0.05. Upper panel; scatter plot of genes protected (red) and non-protected (blue) by RIP140 deletion post-TAC/MI. Lower panel; KEGG pathways enriched in genes protected by RIP140 deletion post-TAC/MI. Bars show the percentage of genes in the pathway that were significantly affected by TAC/MI in Control genotype, which were protected from this change by RIP140 deletion. Bold: pathways discussed in the text.

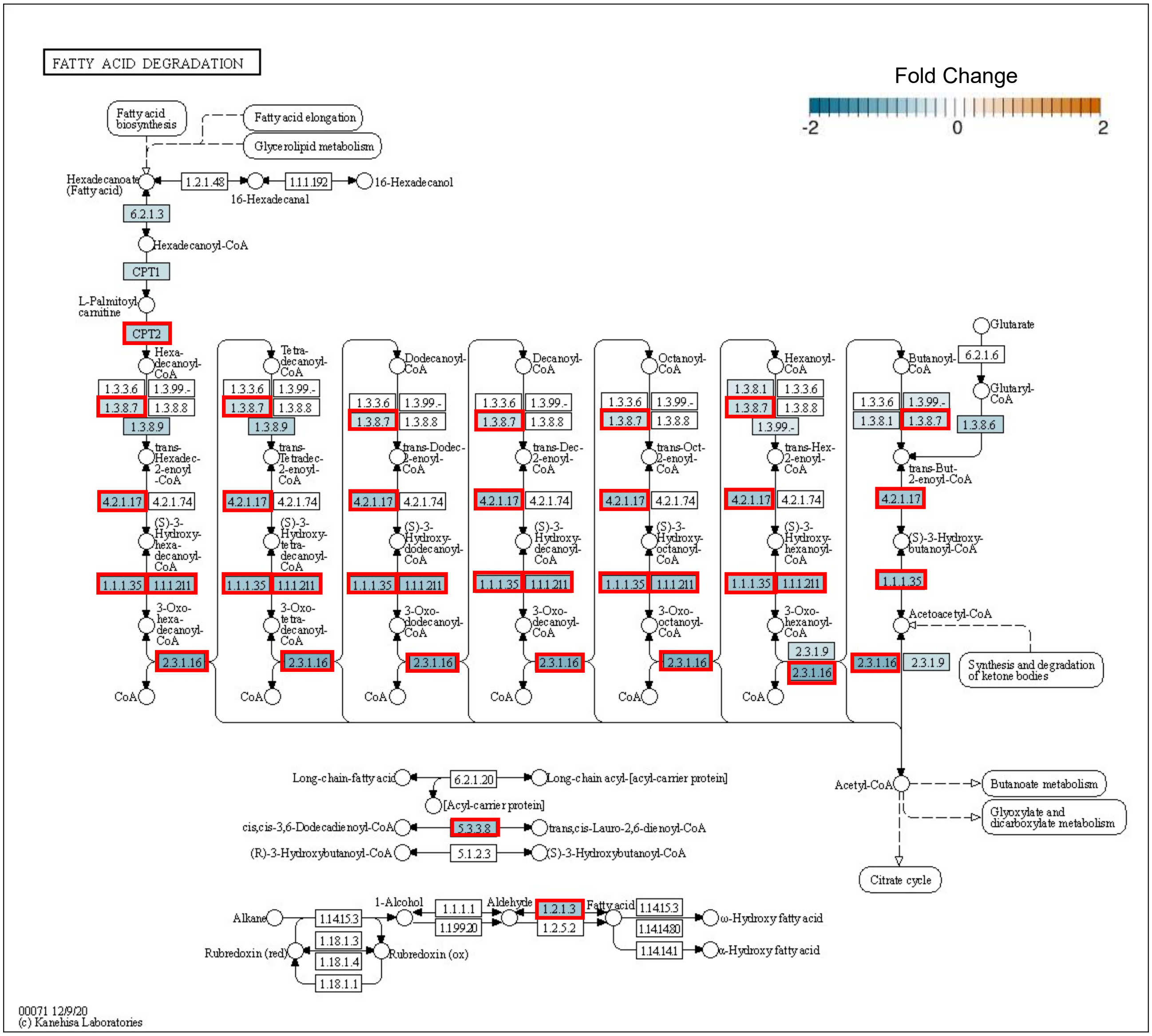

Supplemental Figure 6. Impact of RIP140 deficiency gene expression post-TAC/MI: Fatty acid degradation pathway (KEGG). Cardiac genes that were differentially expressed (DE) in sham vs. TAC/MI cs*Nrip1*$^{+/+}$ groups (FC>1.2 and FDR<0.05) are delineated by shading (see gradient key). Genes "protected" by RIP140 deletion (significantly less up/downregulated) are outlined in red.

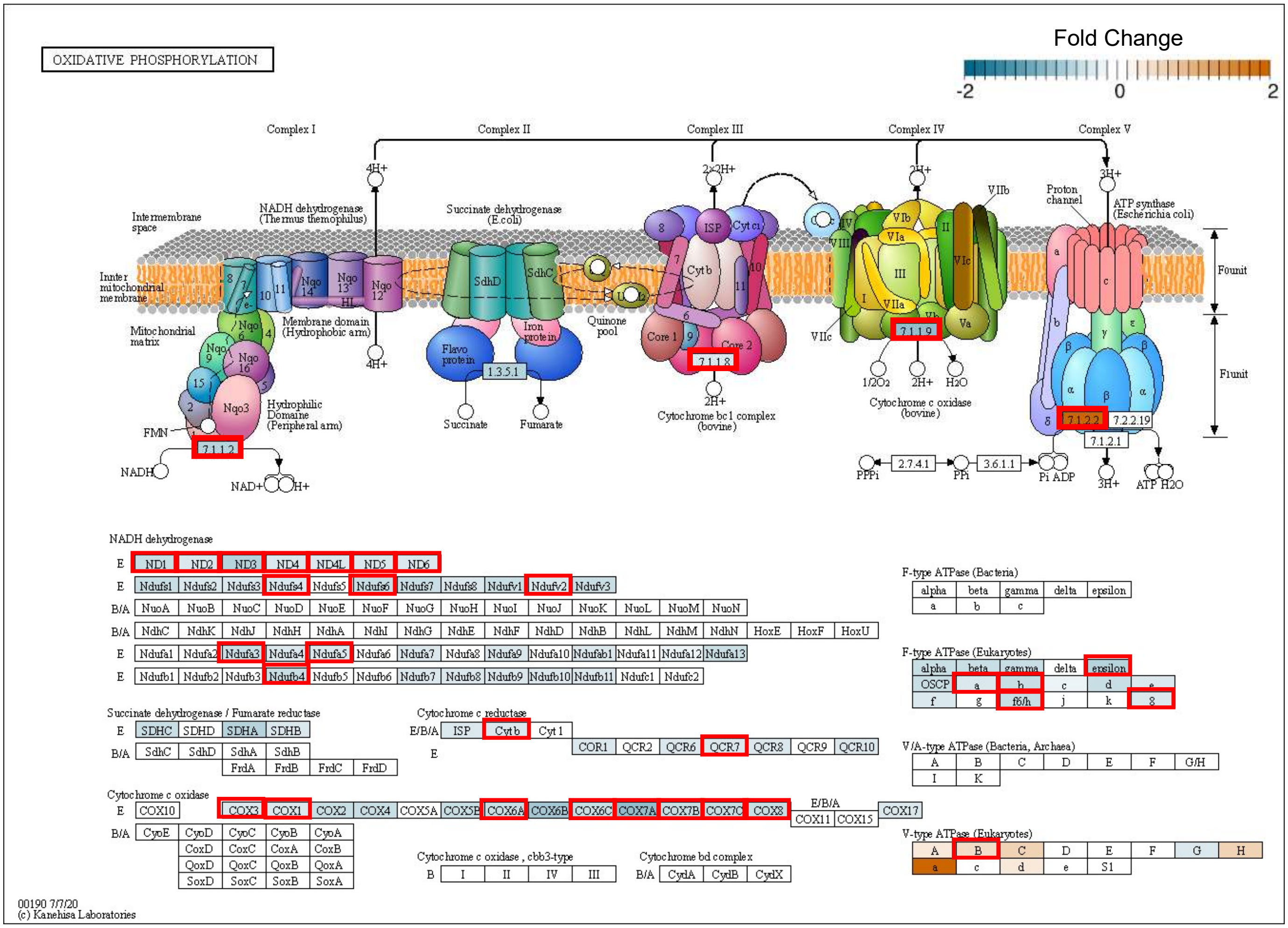

Supplemental Figure 7. Impact of RIP140 deficiency gene expression post-TAC/MI: Oxidative phosphorylation (OXPHOS) pathway (KEGG). Cardiac genes that were differentially expressed (DE) in sham vs. TAC/MI cs*Nrip1*$^{+/+}$ groups (FC>1.2 and FDR<0.05) are delineated by shading (see gradient key). Genes "protected" by RIP140 deletion (significantly less up/downregulated) are outlined in red.

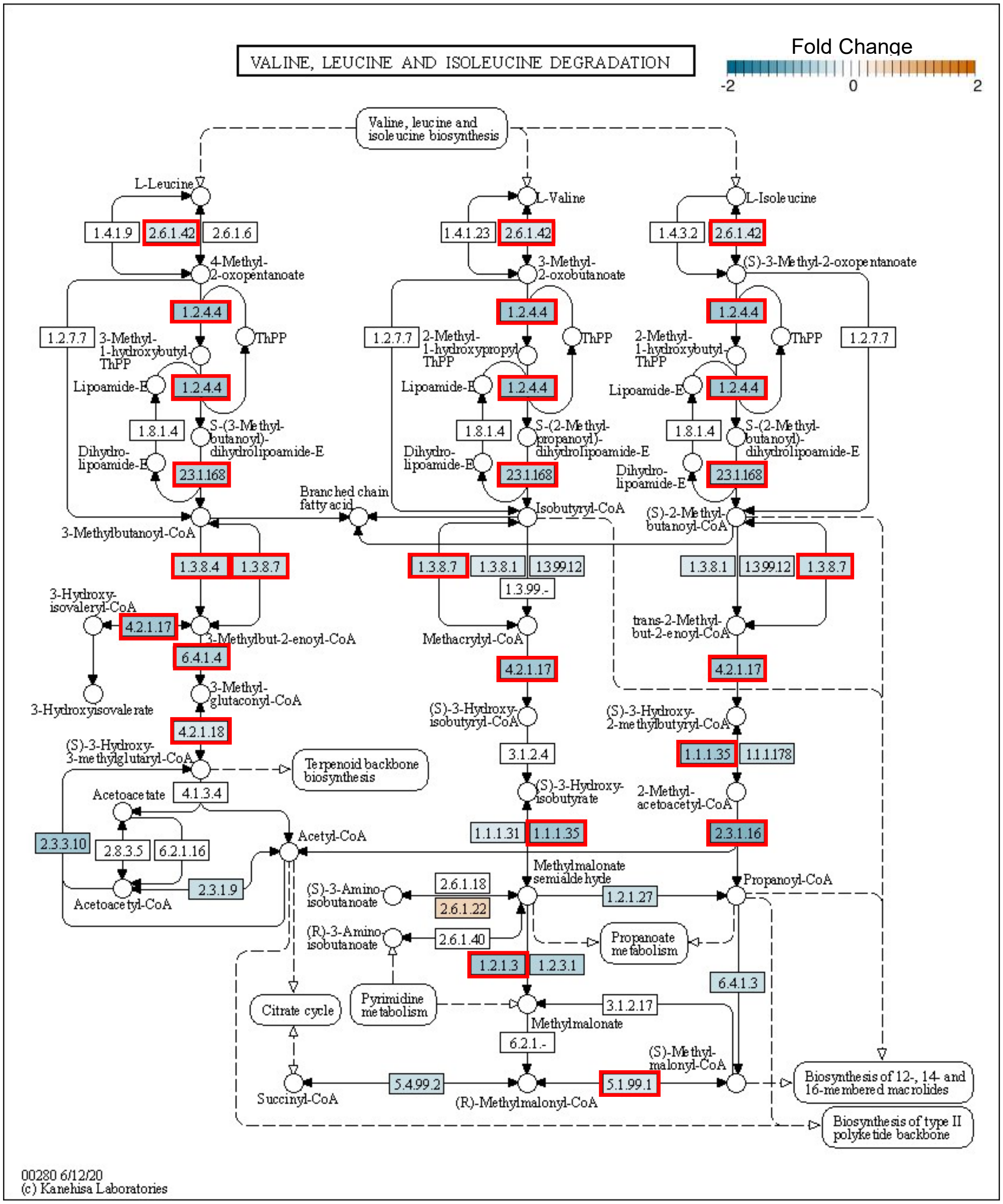

Supplemental Figure 8. Impact of RIP140 deficiency gene expression post-TAC/MI: branched chain amino acid degradation pathway (KEGG). Cardiac genes that were differentially expressed (DE) in sham vs. TAC/MI cs*Nrip1*$^{+/+}$ groups (FC>1.2 and FDR<0.05) are delineated by shading (see gradient key). Genes "protected" by RIP140 deletion, (significantly less up/downregulated) are outlined in red.

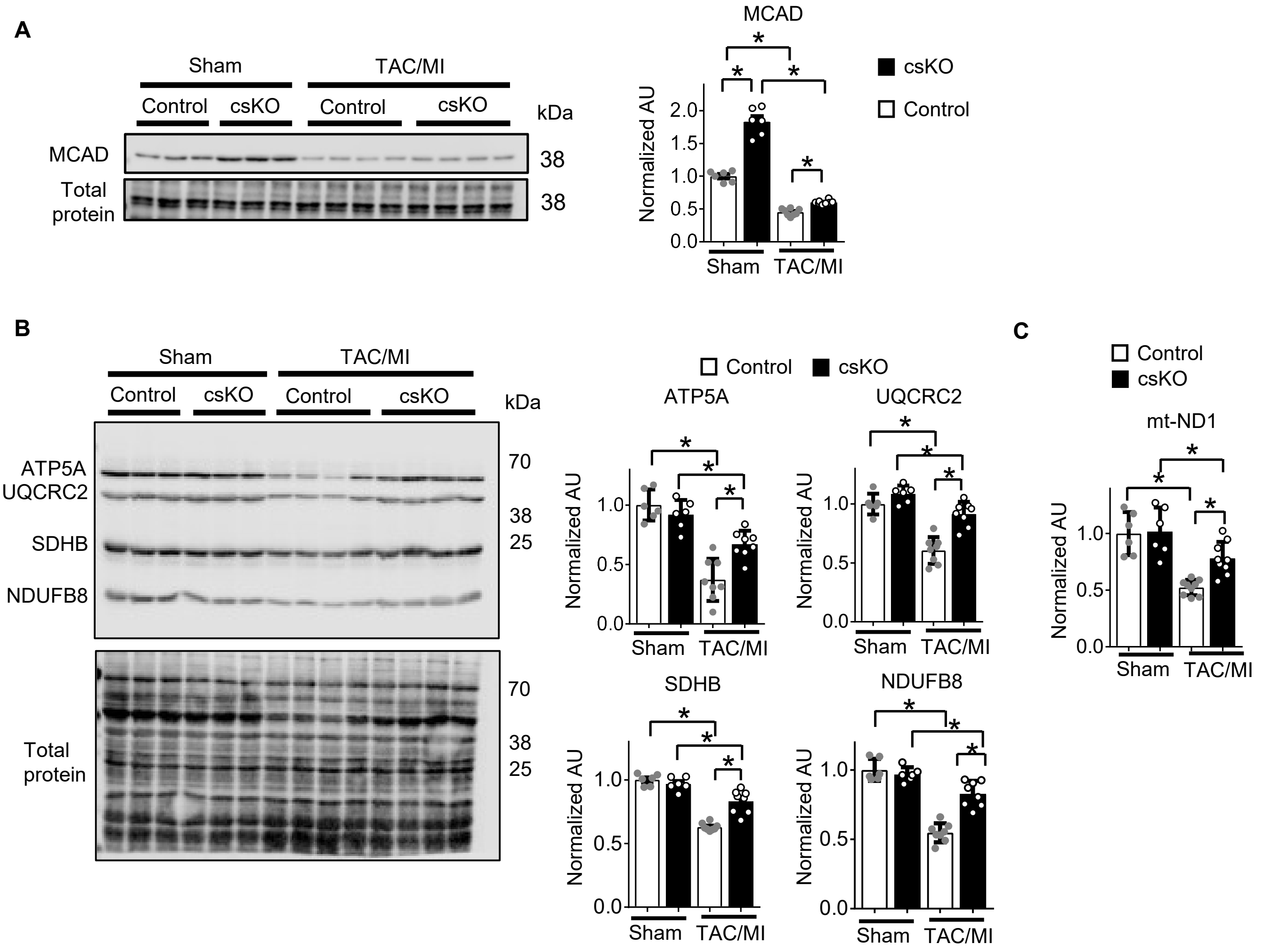

Supplemental Figure 9. Impact of RIP140 deficiency on representative target cardiac mitochondrial protein levels post-TAC/MI conditions. (A) Left; representative immunoblots directed at the fatty acid oxidation enzyme, MCAD, in csKO and Control biventricle lysates. Right; quantification of the immunoblots (n= 6-8 per group). (**B**) Left; representative immunoblots for Atp5a, Uqcrc2, Sdhb, and Ndufb8.Total protein is shown at the bottom. Right; quantification of immunoblots (n= 6-8 per group). Values are mean ± SEM. * $p < 0.05$ using 2-way ANOVA with Tukey's multiple comparison test. (**C**) Relative level of mitochondrial DNA encoding mt-Nd1 normalized to nuclear DNA in csKO and Control hearts (n= 6-9 per group).

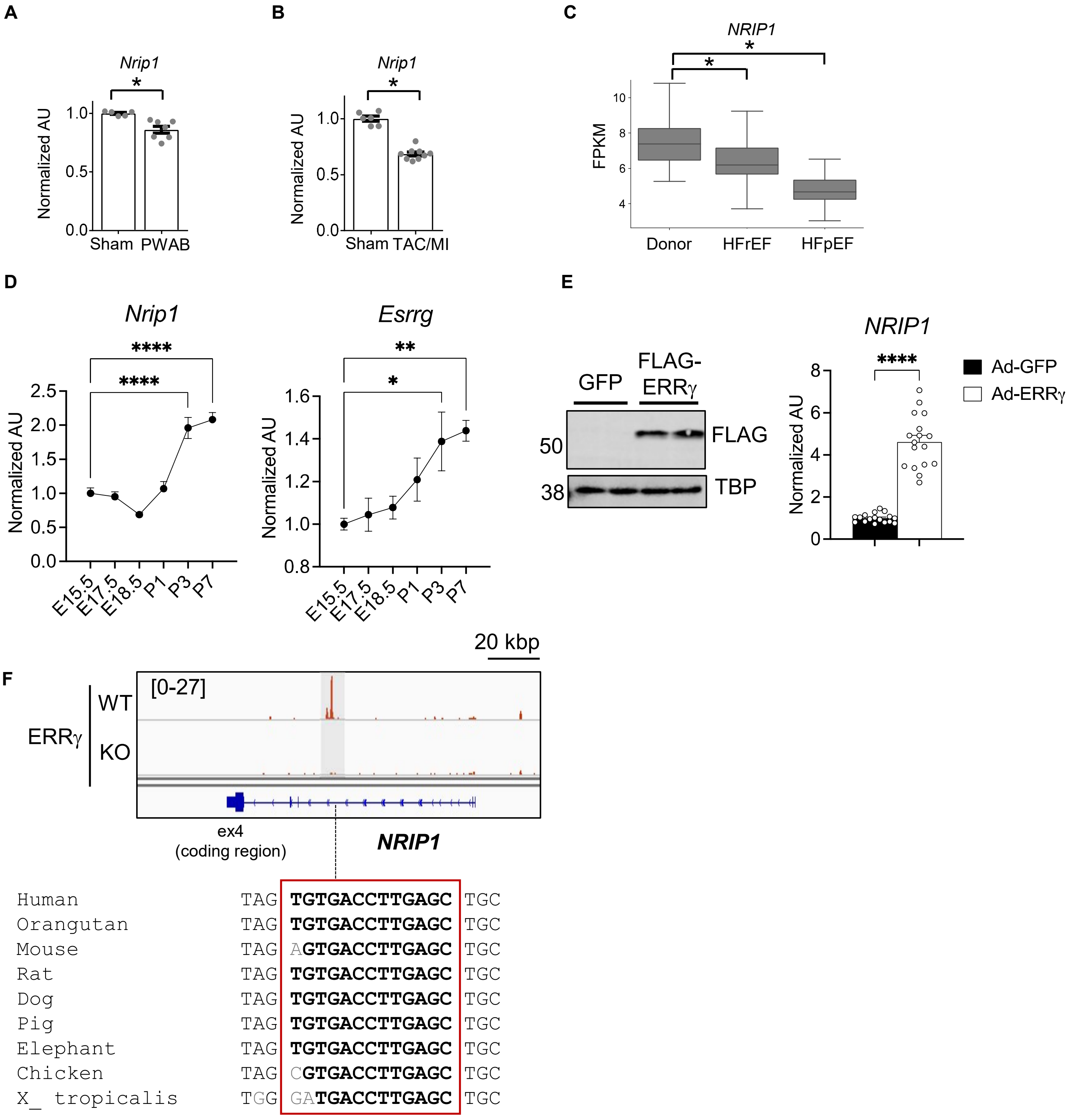

Supplemental Figure 10. Regulation of *Nrip1* expression in the failing and developing heart: A role for ERRγ feedback regulation. **(A),(B),** Real-time quantitative polymerase chain reaction (qRT-PCR) analysis of *Nrip1* expression in hearts of mice subjected to PWAB or TAC/MI surgeries (n=5-9 per group). **(C)** Box plots representing the fragments per kilobase of exon per million mapped fragments (FPKM) of *NRIP1* in each healthy donor control (n=24) and Heart Failure with reduced Ejection Fraction or HFrEF; (n=30), and Heart Failure with preserved Ejection Fraction or HFpEF (n=41) patient cohorts from published RNA-seq data (https://zenodo.org/record/4114617#.YWrnTNnMJ0w). * represents adjusted *p*-values (Benjamini-Hochberg false discovery rate) <0.05. **(D)** qRT-PCR-determined levels of *Nrip1* and *Esrrg* in mouse hearts from embryonic day (E)15.5 to postnatal day (P) 7. E15.5 and P1, n=4 and others, n=5. n denotes independent biological replicates. *$p<0.05$, **$p<0.01$, and ****$p<0.0001$ vs E15.5; one-way ANOVA followed by Dunnett's multiple comparisons test. **(E)** Left panel: representative immunoblot images of indicated protein in human induced pluripotent stem cell-derived cardiomyocytes (hiPSC-CMs) following the infection of adenovirus expressing GFP or FLAG-tagged ERRγ (Ad-GFP or Ad-ERRγ). TATA-binding protein (TBP) serves as nuclear protein loading control. Right panel: Bars represent *NRIP1* transcript level determined by RT-qPCR. ****$p<0.0001$ vs Ad-GFP, two-tailed student's t-test. n=17 for both groups obtained with at least three independent experiments. **(F)** Top, Genomic browser track of *NRIP1* locus in wild type (WT) and estrogen-related receptor γ (ERRγ) KO hiPSC-CMs. The chromatin immunoprecipitation sequencing (ChIP-seq) data with anti-ERRγ dataset was obtained from GSE113784. ERRγ peak signals are denoted in red. Lower panel shows the putative ERR binding site defined by JASPAR (https://jaspar.genereg.net/) and the marked conservation across species. Bold font indicates the ERR binding motif.

Supplemental Table 1. Pathways enriched in the RNA-seq from str*Nrip1*$^{-/-}$ cardiac ventricle.

Pathway/Function Categories	Genes	Gene Count	Fold Enrichment	Adjusted *p*-value
**GO_Biological Process-Upregulated in str*Nrip1*$^{-/-}$**				
2-oxoglutarate metabolic process	Idh3g, Ogdh, Got1, Idh2	4	28.98	0.015782
dendritic spine morphogenesis	Ephb3, Arc, Epha4, Eef2k, Sipa1l1, Dtnbp1	6	11.37	0.024883
dicarboxylic acid metabolic process	Idh3g, Ogdh, Shmt1, Got1, Idh2, Me3, Suclg2	7	11.2	0.005214
cellular respiration	Idh3g, Prkaca, Ogdh, Gpd1, Cox6a2, Coq10a, Slc25a23, Sdhc, Suclg2	9	7.44	0.006176
energy derivation by oxidation of organic compounds	Idh3g, Gnmt, Prkaca, Ogdh, Gpd1, Gnas, Cox6a2, Coq10a, Stk40, Slc25a23, Sdhc, Suclg2	12	6.48	0.000623
organic acid catabolic process	Etfb, Pipox, Shmt1, Asrgl1, Got1, Hadha, Echdc2, Fah, Bckdk	9	6.06	0.032572
carboxylic acid catabolic process	Etfb, Pipox, Shmt1, Asrgl1, Got1, Hadha, Echdc2, Fah, Bckdk	9	6.06	0.032572
generation of precursor metabolites and energy	Idh3g, Gnmt, Prkaca, Ogdh, Gpd1, Gnas, Cox6a2, Hk1, Coq10a, Stk40, Slc25a23, Sdhc, Suclg2, Eno1	14	4.97	0.00162
oxidation-reduction process	Idh3g, Gnmt, Etfb, Prkaca, Pipox, Ogdh, Gpd1, Hadha, Gnas, Echdc2, Me3, Cox6a2, Coq10a, Stk40, Slc25a23, Fto, Sdhc, Suclg2	18	4.49	0.000191
oxoacid metabolic process	Idh3g, Gnmt, As3mt, Etfb, Ptgds, Pipox, Pcbd1, Ogdh, Shmt1, Gpd1, Asrgl1, Got1, Hadha, Echdc2, Idh2, Me3, Fah, Bckdk, Slc27a1, Hk1, Prune2, Suclg2, Eno1	23	3.43	0.000382
carboxylic acid metabolic process	Idh3g, Gnmt, As3mt, Etfb, Ptgds, Pipox, Pcbd1, Ogdh, Shmt1, Gpd1, Asrgl1, Got1, Hadha, Echdc2, Idh2, Me3, Fah, Bckdk, Slc27a1, Hk1, Suclg2, Eno1	22	3.43	0.000752
organic acid metabolic process	Idh3g, Gnmt, As3mt, Etfb, Ptgds, Pipox, Pcbd1, Ogdh, Shmt1, Gpd1, Asrgl1, Got1, Hadha, Echdc2, Idh2, Me3, Fah, Bckdk, Slc27a1, Hk1, Prune2, Suclg2, Eno1	23	3.33	0.00066
small molecule metabolic process	Idh3g, Gnmt, As3mt, Etfb, Ptgds, Pipox, Pcbd1, Ogdh, Shmt1, Gpd1, Gnb3, Sik1, Asrgl1, Got1, Hadha, Echdc2, Idh2, Me3, Fah, Bckdk, Slc27a1, Hk1, Smpd1, Prune2, Coq10a, Hsd3b7, Suclg2, Eno1	28	2.36	0.027989
organic substance catabolic process	Psap, Etfb, Prkaca, Pttg1ip, Pipox, Shmt1, Ube2o, Clu, Dcaf11, Gpd1, Asrgl1, Got1, Hadha, Epha4, Pla2g12a, Echdc2, Fah, Bckdk, Herpud1, Oaz1, Hk1, Smpd1, Prune2, Wfs1, Csdc2, Pelo, Hsd3b7, Fto, Eno1, Rnaset2b	30	2.27	0.027518
regulation of cell differentiation	Hpn, Ppp1cc, Prkaca, Ephb3, Ppp2r1a, Ntn1, Gata4, Clu, Arc, Gnb3, Sik1, Epha4, Gnas, Slc46a2, Tesc, Idh2, Smarca4, Eef2k, Fstl4, Apbb1, Hand2, F11r, Dag1, Sipa1l1, Tenm4, Tob2, Fto, Cebpb, Dtnbp1, Itgb1bp1, Ccl21b	31	2.17	0.048989

Pathway/Function Categories	Genes	Gene Count	Fold Enrichment	Adjusted *p*-value
**GO_Cellular Component-Upregulated in str*Nrip1*$^{-/-}$**				
tricarboxylic acid cycle enzyme complex	Ogdh, Bckdk, Suclg2	3	29.44	0.036983
contractile fiber part	Svil, Myot, Abcc9, Myom3, Kcne1, Dag1, Pgm5, Actn2, 3425401B19Rik	9	5.76	0.00835
myofibril	Svil, Myot, Abcc9, Myom3, Kcne1, Dag1, Pgm5, Actn2, 3425401B19Rik	9	5.49	0.012142
dendritic spine	Ppp1cc, Prkaca, Arc, Epha4, Eef2k, Apbb1, Sipa1l1, Dtnbp1	8	5.45	0.034689
neuron spine	Ppp1cc, Prkaca, Arc, Epha4, Eef2k, Apbb1, Sipa1l1, Dtnbp1	8	5.23	0.045617
contractile fiber	Svil, Myot, Abcc9, Myom3, Kcne1, Dag1, Pgm5, Actn2, 3425401B19Rik	9	5.17	0.019384
mitochondrial membrane	Ppp1cc, Akap1, Ogdh, Clu, Hadha, Epha4, Mccc1, Abcb8, Idh2, Cox6a2, Slc27a1, Bdh1, Sdhc, Slc25a3, Cpt1b	15	3.52	0.00754
mitochondrial envelope	Ppp1cc, Akap1, Ogdh, Clu, Arl2, Hadha, Epha4, Mccc1, Abcb8, Idh2, Cox6a2, Slc27a1, Bdh1, Sdhc, Slc25a3, Cpt1b	16	3.4	0.006074
mitochondrial part	Ppp1cc, Etfb, Akap1, Ogdh, Clu, Mrpl14, Arl2, Hadha, Epha4, Mccc1, Abcb8, Idh2, Cox6a2, Bckdk, Slc27a1, Bdh1, Sdhc, Suclg2, Slc25a3, Cpt1b	20	3.22	0.001188
organelle envelope	Agpat3, Hpn, Emd, Ppp1cc, Akap1, Ogdh, Clu, Nr4a1, Igf2r, Arl2, Hadha, Epha4, Mccc1, Abcb8, Idh2, Cox6a2, Slc27a1, Osbpl6, Bdh1, Sdhc, Slc25a3, Cpt1b	22	2.79	0.003769
envelope	Agpat3, Hpn, Emd, Ppp1cc, Akap1, Ogdh, Clu, Nr4a1, Igf2r, Arl2, Hadha, Epha4, Mccc1, Abcb8, Idh2, Cox6a2, Slc27a1, Osbpl6, Bdh1, Sdhc, Slc25a3, Cpt1b	22	2.79	0.003832
whole membrane	Emd, Ppp1cc, Prkaca, Vac14, Akap1, Copz2, Arc, Epha4, Sec16a, Mal, Gnas, Map1lc3a, Sh3gl2, Slc27a1, Hk1, Wfs1, Dag1, Tuba1c, Syngr2, Dtnbp1, Eno1, Cpt1b, Entpd4	23	2.68	0.0044
mitochondrion	Idh3g, As3mt, Psap, Ppp1cc, Etfb, Prkaca, Akap1, Ogdh, Shmt1, Clu, Gpd1, Mrpl14, Arl2, Hadha, Epha4, Mccc1, Echdc2, Abcb8, Nipsnap2, Abcc9, Idh2, Me3, Cox6a2, Bckdk, Slc27a1, Hk1, Coq10a, Slc25a23, Bdh1, Gatd3a, Ech1, Sdhc, Suclg2, Slc25a3, Cpt1b, Tmem223	36	2.6	2.25E-05
organelle membrane	Agpat3, Hpn, Emd, Ppp1cc, Tmem147, Vac14, Akap1, Copz2, Ogdh, Clu, Nr4a1, Osbp, Hadha, Epha4, Sec16a, Gnas, Map1lc3a, Mccc1, Sh3gl2, Abcb8, Idh2, Cox6a2, Herpud1, Slc27a1, Wfs1, Osbpl6, Bdh1, Syngr2, Dtnbp1, Sdhc, Slc25a3, Rom1, Cpt1b, Entpd4, Strit1	35	2.2	0.001742
neuron part	Hpn, Ppp1cc, Prkaca, Eef1a2, Slc16a7, Clu, Arc, Gnb3, Myot, Asrgl1, Got1, Epha4, Gnas, Sh3gl2, Herpud1, Smarca4, Eef2k, Vezt, Apbb1, Wfs1, Dag1, Sipa1l1, Tenm4, Syngr2, Actn2, Fez2, Dtnbp1, Eno1, Rab3ip, Rom1	30	2.01	0.047082

Pathway/Function Categories	Genes	Gene Count	Fold Enrichment	Adjusted *p*-value
**KEGG_PATHWAY-Upregulated in str*Nrip1*$^{-/-}$**				
Citrate cycle (TCA cycle)	Idh3g, Ogdh, Idh2, Sdhc, Suclg2	5	14.5	0.001729
Carbon metabolism	Idh3g, Ogdh, Shmt1, Got1, Hadha, Idh2, Me3, Hk1, Sdhc, Suclg2, Eno1	11	8.51	4.49E-06

The table shows the significantly enriched ($p<0.05$, adjusted *p*-value by Benjamini-Hochberg FDR method) GOTERM_BP_DIRECT, GOTERM_CC_DIRECT and Kyoto Encyclopedia of Genes and Genomes (KEGG)_PATHWAY pathways defined by the gene sets of upregulated or downregulated genes in str*Nrip1*$^{-/-}$ RNA-seq. There are no significantly downregulated KEGG_PATHWAY in str*Nrip1*$^{-/-}$.

Supplemental Table 1. Continued

Pathway/Function Categories	Genes	Gene Count	Fold Enrichment	Adjusted *p*-value
**GO_Biological Process_Downregulated in str*Nrip1*$^{-/-}$**				
positive regulation of anion transmembrane transport	Acsl6, Arl6ip1, Ptafr	3	63.4	0.026071
cellular response to interferon-beta	Gbp2, Ifit1, Irgm2, 9930111J21Rik2, Ifit3	5	22.97	0.005408
response to interferon-beta	Gbp2, Ifit1, Irgm2, 9930111J21Rik2, Ifit3	5	18.22	0.017289
positive regulation of ion transmembrane transport	Acsl6, Hcn1, Arl6ip1, Stim1, Ctss, Ptafr, Dpp6	7	8.6	0.037651
cellular response to cytokine stimulus	Rarg, Cd74, Ptprc, Lrat, Gbp2, Cntfr, Parp14, Ifit1, Gbp7, Npnt, Irgm2, 9930111J21Rik2, Ifit3	13	4.02	0.040748
response to cytokine	Rarg, Cd74, Ptprc, Lrat, Gbp2, Cntfr, Parp14, Ifit1, H2-Aa, Gbp7, Npnt, Irgm2, 9930111J21Rik2, Ifit3	14	3.83	0.032349
defense response to other organism	Slamf6, Cybb, Rsad2, Cd74, Ptprc, Itch, Gbp2, Parp14, Ifit1, H2-Aa, Gbp7, Lyz2, Slfn9, Irgm2, Ifit3	15	3.62	0.030093
response to other organism	Slamf6, Cybb, Rsad2, Cd74, Ptprc, Car3, Itch, Lrat, Gbp2, Parp14, Ifit1, H2-Aa, Gbp7, Ptafr, Cfd, Lyz2, Slfn9, Irgm2, Hba-a2, Ifit3	20	3.56	0.001123
response to external biotic stimulus	Slamf6, Cybb, Rsad2, Cd74, Ptprc, Car3, Itch, Lrat, Gbp2, Parp14, Ifit1, H2-Aa, Gbp7, Ptafr, Cfd, Lyz2, Slfn9, Irgm2, Hba-a2, Ifit3	20	3.55	0.001168
response to biotic stimulus	Slamf6, Cybb, Rsad2, Cd74, Ptprc, Car3, Itch, Lrat, Gbp2, Parp14, Ifit1, H2-Aa, Gbp7, Ptafr, Cfd, Lyz2, Slfn9, Irgm2, Hba-a2, Ifit3	20	3.49	0.001499
defense response	Slamf6, Cybb, Rsad2, Pbk, Cd74, Ptprc, Itch, Gbp2, Parp14, Ifit1, H2-Aa, Ctss, Gbp7, Ptafr, Cyp26b1, Lyz2, Slfn9, Irgm2, 9930111J21Rik2, Ifit3	20	3.16	0.00702
response to external stimulus	Slamf6, Cybb, Rsad2, Pbk, Cd74, Ptprc, Car3, Itch, Postn, Lrat, Gbp2, Dapk2, Parp14, Ifit1, H2-Aa, Ctss, Itga9, Camk1d, Gbp7, Npnt, Ptafr, Ryr3, Cfd, Lyz2, Slfn9, Irgm2, Hba-a2, Ifit3	28	2.54	0.003875

The table shows the significantly enriched ($p<0.05$, adjusted *p*-value by Benjamini-Hochberg FDR method) GOTERM_BP_DIRECT, GOTERM_CC_DIRECT and Kyoto Encyclopedia of Genes and Genomes (KEGG)_PATHWAY pathways defined by the gene sets of upregulated or downregulated genes in str*Nrip1*$^{-/-}$ RNA-seq. There are no significantly downregulated KEGG_PATHWAY in str*Nrip1*$^{-/-}$.

Supplemental Table 2. Physiological parameters and echocardiographic analysis in control vs str*Nrip1*$^{-/-}$ mice.

	Control	str*Nrip1*$^{-/-}$
Body weight (g)	19.7 ± 1.1	21.5 ± 1.7
Tibia length (mm)	16.0 ± 0.2	16.1 ± 0.2
Biventricular weight (BV) (mg)	87.3 ± 4.4	113.3 ± 9.4*
BV / Tibia length (mg/mm)	5.43 ± 0.21	7.02 ± 0.56*
Heart rate (bpm)	602.0 ± 11.4	625.5 ± 8.9
LVPWd (mm)	0.752 ± 0.015	0.844 ± 0.024*
IVSd (mm)	0.752 ± 0.015	0.859 ± 0.030*
LVIDd (mm)	3.257 ± 0.076	3.318 ± 0.110
LVPWs (mm)	1.101 ± 0.047	1.196 ± 0.028
IVSs (mm)	1.178 ± 0.044	1.222 ± 0.044
LVIDs (mm)	2.004 ± 0.028	2.077 ± 0.030
LVM (mg)	76.5 ± 3.2	94.3 ± 7.0*
LVMI (mg/g Body weight)	3.70 ± 0.17	4.50 ± 0.30*
RWT	0.46 ± 0.02	0.52 ± 0.02
FS (%)	38.38 ± 0.99	38.10 ± 1.07

Echocardiography was performed on 8 week-old male mice (n=6 per each group). Values are mean ± SEM. *$p < 0.05$ vs control using two-tailed, unpaired t-test. LVPWd, left ventricular posterior wall thickness at end-diastole; IVSd, interventricular septum thickness at end-diastole; LVIDd, left ventricular internal dimension at end-diastole; LVPWs, left ventricular posterior wall thickness at end-systole; IVSs, interventricular septum thickness at end-systole; LVIDs, left ventricular internal dimension at end-systole; LVM, left ventricular mass; LVMI, left ventricular mass index; RWT, relative wall thickness; FS, fractional shortening

Supplemental Table 3. Pathways enriched in the RNA-seq from str*Nrip1*$^{-/-}$ gastrocnemius.

Pathway/Function Categories	Genes	Gene Count	Fold Enrichment	Adjusted *p*-value
**GO_Biological Process-Upregulated in strNrip1$^{-/-}$**				
electron transport coupled proton transport	Ndufs7, mt-Co1, mt-Nd4, mt-Cytb	4	21.21	0.010234
fatty acid beta-oxidation using acyl-CoA dehydrogenase	Gcdh, Etfb, Acadvl, Ivd, Etfdh, Acads, Etfa, Acadm	8	18.85	4.26E-07
succinyl-CoA metabolic process	Dlst, Ogdh, Sucla2, Acot4, Suclg1, Suclg2	6	18.18	0.000151
branched-chain amino acid catabolic process	Aldh6a1, Mccc2, Ivd, Hmgcl, Hibadh, Bckdk, Bcat2, Acat1, Bckdhb	9	17.35	1.17E-07
glutamate catabolic process	Glud1, Got1, Adhfe1, Got2	4	16.97	0.049249
dicarboxylic acid catabolic process	Acsf3, Glud1, Got1, Adhfe1, Got2, Acot4, Ddo	7	14.84	0.000112
tricarboxylic acid cycle	Sdhd, Idh3g, Cs, Mdh2, Mdh1, Ogdh, Pdhb, Ogdhl, Sucla2, Aco2, Fh1, Idh3b, Idh3a, Suclg1, Suclg2	15	13.83	8.09E-12
ATP synthesis coupled proton transport	Atp5d, Atp5g1, Atp5g3, Cyc1, Atp5o, Atp5b, Atp5a1, Atp5c1, Atp5h, Atp5l, mt-Atp6	11	12.28	2.68E-07
short-chain fatty acid metabolic process	Phyh, Acss1, Pck1, Acss2, Acads, Acot4	6	10.6	0.016292
acetyl-CoA biosynthetic process from pyruvate	Dlat, Pdhx, Dld, Pdhb, Mpc2, Pdk2	6	10.6	0.016292
ribonucleoside bisphosphate biosynthetic process	Dlat, Coasy, Gcdh, Pdhx, Acsl1, Dld, Pdhb, Mpc2, Acss1, Acss2, Acot7, Pank4, Acat1, Pank1, Pdk2, Acacb, Mlycd	17	10.3	1.03E-10
ATP synthesis coupled electron transport	Cox5a, Sdhd, Ndufs2, Cox7c, Dld, Sdha, Cyc1, Ndufv2, Uqcrc1, Ndufa10, Pink1, Cox6a2, Coq9, Cox4i1, Ndufv1, Uqcrq, Bdnf, Gm10053, Uqcr10, Ndufs8, mt-Co1, mt-Co2, mt-Co3, Ndufb6	24	9.98	1.53E-15
mitochondrial ATP synthesis coupled electron transport	Cox5a, Sdhd, Ndufs2, Cox7c, Dld, Sdha, Cyc1, Ndufv2, Uqcrc1, Ndufa10, Pink1, Cox6a2, Coq9, Cox4i1, Ndufv1, Uqcrq, Bdnf, Gm10053, Uqcr10, Ndufs8, mt-Co1, mt-Co3, Ndufb6	23	9.95	8.85E-15
negative regulation of fatty acid oxidation	Plin5, Acadvl, Appl2, Sirt4, Dgat2, Acacb	6	9.79	0.029047
respiratory electron transport chain	Cox5a, Sdhd, Sod2, Sdhb, Ndufs2, Slc25a13, Cox7c, Dld, Sdha, Cyc1, Ndufa5, Ndufv2, Uqcrc1, Ndufa10, Slc25a12, Etfdh, Pink1, Cox6a2, Coq9, Cox4i1, Ndufv1, Mybbp1a, Uqcrq, Bdnf, Gm10053, Uqcr10, Ndufs8, mt-Co1, mt-Co2, mt-Co3, Ndufb6	31	9.53	6.77E-20
fatty acid beta-oxidation	Gcdh, Etfb, Pex5, Plin5, Acadvl, Acox1, Eci2, Eci1, Hsd17b4, Echs1, Hadha, Ivd, Etfdh, Hadh, Abcd3, Decr1, Echdc2, Cpt2, Acads, Acat1, Etfa, Acaa2, Acacb, Hadhb, Acadm, Mlycd	26	9.19	7.14E-16
aerobic respiration	Cox5a, Sdhd, Oxa1l, Idh3g, Atp5d, Cs, Sdhb, Cox7c, Mdh2, Ndufs7, Mdh1, Ogdh, Pdhb, Ogdhl, Sucla2, Aco2, Shmt2, Fh1, Cat, Idh3b, Cox6a2, Cox4i1, Idh3a, Suclg1, Gm10053, Uqcr10, Ndufs8, Suclg2, mt-Nd1, mt-Co1, mt-Co3, mt-Nd4	32	9.17	5.79E-20
ubiquinone biosynthetic process	Ndufa9, Coq6, Coq8a, Coq9, Coq10a, Coq5	6	9.09	0.048804
regulation of cardiac muscle contraction by calcium ion signaling	Ryr2, Casq2, Ank2, Tnni3, Pln, Cacna1c	6	9.09	0.048804
mitochondrial respiratory chain complex I assembly	Ndufa9, Oxa1l, Ndufb2, Ndufa1, Ndufs7, Ndufb9, Ndufa5, Timm21, Ndufb8, Ndufa10, Ndufa8, Ndufab1, Ndufa3, Aifm1, Foxred1, Ndufb10, Ndufs8, Ndufb6	18	8.88	4.66E-10
dicarboxylic acid metabolic process	Idh3g, Dlst, Cs, Sdhb, Acsf3, Mdh2, Mdh1, Ogdh, Dld, Dglucy, Sdha, Glud1, Sucla2, Got1, Shmt2, Adhfe1, Fh1, Phyh, Idh3b, Pck1, Idh2, Me3, Oat, Got2, Idh3a, Aldh5a1, Kyat3, Acot4, Suclg1, Suclg2, Ddo, Ass1	32	8.81	2.61E-19
acyl-CoA metabolic process	Dlat, Gcdh, Dlst, Cs, Pdhx, Acsl1, Ogdh, Dld, Acot2, Pdhb, Sucla2, Hsd17b4, Mpc2, Acss1, Acss2, Hmgcs2, Pmvk, Hmgcl, Acot7, Dgat2, Acadsb, Acsm5, Nudt7, Acat1, Acot11, Acaa2, Pdk2, Acacb, Acot4, Suclg1, Suclg2, Acot1, Mlycd	33	8.53	1.93E-19
ribonucleoside bisphosphate metabolic process	Dlat, Coasy, Gcdh, Dlst, Cs, Pdhx, Acsl1, Ogdh, Dld, Acot2, Mccc2, Pdhb, Sucla2, Hsd17b4, Mpc2, Acss1, Acss2, Hmgcs2, Pmvk, Hmgcl, Acot7, Pank4, Dgat2, Acadsb, Acsm5, Nudt7, Acat1, Pank1, Acot11, Acaa2, Pdk2, Acacb, Acot4, Suclg1, Suclg2, Acot1, Mlycd	37	8.26	1.95E-21
purine nucleoside bisphosphate metabolic process	Dlat, Coasy, Gcdh, Dlst, Cs, Pdhx, Acsl1, Ogdh, Dld, Acot2, Mccc2, Pdhb, Sucla2, Hsd17b4, Mpc2, Acss1, Acss2, Hmgcs2, Pmvk, Hmgcl, Acot7, Pank4, Dgat2, Acadsb, Acsm5, Nudt7, Acat1, Pank1, Acot11, Acaa2, Pdk2, Acacb, Acot4, Suclg1, Suclg2, Acot1, Mlycd	37	8.26	1.95E-21
fatty acid catabolic process	Lipe, Gcdh, Etfb, Pex5, Plin5, Acadvl, Acox1, Acot2, Eci2, Eci1, Hsd17b4, Echs1, Hadha, Phyh, Ivd, Pck1, Etfdh, Hadh, Abcd3, Decr1, Echdc2, Cpt2, Acot7, Acads, Acat1, Etfa, Acaa2, Acacb, Hadhb, Acadm, Mlycd	31	8.22	1.48E-17
monocarboxylic acid catabolic process	Lipe, Gcdh, Etfb, Pex5, Plin5, Acadvl, Acox1, Acot2, Eci2, Eci1, Hsd17b4, Echs1, Hadha, Phyh, Ivd, Pck1, Etfdh, Hadh, Abcd3, Decr1, Echdc2, Cpt2, Acot7, Acads, Fah, Ldhd, Acat1, Etfa, Aldh5a1, Acaa2, Acacb, Idnk, Abat, Hadhb, Acadm, Mlycd	36	8.12	1.67E-20
oxidative phosphorylation	Cox5a, Sdhd, Ndufs2, Cox7c, Dld, Sdha, Ppif, Cyc1, Atp5o, Ndufv2, Atp5b, Shmt2, Uqcrc1, Ndufa10, Ak4, Pink1, Cox6a2, Coq9, Cox4i1, Ndufv1, Uqcrq, Bdnf, Chchd10, Gm10053, Uqcr10, Ndufs8, mt-Co1, mt-Co2, mt-Co3, Ndufb6, Cox7a1	31	7.92	5.38E-17
cellular respiration	Cox5a, Sdhd, Oxa1l, Idh3g, Atp5d, Cs, Trap1, Sod2, Sdhb, Ndufs2, Slc25a13, Cox7c, Mdh2, Ndufs7, Mdh1, Ogdh, Dld, Sdha, Pdhb, Ogdhl, Sucla2, Aco2, Cyc1, Ndufa5, Ndufv2, Shmt2, Uqcrc1, Ndufs1, Ndufa10, Fh1, Slc25a12, Cat, Idh3b, Etfdh, Pink1, Cox6a2, Coq9, Cox4i1, Idh3a, Ndufv1, Coq10a, Mybbp1a, Uqcrq, Bdnf, Suclg1, Gm10053, Uqcr10, Ndufs8, Suclg2, mt-Nd1, mt-Co1, mt-Co2, mt-Co3, mt-Nd4, Ndufb6	55	7.83	1.85E-31
purine nucleoside triphosphate biosynthetic process	Atp5d, Stat3, Atp5g1, Slc25a13, Atp5g3, Cyc1, Atp5o, Atp5b, Atp5a1, Atp5c1, Ak4, Ldhd, Atp5h, Atp5l, Adk, Impdh2, mt-Co2, mt-Atp6	18	7.79	6.76E-09
ATP biosynthetic process	Atp5d, Stat3, Atp5g1, Slc25a13, Atp5g3, Cyc1, Atp5o, Atp5b, Atp5a1, Atp5c1, Ak4, Ldhd, Atp5h, Atp5l, mt-Co2, mt-Atp6	16	7.54	1.94E-07
purine ribonucleoside triphosphate biosynthetic process	Atp5d, Stat3, Atp5g1, Slc25a13, Atp5g3, Cyc1, Atp5o, Atp5b, Atp5a1, Atp5c1, Ak4, Ldhd, Atp5h, Atp5l, Impdh2, mt-Co2, mt-Atp6	17	7.51	5.39E-08
lipid oxidation	Cd36, Gcdh, Etfb, Pex5, Plin5, Acadvl, Appl2, Acox1, Eci2, Apod, Eci1, Hsd17b4, Echs1, Hadha, Phyh, Ivd, Etfdh, Hadh, Abcd3, Decr1, Echdc2, Cpt2, Fabp3, Sirt4, Acads, Dgat2, Acat1, Etfa, Acaa2, Acacb, Hadhb, Acadm, Mlycd	33	7.44	3.08E-17
sulfur compound catabolic process	Ggt5, Csad, Ahcy, Acot7, Nudt7, Acat1, Mlycd	7	7.42	0.047628
fatty acid oxidation	Cd36, Gcdh, Etfb, Pex5, Plin5, Acadvl, Appl2, Acox1, Eci2, Eci1, Hsd17b4, Echs1, Hadha, Phyh, Ivd, Etfdh, Hadh, Abcd3, Decr1, Echdc2, Cpt2, Fabp3, Sirt4, Acads, Dgat2, Acat1, Etfa, Acaa2, Acacb, Hadhb, Acadm, Mlycd	32	7.38	1.63E-16
regulation of mitochondrial translation	Uqcc1, Lrpprc, Shmt2, Nsun4, Rpusd4, Mtg1, Trub2, Mrps27, Rcc1l	9	7.34	0.003486
NADH metabolic process	Idh3g, Dlst, Mdh2, Mdh1, Ogdh, Nudt13, Idh3b, Aldob, Idh3a, Foxk1	10	7.31	0.000951
ribonucleoside triphosphate biosynthetic process	Atp5d, Stat3, Atp5g1, Slc25a13, Atp5g3, Cyc1, Atp5o, Atp5b, Atp5a1, Atp5c1, Ak4, Ldhd, Atp5h, Atp5l, Impdh2, mt-Co2, mt-Atp6	17	7.07	1.64E-07
regulation of mitochondrial gene expression	Uqcc1, Lrpprc, Shmt2, Nsun4, Rpusd4, Mtg1, Trub2, Mrps27, Chchd10, Rcc1l	10	7.07	0.001366
cell communication involved in cardiac conduction	Jup, Ryr2, Dsc2, Kcnj5, Scn5a, Ank2, Rangrf, Tnni3k, Dsg2, Scn4b, Cacna1c, Dsp	12	6.88	0.000145
purine ribonucleotide biosynthetic process	Dlat, Coasy, Atp5d, Gcdh, Stat3, Atp5g1, Aprt, Pdhx, Slc25a13, Atp5g3, Acsl1, Dld, Pdhb, Cyc1, Atp5o, Atp5b, Atp5a1, Atp5c1, Mpc2, Acss1, Acss2, Npr1, Npr2, Ak4, Acot7, Pank4, Ldhd, Acat1, Pank1, Atp5h, Atp5l, Pdk2, Nppa, Acacb, Impdh2, mt-Co2, mt-Atp6, Mlycd	38	6.83	1.28E-18
nucleoside triphosphate biosynthetic process	Atp5d, Stat3, Atp5g1, Slc25a13, Atp5g3, Cyc1, Atp5o, Atp5b, Atp5a1, Atp5c1, Ak4, Ldhd, Atp5h, Atp5l, Adk, Suclg1, Impdh2, mt-Co2, mt-Atp6	19	6.83	2.52E-08
ribonucleotide biosynthetic process	Dlat, Coasy, Atp5d, Gcdh, Stat3, Atp5g1, Aprt, Pdhx, Slc25a13, Atp5g3, Acsl1, Dld, Pdhb, Cyc1, Atp5o, Atp5b, Atp5a1, Atp5c1, Mpc2, Acss1, Acss2, Npr1, Npr2, Ak4, Acot7, Pank4, Ldhd, Acat1, Pank1, Atp5h, Atp5l, Pdk2, Nppa, Acacb, Impdh2, mt-Co2, mt-Atp6, Mlycd	38	6.45	1.27E-17
proton transmembrane transport	Atp5d, Phb2, Clcn3, Atp5g1, Atp1a2, Atp5g3, Ndufs7, Ppif, Cyc1, Atp5o, Atp5b, Atp5a1, Atp5c1, Tesc, Slc25a4, Atp5h, Atp5l, Chchd10, mt-Co1, mt-Atp6, mt-Nd4, mt-Cytb	22	6.39	2.75E-09
organic acid catabolic process	Lipe, Gcdh, Etfb, Pex5, Plin5, Acsf3, Acadvl, Acox1, Acot2, Aldh6a1, Eci2, Mccc2, Glud1, Csad, Eci1, Hsd17b4, Got1, Shmt2, Echs1, Hadha, Adhfe1, Phyh, Ivd, Pck1, Ahcy, Etfdh, Hadh, Abcd3, Decr1, Echdc2, Cpt2, Hmgcl, Acot7, Acads, Hibadh, Fah, Bckdk, Bcat2, Oat, Got2, Ldhd, Acat1, Bckdhb, Etfa, Aldh5a1, Amdhd2, Acaa2, Acacb, Idnk, Acot4, Abat, Hadhb, Acadm, Ddo, Mlycd	55	6.37	3.63E-26

Supplemental Table 3. Continued

Pathway/Function Categories	Genes	Gene Count	Fold Enrichment	Adjusted *p*-value
**GO_Biological Process-Upregulated in str*Nrip1*$^{-/-}$**				
regulation of heart rate by cardiac conduction	Jup, Dsc2, Kcnj5, Scn5a, Ank2, Dsg2, Scn4b, Cacna1c, Dsp	9	6.36	0.013464
regulation of cardiac muscle cell contraction	Jup, Atp1a2, Ryr2, Gata4, Dsc2, Casq2, Scn5a, Ank2, Rangrf, Pln, Dsg2, Cacna1c, Dsp	13	6.27	0.000145
purine ribonucleoside triphosphate metabolic process	Atp5d, Stat3, Atp5g1, Slc25a13, Atp5g3, Cyc1, Atp5o, Atp5b, Atp5a1, Atp5c1, Mfn1, Ak4, Ldhd, Atp5h, Atp5l, Impdh2, mt-Co2, mt-Atp6	18	6.26	4.5E-07
mitochondrial respiratory chain complex assembly	Ndufa9, Oxa1l, Ndufb2, Tfam, Uqcc1, Ndufa1, Ndufs7, Ndufb9, Samm50, Ndufa5, Timm21, Ndufb8, Ndufa10, Ndufa8, Ndufab1, Ndufa3, Aifm1, Foxred1, Ndufb10, Uqcr10, Ndufs8, Ndufb6	22	6.22	5.06E-09
purine nucleotide biosynthetic process	Dlat, Coasy, Atp5d, Gcdh, Stat3, Atp5g1, Aprt, Pdhx, Slc25a13, Atp5g3, Acsl1, Dld, Pdhb, Cyc1, Atp5o, Gart, Atp5b, Atp5a1, Atp5c1, Mpc2, Acss1, Acss2, Npr1, Npr2, Ak4, Acot7, Pank4, Ldhd, Acat1, Pank1, Atp5h, Atp5l, Pdk2, Adk, Nppa, Acacb, Impdh2, mt-Co2, mt-Atp6, Mlycd	40	6.15	8.37E-18
coenzyme biosynthetic process	Dlat, Ndufa9, Coasy, Gcdh, Pdhx, Acsl1, Nampt, Dld, Coq6, Pdhb, Gart, Coq8a, Mpc2, Acss1, Acss2, Acot7, Pank4, Idh2, Coq9, Acat1, Pank1, Pdk2, Coq10a, Coq5, Acacb, Mlycd	26	6.13	7.67E-11
energy derivation by oxidation of organic compounds	Cox5a, Sdhd, Oxa1l, Idh3g, Gnmt, Atp5d, Gys1, Insr, Cs, Trap1, Inpp5k, Sod2, Sdhb, Ndufs2, Slc25a13, Cox7c, Mdh2, Ndufs7, Mdh1, Ogdh, Dld, Sdha, Pdhb, Ogdhl, Sucla2, Aco2, Cyc1, Gbe1, Gsk3b, Ndufa5, Ndufv2, Shmt2, Uqcrc1, Rb1cc1, Ndufs1, Ndufa10, Fh1, Slc25a12, Cat, Idh3b, Etfdh, Pink1, Cox6a2, Coq9, Cox4i1, Idh3a, Pygb, Ndufv1, Coq10a, Mybbp1a, Uqcrq, Bdnf, Suclg1, Adgrf5, Gm10053, Uqcr10, Ndufs8, Suclg2, Acadm, mt-Nd1, mt-Co1, mt-Co2, mt-Co3, mt-Nd4, Ndufb6	65	6.05	8.08E-30
ribonucleoside triphosphate metabolic process	Atp5d, Stat3, Atp5g1, Slc25a13, Atp5g3, Cyc1, Atp5o, Atp5b, Atp5a1, Atp5c1, Mfn1, Ak4, Ldhd, Atp5h, Atp5l, Impdh2, mt-Co2, mt-Atp6	18	5.87	1.43E-06
regulation of actin filament-based movement	Jup, Atp1a2, Ryr2, Gata4, Dsc2, Tnnt2, Casq2, Scn5a, Ank2, Rangrf, Pln, Dsg2, Cacna1c, Dsp	14	5.71	0.000167
cardiac conduction	Jup, Nkx2-5, Ryr2, Dsc2, Kcnj5, Scn5a, Ank2, Rangrf, Pln, Tnni3k, Dsg2, Scn4b, Cacna1c, Dsp, Tmem65	15	5.68	6.22E-05
ATP metabolic process	Cox5a, Sdhd, Atp5d, Stat3, Insr, Atp5g1, Slc4a1, Atp1a2, Ndufs2, Slc25a13, Clpx, Cox7c, Atp5g3, Ddit4, Dld, Sdha, Ppif, Cyc1, Zbtb20, Atp5j, Atp5o, Ndufv2, Atp5b, Shmt2, Atp5a1, Uqcrc1, Atp5c1, Ndufs1, Ndufa10, Aldob, Ak4, Pink1, Cox6a2, Coq9, Cox4i1, Ldhd, Mpi, Mif, Atp5h, Hk1, Ndufv1, Atp5j2, Atp5l, Myh6, Uqcrq, Bdnf, Chchd10, Foxk1, Gm10053, Uqcr10, Ndufs8, mt-Co1, mt-Co2, mt-Atp6, mt-Co3, Fbp1, Ndufb6, Cox7a1	58	5.67	1.02E-24
coenzyme metabolic process	Dlat, Ndufa9, Coasy, Gnmt, Gcdh, Dlst, Cs, Pdhx, Acsl1, Ogdh, Nampt, Dld, Acot2, Coq6, Mccc2, Pdhb, Nudt13, Sucla2, Gart, Hsd17b4, Shmt2, Coq8a, Mpc2, Acss1, Ahcy, Acss2, Hmgcs2, Pmvk, Hmgcl, Acot7, Pank4, Idh2, Dgat2, Acadsb, Acsm5, Nudt7, Coq9, Acat1, Pank1, Acot11, Acaa2, Pdk2, Coq10a, Coq5, Acacb, Acot4, Suclg1, Suclg2, Mocs1, Acot1, Mlycd	51	5.66	1.91E-21
cellular amino acid catabolic process	Aldh6a1, Mccc2, Glud1, Csad, Got1, Shmt2, Adhfe1, Ivd, Ahcy, Hmgcl, Hibadh, Fah, Bckdk, Bcat2, Oat, Got2, Acat1, Bckdhb, Aldh5a1, Abat, Ddo	21	5.64	1.27E-07
generation of precursor metabolites and energy	Cox5a, Sdhd, Oxa1l, Idh3g, Gnmt, Atp5d, Gys1, Stat3, Phb2, Insr, Cs, Trap1, Inpp5k, Slc4a1, Sod2, Sdhb, Ndufs2, Slc25a13, Cox7c, Mdh2, Ddit4, Ndufs7, Mdh1, Ogdh, Dld, Acox1, Sdha, Pdhb, Ppif, Ogdhl, Sucla2, Oxct1, Aco2, Cyc1, Gbe1, Zbtb20, Gsk3b, Atp5o, Ndufa5, Ndufv2, Atp5b, Shmt2, Uqcrc1, Rb1cc1, Ndufs1, Ndufa10, Fh1, Slc25a12, Cat, Idh3b, Etfdh, Aldob, Ak4, Hmgcl, Pink1, Cox6a2, Coq9, Cox4i1, Acat1, Idh3a, Mpi, Pygb, Mif, Hk1, Ndufv1, Coq10a, Mybbp1a, Uqcrq, Bdnf, Chchd10, Suclg1, Adgrf5, Foxk1, Gm10053, Uqcr10, Ndufs8, Suclg2, Acadm, mt-Nd1, mt-Co1, mt-Co2, mt-Co3, mt-Nd4, Fbp1, Ndufb6, Adh1, Cox7a1	87	5.32	5.56E-36
alpha-amino acid catabolic process	Aldh6a1, Mccc2, Glud1, Csad, Got1, Shmt2, Adhfe1, Ivd, Ahcy, Hmgcl, Hibadh, Fah, Bcat2, Oat, Got2, Acat1, Ddo	17	5.08	4.72E-05
negative regulation of striated muscle cell apoptotic process	Nkx2-5, Gata4, Cflar, Sirt4, Slc25a4, Hand2, Rgl2, Sirt5, Naca, Acot1	10	4.93	0.049891
nucleoside triphosphate metabolic process	Atp5d, Stat3, Atp5g1, Slc25a13, Atp5g3, Cyc1, Atp5o, Atp5b, Atp5a1, Atp5c1, Mfn1, Ak4, Ldhd, Atp5h, Atp5l, Adk, Suclg1, Impdh2, mt-Co2, mt-Atp6	20	4.88	5.89E-06
small molecule catabolic process	Lipe, Gcdh, Etfb, Pex5, Inpp5k, Plin5, Acsf3, Acadvl, Acox1, Acot2, Aldh6a1, Eci2, Mccc2, Glud1, Oxct1, Csad, Eci1, Hsd17b4, Got1, Shmt2, Echs1, Hadha, Adhfe1, Phyh, Ivd, Pck1, Ahcy, Etfdh, Hadh, Abcd3, Decr1, Aldob, Echdc2, Cpt2, Hmgcl, Acot7, Acads, Hibadh, Fah, Bckdk, Bcat2, Oat, Got2, Ldhd, Acat1, Bckdhb, Mpi, Etfa, Aldh5a1, Amdhd2, Acaa2, Inpp4b, Acacb, Idnk, Acot4, Foxk1, Abat, Hadhb, Acadm, Ddo, Mlycd, Adh1	62	4.87	1.24E-22
cofactor biosynthetic process	Dlat, Ndufa9, Coasy, Gcdh, Stat3, Sod2, Pdhx, Acsl1, Nampt, Dld, Coq6, Pdhb, Gart, Hspa9, Coq8a, Mpc2, Acss1, Acss2, Gss, Acot7, Pank4, Idh2, Coq9, Acat1, Pank1, Pdk2, Coq10a, Coq5, Acacb, Isca1, Iba57, Ppox, Mocs1, mt-Co2, Mlycd	35	4.82	1.01E-11
myofibril assembly	Myl2, Nkx2-5, Cflar, Tnnt2, Casq2, Foxp1, Csrp3, Mylk3, Myom3, Myh6, Pgm5, Actn2, Nebl, Akap13	14	4.71	0.002182
cofactor metabolic process	Dlat, Ndufa9, Coasy, Idh3g, Gnmt, Gcdh, Gstm7, Stat3, Dlst, Insr, Cs, Ggt5, Sod2, Mgst1, Pdhx, Acsl1, Ogdh, Nampt, Dld, Acot2, Coq6, Mccc2, Pdhb, Nudt13, Sucla2, Oplah, Gart, Hspa9, Hsd17b4, Prdx5, Got1, Shmt2, Adhfe1, Gsta3, Coq8a, Mpc2, Phyh, Cat, Idh3b, Acss1, Ahcy, Acss2, Gss, Hmgcs2, Pmvk, Hmgcl, Pink1, Acot7, Pank4, Gstk1, Idh2, Dgat2, Acadsb, Acsm5, Gsr, Got2, Nudt7, Coq9, Acat1, Idh3a, Pank1, Acot11, Acaa2, Pdk2, Coq10a, Kyat3, Coq5, Acacb, Isca1, Iba57, Acot4, Suclg1, Gm10053, Suclg2, Ppox, Mocs1, Ethe1, mt-Co2, Acot1, Mlycd	80	4.7	9.01E-29
oxidation-reduction process	Cox5a, Sdhd, Oxa1l, Idh3g, Gnmt, Cd36, Atp5d, Gcdh, Gys1, Etfb, Pex5, Insr, Cs, Trap1, Inpp5k, Sod2, Mgst1, Sdhb, Plin5, Ndufs2, Slc25a13, Cox7c, Acadvl, Mdh2, Ndufs7, Appl2, Mdh1, Ogdh, Dld, Acox1, Eci2, Sdha, Pdhb, Ogdhl, Sucla2, Dhrs4, Aco2, Apod, Cyc1, Gbe1, Gsk3b, Ndufa5, Ndufv2, Eci1, Hsd17b4, Prdx5, Shmt2, Echs1, Uqcrc1, Hadha, Rb1cc1, Ndufs1, Ndufa10, Fh1, Phyh, Slc25a12, Cat, Ivd, Idh3b, Etfdh, Hadh, Abcd3, Decr1, Aldob, Echdc2, Cpt2, Pink1, Fabp3, Sirt4, Acads, Me3, Dgat2, Cox6a2, Coq9, Cox4i1, Acat1, Idh3a, Etfa, Pygb, Aldh5a1, Acaa2, Ndufv1, Coq10a, Mybbp1a, Acacb, Uqcrq, Bdnf, Suclg1, Aldh1a1, Adgrf5, Foxk1, Retsat, Gm10053, Hadhb, Uqcr10, Ndufs8, Cyp2d22, Suclg2, Ppox, Acadm, mt-Nd1, mt-Co1, mt-Co2, mt-Co3, mt-Nd4, Ndufb6, H2-Ke6, Mlycd, Adh1	109	4.68	3.86E-40
mitochondrial translation	Uqcc1, Yars2, Lrpprc, Mrps18b, Mrpl16, Shmt2, Mrpl44, Dars2, Gfm1, Nsun4, Rpusd4, Mtg1, Trub2, Mrps27, Rcc1l, Tufm	16	4.59	0.000558
lipid modification	Cd36, Gcdh, Etfb, Pex5, Inpp5k, Plin5, Acadvl, Appl2, Efr3b, Acox1, Eci2, Ephx2, Apod, Eci1, Hsd17b4, Dgka, Echs1, Hadha, Phyh, Ivd, Etfdh, Hadh, Abcd3, Decr1, Echdc2, Cpt2, Fabp3, Sirt4, Acads, Dgat2, Acat1, Etfa, Ip6k1, B3galt1, Ggta1, Acaa2, Inpp4b, Dgkz, Acacb, Hadhb, Acadm, Dgkd, Mlycd	43	4.58	5.48E-14
sulfur compound biosynthetic process	Dlat, Gcdh, Ggt5, Pdhx, Acsl1, Dld, Pdhb, Csad, Mpc2, Acss1, Acss2, Gss, Acat1, Pdk2, Acacb, Mlycd	16	4.46	0.000823
regulation of striated muscle contraction	Jup, Atp1a2, Nkx2-5, Ryr2, Gata4, Dsc2, Casq2, Scn5a, Ank2, Rangrf, Tnni3, Pln, Tnni3k, Nppa, Dsg2, Cacna1c, Dsp	17	4.4	0.000453
cellular lipid catabolic process	Lipe, Gcdh, Etfb, Pex5, Plin5, Acadvl, Acox1, Acot2, Eci2, Prkcd, Cyp1b1, Eci1, Hsd17b4, Echs1, Pnpla2, Hadha, Phyh, Ivd, Pck1, Etfdh, Hadh, Abcd3, Decr1, Echdc2, Cpt2, Acot7, Acads, Acat1, Etfa, Acaa2, Acacb, Sorl1, Hadhb, Acadm, Mlycd	35	4.34	2.87E-10
actin-mediated cell contraction	Jup, Atp1a2, Ryr2, Gata4, Dsc2, Epb41l5, Tnnt2, Casq2, Kcnj5, Scn5a, Ank2, Rangrf, Pln, Myh6, Dsg2, Scn4b, Cacna1c, Dsp	18	4.19	0.000425
mitochondrial gene expression	Tbrg4, Tfam, Uqcc1, Yars2, Lrpprc, Mrps18b, Mrpl16, Shmt2, Mrpl44, Dars2, Gfm1, Nsun4, Rpusd4, Mtg1, Trub2, Mrps27, Chchd10, Rcc1l, Tufm	19	4.11	0.000274
pyruvate metabolic process	Dlat, Stat3, Insr, Slc4a1, Pdhx, Ddit4, Dld, Pdhb, Zbtb20, Mpc2, Pck1, Aldob, Ldhb, Me3, Mpi, Mif, Hk1, Pdk2, Foxk1, Fbp1	20	4	0.000206
fatty acid metabolic process	Alkbh7, Cd36, Lipe, Gcdh, Gstm7, Etfb, Pex5, Ggt5, Mid1ip1, Plin5, Acsf3, Ptgds, Acadvl, Acsl1, Appl2, Dld, Acox1, Acot2, Eci2, Ephx2, Cyp1b1, Eci1, Hsd17b4, Gk, Echs1, Hadha, Phyh, Crat, Ivd, Acss1, Pck1, Acss2, Etfdh, Hadh, Abcd3, Decr1, Echdc2, Cpt2, Fabp3, Mecr, Acot7, Sirt4, Acads, Dgat2, Acsm5, Slc27a1, Gnpat, Acat1, Etfa, Mif, Acot11, Acaa2, Pdk2, Acacb, Acot4, Hadhb, Cyp2d22, Acadm, Acot1, H2-Ke6, Mlycd	61	3.86	1.03E-16
regulation of heart rate	Jup, Ryr2, Atp5j, Epas1, Dsc2, Casq2, Kcnj5, Scn5a, Ank2, Tnni3k, Myh6, Nppa, Dsg2, Scn4b, Cacna1c, Dsp	16	3.86	0.006474
actin filament-based movement	Jup, Atp1a2, Ryr2, Gata4, Dsc2, Epb41l5, Tnnt2, Casq2, Myh14, Myo7a, Kcnj5, Scn5a, Ank2, Rangrf, Pln, Myh6, Dsg2, Scn4b, Cacna1c, Dsp	20	3.82	0.000455
cardiac cell development	Fhl2, Myl2, Nkx2-5, Gata4, Gsk3b, Jag1, Foxp1, Csrp3, Slc25a4, Mylk3, Alpk2, Myom3, Myh6, Actn2, Nebl, Akap13	16	3.69	0.011819
cardiac muscle cell development	Fhl2, Myl2, Nkx2-5, Gata4, Gsk3b, Foxp1, Csrp3, Slc25a4, Mylk3, Alpk2, Myom3, Myh6, Actn2, Nebl, Akap13	15	3.66	0.02568
protein homotetramerization	Gnmt, Pex5, Sod2, Appl2, Shmt2, Cat, Decr1, Acads, Aldh5a1, Acacb, Actn2, Aldh1a1, Impdh2, Acadm, Fbp1	15	3.61	0.029627
lipid catabolic process	Lipe, Gcdh, Etfb, Pex5, Plin5, Acadvl, Fmc1, Acox1, Acot2, Eci2, Prkcd, Cyp1b1, Eci1, Hsd17b4, Cidea, Echs1, Pnpla2, Hadha, Sctr, Phyh, Ivd, Pck1, Etfdh, Hadh, Abcd3, Decr1, Echdc2, Cpt2, Acot7, Acads, Acat1, Etfa, Acaa2, Acacb, Sorl1, Hadhb, Acadm, Mlycd	38	3.58	1.37E-08
monocarboxylic acid metabolic process	Dlat, Alkbh7, Cd36, Lipe, As3mt, Gcdh, Gstm7, Stat3, Etfb, Pex5, Insr, Ggt5, Slc4a1, Mid1ip1, Pdhx, Plin5, Acsf3, Ptgds, Acadvl, Acsl1, Ddit4, Appl2, Dld, Acox1, Acot2, Eci2, Pdhb, Ephx2, Zbtb20, Cyp1b1, Eci1, Hsd17b4, Gk, Echs1, Hadha, Mpc2, Phyh, Crat, Ivd, Acss1, Pck1, Acss2, Etfdh, Hadh, Abcd3, Decr1, Aldob, Echdc2, Cpt2, Fabp3, Mecr, Acot7, Sirt4, Acads, Ldhb, Me3, Fah, Dgat2, Acsm5, Slc27a1, Ldhd, Gnpat, Acat1, Mpi, Etfa, Mif, Acot11, Aldh5a1, Acaa2, Hk1, Pdk2, Acacb, Idnk, Acot4, Aldh1a1, Foxk1, Abat, Hadhb, Cyp2d22, Acadm, Rdh16, Fbp1, Acot1, H2-Ke6, Mlycd, Adh1	86	3.56	5.18E-22

Supplemental Table 3. Continued

Pathway/Function Categories	Genes	Gene Count	Fold Enrichment	Adjusted *p*-value
	GO_Biological Process-Upregulated in str*Nrip1*$^{-/-}$			
mitochondrial membrane organization	Oxa1l, Alkbh7, Stat3, Pex5, Ppif, Samm50, Gsk3b, Afg3l2, Mfn1, Pink1, Slc25a4, Acaa2, Chchd10, Immt, Chchd3, Rcc1l	16	3.46	0.027271
cellular component assembly involved in morphogenesis	Myl2, Nkx2-5, Cflar, Tnnt2, Casq2, Foxp1, Csrp3, Mylk3, Gnpat, Myom3, Myh6, Pgm5, Actn2, Nebl, Akap13, Acrbp	16	3.43	0.031126
cellular amino acid metabolic process	Gnmt, Ggt5, Dglucy, Aldh6a1, Fars2, Mccc2, Glud1, Yars2, Gart, Csad, Mmut, Got1, Shmt2, Adhfe1, Dars2, Ivd, Ahcy, Tars2, Hmgcl, Sirt4, Hibadh, Fah, Bckdk, Bcat2, Oat, Got2, Acat1, Bckdhb, Aldh5a1, Iars, Kyat3, Pars2, Abat, Cyp2d22, Ddo, Ass1	36	3.41	2.12E-07
organic acid metabolic process	Dlat, Idh3g, Alkbh7, Gnmt, Cd36, Lipe, As3mt, Gcdh, Gstm7, Stat3, Etfb, Dlst, Pex5, Insr, Cs, Ggt5, Slc4a1, Mid1ip1, Sdhb, Pdhx, Plin5, Acsf3, Ptgds, Acadvl, Acsl1, Mdh2, Ddit4, Appl2, Mdh1, Ogdh, Dld, Acox1, Dglucy, Acot2, Aldh6a1, Eci2, Fars2, Sdha, Mccc2, Pdhb, Glud1, Ephx2, Sucla2, Aco2, Zbtb20, Yars2, Gart, Csad, Mmut, Cyp4f13, Cyp1b1, Eci1, Hsd17b4, Gk, Got1, Shmt2, Echs1, Hadha, Adhfe1, Fh1, Mpc2, Phyh, Mgst3, Dars2, Crat, Ivd, Idh3b, Acss1, Pck1, Ahcy, Acss2, Etfdh, Hadh, Tars2, Abcd3, Decr1, Aldob, Echdc2, Cpt2, Hmgcl, Fabp3, Mecr, Acot7, Sirt4, Acads, Hibadh, Ldhb, Idh2, Me3, Fah, Dgat2, Bckdk, Bcat2, Oat, Acsm5, Got2, Il15, Slc27a1, Ldhd, Gnpat, Acat1, Bckdhb, Idh3a, Mpi, Etfa, Mif, Acot11, Aldh5a1, Amdhd2, Acaa2, Hk1, Iars, Pdk2, Kyat3, Acacb, Pars2, Ndnf, Idnk, Acot4, Suclg1, Aldh1a1, Foxk1, Abat, Hadhb, Cyp2d22, Suclg2, Acadm, Ddo, Rdh16, Fbp1, Acot1, H2-Ke6, Mlycd, Adh1, Ass1	135	3.36	1.15E-33
regulation of heart contraction	Tbx2, Jup, Atp1a2, Myl2, Nkx2-5, Ryr2, Gata4, Atp5j, Epas1, Dsc2, Tnnt2, Casq2, Csrp3, Kcnj5, Scn5a, Ank2, Rangrf, Tnni3, Pln, Tnni3k, Myh6, Nppa, Dsg2, Scn4b, Cacna1c, Dsp, Tmem65	27	3.35	6.76E-05
alpha-amino acid metabolic process	Gnmt, Dglucy, Aldh6a1, Mccc2, Glud1, Gart, Csad, Mmut, Got1, Shmt2, Adhfe1, Ivd, Ahcy, Hmgcl, Sirt4, Hibadh, Fah, Bcat2, Oat, Got2, Acat1, Aldh5a1, Kyat3, Cyp2d22, Ddo, Ass1	26	3.24	0.000234
mitochondrion organization	Ndufa9, Oxa1l, Poldip2, Ndufb2, Alkbh7, Timm44, Atp5d, Tfam, Stat3, Dnaja3, Phb2, Pex5, Uqcc1, Sod2, Ndufa1, Fmc1, Ndufs7, Vdac1, Sept4, Ppif, Ndufb9, Samm50, Gsk3b, Ndufa5, Epas1, Afg3l2, Timm21, Ndufb8, Rb1cc1, Ndufs1, Ndufa10, Ndufa8, Map1lc3a, Mfn1, Hmgcl, Pink1, Cdk5, Pmpcb, Atad3a, Grpel1, Sirt4, Myh14, Ndufab1, Uqcrc2, Slc25a4, Map1lc3b, Ndufa3, Acaa2, Aifm1, Phb, Foxred1, Ndufb10, Lonp1, Wipi1, Wdr81, Cd24a, Chchd10, Immt, Chchd3, Uqcr10, Ndufs8, Rcc1l, Htra2, Ndufb6, Cox7a1	65	3.05	1.53E-12
protein tetramerization	Oxa1l, Gnmt, Pex5, Insr, Sod2, Appl2, Dhrs4, Shmt2, Cat, Decr1, Hmgcl, Acads, Rrm1, Aldh5a1, Acacb, Actn2, Aldh1a1, Hist1h4h, Impdh2, Acadm, Fbp1, H2-Ke6	22	3.01	0.007803
muscle cell development	Fhl2, Myl2, Nkx2-5, Gata4, Bin3, Hes1, Gsk3b, Afg3l2, Cflar, Tnnt2, Nfatc2, Casq2, Foxp1, Csrp3, Slc25a4, Mylk3, Ank2, Alpk2, Myom3, Myh6, Pgm5, Actn2, Nebl, Naca, Akap13	25	2.98	0.002116
heart process	Tbx2, Jup, Atp1a2, Myl2, Nkx2-5, Ryr2, Gata4, Atp5j, Epas1, Dsc2, Vegfb, Tnnt2, Casq2, Csrp3, Kcnj5, Scn5a, Ank2, Rangrf, Tnni3, Pln, Tnni3k, Myh6, Nppa, Dsg2, Scn4b, Cacna1c, Yap1, Dsp, Tmem65, Akap13	30	2.96	0.000209
actomyosin structure organization	Rac1, Inpp5k, Myl2, Nkx2-5, Cdc42bpb, Cflar, Epb41l5, Tnnt2, Prkcq, Frmd5, Casq2, 2310002L09Rik, Foxp1, Csrp3, Myh14, Mylk3, Myom3, F11r, Myh6, Pgm5, Kank3, S1pr1, Actn2, Nebl, Akap13	25	2.96	0.002352
mitochondrial transport	Oxa1l, Alkbh7, Timm44, Stat3, Slc25a13, Ppif, Samm50, Cyc1, Gsk3b, Atp5o, Afg3l2, Timm21, Atp5b, Mpc2, Slc25a12, Pink1, Pmpcb, Grpel1, Sirt4, Uqcrc2, Slc25a4, Slc8b1, Acaa2, Slc25a28, Chchd10	25	2.91	0.003212
heart contraction	Tbx2, Jup, Atp1a2, Myl2, Nkx2-5, Ryr2, Gata4, Atp5j, Epas1, Dsc2, Vegfb, Tnnt2, Casq2, Csrp3, Kcnj5, Scn5a, Ank2, Rangrf, Tnni3, Pln, Tnni3k, Myh6, Nppa, Dsg2, Scn4b, Cacna1c, Dsp, Tmem65	28	2.9	0.000866
cardiac chamber development	Tbx2, Gata6, Fhl2, Myl2, Nkx2-5, Ryr2, Gata4, Tab1, Hes1, Tnnt2, Jag1, Ccm2l, Tbx20, Scn5a, Hectd1, Tnni3, Hand2, Myh6, Ndst1, Dsp, Cntrl, Naca, Tmem65	23	2.85	0.011937
regulation of muscle contraction	Jup, Atp1a2, Nkx2-5, Ryr2, Gata4, Dsc2, Tnnt2, Casq2, Scn5a, Ank2, Rangrf, Tnni3, Pln, Tnni3k, Nppa, Dsg2, Chrm2, Cacna1c, Dsp, Abat	20	2.85	0.049234
cellular ketone metabolic process	Ndufa9, Mid1ip1, Plin5, Stard3, Acadvl, Appl2, Coq6, Oxct1, Dhrs4, Gk, Coq8a, Fabp3, Sirt4, Dgat2, Coq9, Pdk2, Bmp6, Coq10a, Kyat3, Coq5, Acacb, Sirt5, Afp, Mlycd	24	2.8	0.010662
cardiocyte differentiation	Tbx2, Efnb2, Gata6, Fhl2, Myl2, Nkx2-5, Gata4, Hes1, Gsk3b, Jag1, Foxp1, Csrp3, Slc25a4, Mylk3, Alpk2, Myom3, Hand2, Myh6, Actn2, Nebl, Acadm, Akap13	22	2.78	0.02917
monovalent inorganic cation transport	Atp5d, Phb2, Clcn3, Aqp1, Atp5g1, Atp1a2, Nkx2-5, Slc12a7, Atp5g3, Ndufs7, Ppif, Cyc1, Atp5o, Atp5b, Atp5a1, Atp5c1, Kcnj3, Stom, Casq2, Kcnq5, Kcnq4, Tesc, Slc25a4, Kcnj5, Scn5a, Slc8b1, Ank2, Rangrf, Mif, Kcnk1, Atp5h, Fxyd7, Mllt6, Atp5l, Plcb4, Nppa, Scn4b, Kcnk3, Chchd10, Actn2, Kcng2, mt-Co1, mt-Atp6, mt-Nd4, mt-Cytb, Fxyd6	46	2.66	3.04E-06
cellular lipid metabolic process	Rac1, Alkbh7, Cd36, Lipe, Gcdh, Gstm7, Etfb, Pex5, Gata6, Inpp5k, Ggt5, Mid1ip1, Vav2, Plin5, Acsf3, Ptgds, Cers2, Acadvl, Acsl1, Appl2, Efr3b, Dld, Acox1, Acot2, Fdft1, Eci2, Prkcd, Ephx2, Dhrs4, Apod, Chkb, Pisd, Cyp1b1, Eci1, Hsd17b4, Gk, Dgka, Echs1, Pnpla2, Hadha, Phyh, St6galnac6, Crat, Cat, Ivd, Acss1, Pck1, Acss2, Etfdh, Hmgcs2, Pmvk, Hadh, Acp6, Abcd3, Decr1, Echdc2, Cpt2, Fabp3, Mecr, Acot7, Gpat3, Cds1, Sirt4, Acads, Dgat2, Acsm5, Slc27a1, Gnpat, Acat1, Etfa, Ip6k1, Mif, B3galt1, Acot11, Ggta1, Cerk, Acaa2, Inpp4b, Pdk2, Pitpnc1, Dgkz, Acacb, Wdr81, Sorl1, Acot4, B4galt3, Aldh1a1, Adgrf5, Retsat, Hdhd5, Hadhb, Cyp2d22, Acadm, Rdh16, Dgkd, Acot1, H2-Ke6, Mlycd, Adh1, Pigb	100	2.58	3.2E-15
regulation of blood circulation	Tbx2, Jup, Atp1a2, Myl2, Nkx2-5, Ryr2, Gata4, Atp5j, Epas1, Dsc2, Tnnt2, Casq2, Csrp3, Kcnj5, Scn5a, Ank2, Rangrf, Tnni3, Pln, Tnni3k, Myh6, Nppa, Dsg2, Scn4b, Cacna1c, Dsp, Tmem65	27	2.52	0.020023
regulation of muscle system process	Jup, Atp1a2, Nkx2-5, Akap1, Ryr2, Gata4, Fbxo32, Gsk3b, Dsc2, Tnnt2, Casq2, Errfi1, Foxp1, Slc25a4, Scn5a, Ank2, Rangrf, Tnni3, Hand2, Pln, Adk, Tnni3k, Nppa, Dsg2, Chrm2, Cacna1c, Dsp, Abat	28	2.51	0.015669
muscle contraction	Jup, Atp1a2, Myl2, Nkx2-5, Ryr2, Gata4, Dsc2, Vegfb, Tnnt2, Casq2, Csrp3, Myh14, Tbx20, Kcnj5, Scn5a, Ank2, Rangrf, Tnni3, Myom3, Pln, Tnni3k, Myh6, Nppa, Dsg2, Chrm2, Scn4b, Cacna1c, Actn2, Dsp, Myh1, Abat	31	2.45	0.008057
heart morphogenesis	Tbx2, Insr, Gata6, Fhl2, Myl2, Nkx2-5, Dvl2, Ryr2, Gata4, Tab1, Hes1, Mib1, Tnnt2, Jag1, Ccm2l, Wnt16, Tbx20, Alpk2, Tnni3, Myom3, Hand2, Myh6, Asxl1, S1pr1, Fat4, C2cd3, Yap1, Dsp, Naca	29	2.45	0.016772
cellular carbohydrate metabolic process	Gnmt, Gys1, Stat3, Insr, Inpp5k, Plcd1, Ddit4, Gbe1, Gsk3b, Ddb1, Gk, Got1, Rb1cc1, Wdr5, Pck1, Rorc, Dgat2, Ip6k1, Pygb, Hk1, Inpp4b, Pdk2, Acacb, Idnk, Ndst1, Foxk1, Acadm, Clk2, Fbp1, Mlycd	30	2.44	0.013053
muscle system process	Jup, Gata6, Atp1a2, Myl2, Nkx2-5, Akap1, Ryr2, Gata4, Fbxo32, Gsk3b, Dsc2, Vegfb, Cflar, Tnnt2, Casq2, Errfi1, Foxp1, Csrp3, Myh14, Slc25a4, Tbx20, Kcnj5, Scn5a, Ank2, Rangrf, Tnni3, Myom3, Hand2, Pln, Adk, Tnni3k, Myh6, Nppa, Dsg2, Chrm2, Scn4b, Cacna1c, Actn2, Dsp, Myh1, Abat, Akap13	42	2.36	0.000492
striated muscle tissue development	Tbx2, Efnb2, Gata6, Fhl2, Myl2, Nkx2-5, Erbb3, Btg2, Ryr2, Gata4, Bin3, Hdac7, Gsk3b, Ndufv2, Ankrd2, Cflar, Tnnt2, Ccm2l, Cdk5, Foxp1, Csrp3, Myh14, Slc25a4, Mylk3, Tbx20, Alpk2, Tnni3, Myom3, Pln, Myh6, Pgm5, Maff, Dsg2, S1pr1, Bdnf, Actn2, Yap1, Nebl, Dsp, Naca, Acadm, Akap13, Med20	43	2.31	0.000583
circulatory system process	Tbx2, Jup, Cd36, Sod2, Atp1a2, Bcr, Myl2, Nkx2-5, Fli1, Nampt, Ryr2, Gata4, Ephx2, Atp5j, Epas1, Dsc2, Vegfb, Tnnt2, Casq2, Csrp3, Mylk3, Cdh5, Tbx20, Kcnj5, Scn5a, Ank2, Rangrf, Ptprm, Mif, Tnni3, F11r, Pln, Bmp6, Tnni3k, Myh6, Nppa, Amot, Dsg2, Scn4b, Cacna1c, Yap1, Klk1b26, Dsp, Abat, Ptp4a3, Tmem65, Akap13	47	2.18	0.001005
inorganic cation transmembrane transport	Atp5d, Gstm7, Phb2, Clcn3, Atp5g1, Atp1a2, Plcd1, Slc41a1, Plcg1, Slc12a7, Atp5g3, Ndufs7, Vdac1, Ryr2, Ppif, Cyc1, Atp5o, Afg3l2, Atp5b, Atp5a1, Atp5c1, Slc40a1, Kcnj3, Stom, Casq2, Kcnq5, Kcnq4, Tesc, Itpr2, Stim1, Slc25a4, Kcnj5, Gnb5, Scn5a, Slc8b1, Ank2, Rangrf, Kcnk1, Atp5h, Fxyd7, Slc24a2, Pln, Atp5l, Plcb4, Slc25a28, Nppa, Scn4b, Kcnk3, Chchd10, Cacna1c, Actn2, Kcng2, mt-Co1, mt-Atp6, mt-Nd4, mt-Cytb, Fxyd6	57	2.15	9.64E-05
blood circulation	Tbx2, Jup, Cd36, Sod2, Atp1a2, Bcr, Myl2, Nkx2-5, Fli1, Nampt, Ryr2, Gata4, Ephx2, Atp5j, Epas1, Dsc2, Vegfb, Tnnt2, Casq2, Csrp3, Mylk3, Cdh5, Tbx20, Kcnj5, Scn5a, Ank2, Rangrf, Ptprm, Mif, Tnni3, F11r, Pln, Bmp6, Tnni3k, Myh6, Nppa, Amot, Dsg2, Scn4b, Cacna1c, Klk1b26, Dsp, Abat, Ptp4a3, Tmem65	45	2.13	0.002977
lipid biosynthetic process	Mid1ip1, Plin5, Acsf3, Ptgds, Cers2, Stard3, Acadvl, Acsl1, Fdft1, Prkcd, Chkb, Zbtb20, Pisd, Dgka, St6galnac6, Acss1, Pck1, Acss2, Hmgcs2, Pmvk, Abcd3, Fabp3, Acot7, Gpat3, Cds1, Sirt4, Dgat2, Acsm5, Slc27a1, Gnpat, Sc5d, Mif, Dhrs11, B3galt1, Bmp6, Dgkz, Acacb, B4galt3, Bmpr1b, Aldh1a1, Adgrf5, Hdhd5, Rdh16, H2-Ke6, Mlycd, Pigb	46	2.09	0.004147
response to inorganic substance	Cd36, Aqp1, Trap1, Slc4a1, Sod2, Plcd1, Slc41a1, Slc25a13, Mb, Ryr2, Glud1, Anxa7, Ppif, Prkcd, Gsk3b, Gart, Cyp1b1, Slc40a1, Cflar, Tnnt2, Lcn2, Slc25a12, Cat, Gss, Aldob, Pink1, Foxp1, Fus, Stim1, Scn5a, Szt2, Ccs, Pln, Bmp6, Timeless, Junb, Abat, mt-Cytb, Fbp1, Ass1, Ddi2	41	2.07	0.019566
protein complex oligomerization	Oxa1l, Jup, Clpp, Gnmt, Pex5, Insr, Slc4a1, Sod2, Mgst1, Appl2, Sept4, Chrna2, Dhrs4, Shank3, Cdh2, Tmem173, Shmt2, Tnnt2, Lcn2, Stom, Cat, Decr1, Hmgcl, Acads, Oat, Rrm1, Acat1, Malt1, Mif, Aldh5a1, Lzts3, Pln, Tmem120a, Lonp1, Acacb, Sorl1, Actn2, Aldh1a1, Sept11, Hist1h4h, Impdh2, Acadm, Htra2, Fbp1, Dgkd, H2-Ke6	46	2.06	0.006206
carbohydrate metabolic process	Gnmt, Gys1, Stat3, Insr, Cs, Inpp5k, Slc4a1, Plcd1, Ddit4, Apod, Gbe1, Zbtb20, Gsk3b, Ddb1, Gk, Got1, Fuom, Rb1cc1, St6galnac6, Wdr5, Pck1, Crtc2, Rorc, Aldob, Dgat2, Mpi, Ip6k1, Pygb, Mif, B3galt1, Hk1, Inpp4b, Pdk2, Acacb, Idnk, Ndst1, Foxk1, Acadm, Clk2, Fbp1, Mlycd, Amy1	42	2.06	0.016846
multicellular organismal homeostasis	Rac1, Kmt2a, Cd36, Stat3, Clcn3, Aqp1, Prlr, Inpp5k, Acadvl, Dync1h1, Acsl1, Appl2, Ahsg, Epas1, Cidea, Prdx5, Ldb1, Homer2, Slc40a1, Sctr, Fh1, Lcn2, Car2, Hadh, Abca4, Decr1, Sgip1, Cpt2, Pgam5, Il15, Irx3, Slc27a1, Ip6k1, Acot11, Tns2, Bmp6, Wfs1, Acacb, S1pr1, Yap1, Aldh1a1, Adgrf5, Abat	43	2.05	0.014492
cation transmembrane transport	Atp5d, Gstm7, Phb2, Clcn3, Atp5g1, Atp1a2, Plcd1, Slc41a1, Plcg1, Slc12a7, Atp5g3, Ndufs7, Vdac1, Ryr2, Ppif, Cyc1, Shank3, Atp5o, Afg3l2, Atp5b, Atp5a1, Atp5c1, Slc40a1, Kcnj3, Stom, Casq2, Kcnq5, Kcnq4, Pink1, Tesc, Itpr2, Stim1, Slc25a4, Kcnj5, Gnb5, Scn5a, Slc25a20, Slc8b1, Ank2, Rangrf, Kcnk1, Atp5h, Fxyd7, Slc24a2, Pln, Atp5l, Plcb4, Slc25a28, Nppa, Scn4b, Kcnk3, Chchd10, Cacna1c, Actn2, Kcng2, mt-Co1, mt-Atp6, mt-Nd4, mt-Cytb, Fxyd6	60	2.01	0.000461
ion transmembrane transport	Slc25a42, Atp5d, Gstm7, Phb2, Clcn3, Atp5g1, Slc5a6, Atp1a2, Plcd1, Slc41a1, Slc25a13, Plcg1, Slc12a7, Atp5g3, Slc16a7, Ndufs7, Vdac1, Ryr2, Ppif, Prkcd, Chrna2, Cyc1, Shank3, Atp5o, Afg3l2, Atp5b, Atp5a1, Atp5c1, Slc40a1, Mpc2, Kcnj3, Stom, Slc25a12, Casq2, Kcnq5, Kcnq4, Pink1, Tesc, Itpr2, Stim1, Slc25a4, Kcnj5, Gnb5, Scn5a, Slc25a20, Slc8b1, Ank2, Rangrf, Kcnk1, Atp5h, Fxyd7, Hk1, Slc20a2, Slc24a2, Pln, Atp5l, Plcb4, Slc25a28, Nppa, Lrrc8d, Scn4b, Kcnk3, Chchd10, Cacna1c, Actn2, Kcng2, Slc38a10, Slc25a3, mt-Co1, mt-Atp6, mt-Nd4, mt-Cytb, Chrna10, Fxyd6	74	2	1.88E-05

Supplemental Table 3. Continued

Pathway/Function Categories	Genes	Gene Count	Fold Enrichment	Adjusted *p*-value
**KEGG_PATHWAY-Upregulated in str*Nrip1*$^{-/-}$**				
Citrate cycle (TCA cycle)	Dlat, Sdhd, Idh3g, Dlst, Cs, Sdhb, Mdh2, Mdh1, Ogdh, Dld, Sdha, Pdhb, Ogdhl, Sucla2, Aco2, Fh1, Idh3b, Pck1, Idh2, Idh3a, Suclg1, Suclg2	22	10.34	3.24E-17
Propanoate metabolism	Dbt, Dld, Acox1, Aldh6a1, Sucla2, Mmut, Echs1, Hadha, Acss1, Acss2, Acads, Ldhb, Acat1, Bckdhb, Pccb, Hibch, Acacb, Suclg1, Abat, Bckdha, Suclg2, Mlycd	22	10.03	9.12E-17
Valine, leucine and isoleucine degradation	Dbt, Acsf3, Dld, Aldh6a1, Auh, Mccc2, Oxct1, Mmut, Hsd17b10, Echs1, Hadha, Aldh9a1, Ivd, Mccc1, Hmgcs2, Hadh, Hmgcl, Aldh2, Acads, Hibadh, Bcat2, Acadsb, Acad8, Acat1, Bckdhb, Pccb, Aldh1b1, Acaa2, Hibch, Aldh7a1, Abat, Hadhb, Bckdha, Acadm	34	9.3	8.9E-25
2-Oxocarboxylic acid metabolism	Idh3g, Cs, Aco2, Gpt, Acy1, Got1, Idh3b, Idh2, Bcat2, Got2, Idh3a	11	8.71	5.27E-07
Pyruvate metabolism	Dlat, Mdh2, Mdh1, Dld, Pdhb, Fh1, Aldh9a1, Acss1, Pck1, Acss2, Aldh2, Ldhb, Me3, Ldhd, Acat1, Aldh1b1, Acacb, Aldh7a1	18	7.13	5.28E-10
Oxidative phosphorylation	Cox5a, Sdhd, Ndufa9, Ndufb2, Atp5d, Atp5g1, Sdhb, Ndufs2, Cox6c, Ndufa1, Cox7c, Atp5g3, Ndufs7, Uqcr11, Sdha, Ndufb9, Cyc1, Atp5j, Atp5o, Ndufa5, Ndufv2, Ndufb8, Atp5b, Cox8b, Atp5c1, Ndufs1, Ndufa10, Ndufa8, Ndufa4, Cox6a2, Ndufab1, Uqcrc2, Cox7b, Cox4i1, Atp5h, Ndufa3, Cox6b1, Ndufv1, Uqcrfs1, Atp5j2, Atp5l, Ndufb10, Uqcrq, Uqcr10, Ndufs8, Cox5b, mt-Nd1, mt-Nd2, mt-Co1, mt-Co2, mt-Atp8, mt-Atp6, mt-Co3, mt-Nd4, mt-Nd5, mt-Nd6, mt-Cytb, mt-Nd4l, Ndufb6, Cox7a1	60	7.11	1.91E-35
Fatty acid degradation	Gcdh, Acadvl, Acsl1, Acox1, Eci2, Eci1, Echs1, Hadha, Acadl, Aldh9a1, Hadh, Cpt2, Aldh2, Acads, Acadsb, Acat1, Aldh1b1, Acaa2, Aldh7a1, Hadhb, Acadm, Adh1, Cpt1b	23	6.92	1.19E-12
Butanoate metabolism	L2hgdh, Oxct1, Echs1, Hadha, Hmgcs2, Hadh, Hmgcl, Acads, Acsm5, Acat1, Aldh5a1, Abat	12	6.69	5.12E-06
Glyoxylate and dicarboxylate metabolism	Cs, Mdh2, Mdh1, Dld, Aco2, Mmut, Shmt2, Cat, Acss1, Acss2, Acat1, Pccb	12	6.02	2.12E-05
beta-Alanine metabolism	Acox1, Aldh6a1, Echs1, Hadha, Aldh9a1, Aldh2, Acads, Aldh1b1, Hibch, Aldh7a1, Abat, Mlycd	12	5.82	3.25E-05
Proximal tubule bicarbonate reclamation	Car4, Aqp1, Atp1a2, Atp1b4, Mdh1, Glud1, Pck1, Car2	8	5.47	0.005634
Carbon metabolism	Dlat, Sdhd, Idh3g, Dlst, Cs, Sdhb, Mdh2, Mdh1, Ogdh, Dld, Acox1, Aldh6a1, Sdha, Pdhb, Glud1, Ogdhl, Sucla2, Aco2, Gpt, Mmut, Got1, Shmt2, Echs1, Hadha, Fh1, Cat, Idh3b, Acss1, Acss2, Aldob, Acads, Idh2, Me3, Got2, Acat1, Idh3a, Pccb, Hk1, Hibch, Idnk, Suclg1, Suclg2, Fbp1	43	5.39	3.45E-19
Cardiac muscle contraction	Cox5a, Atp1a2, Myl2, Cox6c, Atp1b4, Cox7c, Uqcr11, Ryr2, Cyc1, Cox8b, Tnnt2, Casq2, Cox6a2, Uqcrc2, Cox7b, Cox4i1, Tnni3, Cox6b1, Uqcrfs1, Myh6, Uqcrq, Cacna1c, Uqcr10, Cox5b, mt-Co1, mt-Co2, mt-Co3, mt-Cytb, Cox7a1	29	5.32	1.77E-12
Fatty acid elongation	Acot2, Echs1, Hadha, Hadh, Mecr, Acot7, Acaa2, Acot4, Hadhb, Acot1	10	5.19	0.001098
Tryptophan metabolism	Inmt, Gcdh, Dlst, Dld, Cyp1b1, Echs1, Hadha, Aldh9a1, Cat, Hadh, Aldh2, Acat1, Aldh1b1, Kyat3, Aldh7a1	15	4.8	2.08E-05
Thermogenesis	Cox5a, Sdhd, Ndufa9, Ndufb2, Atp5d, Lipe, Atp5g1, Sdhb, Ndufs2, Cox6c, Ndufa1, Cox7c, Atp5g3, Acsl1, Ndufs7, Uqcr11, Adcy2, Sdha, Ndufb9, Cyc1, Atp5j, Atp5o, Ndufa5, Ndufv2, Ndufb8, Atp5b, Cox8b, Pnpla2, Atp5c1, Ndufs1, Ndufa10, Ndufa8, Npr1, Cpt2, Ndufa4, Cox6a2, Ndufab1, Uqcrc2, Cox7b, Cox4i1, Slc25a20, Atp5h, Ndufa3, Cox6b1, Ndufv1, Uqcrfs1, Atp5j2, Atp5l, Ndufb10, Nppa, Uqcrq, Uqcr10, Ndufs8, Cox5b, mt-Nd1, mt-Nd2, mt-Co1, mt-Co2, mt-Atp8, mt-Atp6, mt-Co3, mt-Nd4, mt-Nd5, mt-Nd6, mt-Cytb, mt-Nd4l, Ndufb6, Cox7a1, Cpt1b	69	4.7	2.46E-27
Fatty acid metabolism	Acsf3, Acadvl, Acsl1, Acox1, Hsd17b4, Echs1, Hadha, Acadl, Hadh, Cpt2, Mecr, Acads, Acadsb, Acat1, Acaa2, Hadhb, Acadm, H2-Ke6, Cpt1b	19	4.69	7.52E-07
Peroxisome	Pex6, Pex5, Sod2, Acsl1, Acox1, Eci2, Ephx2, Dhrs4, Hsd17b4, Prdx5, Phyh, Crat, Cat, Pmvk, Abcd3, Hmgcl, Pxmp2, Gstk1, Idh2, Pex11a, Nudt7, Gnpat, Ech1, Ddo, Mlycd	25	4.59	5.08E-09
Lysine degradation	Kmt2a, Gcdh, Dlst, Kmt2b, Ezh1, Dld, Echs1, Hadha, Aldh9a1, Hadh, Aldh2, Acat1, Aldh1b1, Kmt5b, Kmt2d, Aldh7a1, Dot1l	17	4.26	2.23E-05
Glycerolipid metabolism	Agpat3, Lpin1, Gk, Dgka, Pnpla2, Aldh9a1, Gpat3, Aldh2, Dgat2, Aldh1b1, Dgkz, Pnpla3, Aldh7a1, Dgkd	14	3.51	0.003131
Retrograde endocannabinoid signaling	Ndufa9, Ndufb2, Ndufs2, Ndufa1, Grm1, Ndufs7, Adcy2, Ndufb9, Ndufa5, Ndufv2, Ndufb8, Ndufs1, Ndufa10, Kcnj3, Ndufa8, Ndufa4, Itpr2, Ndufab1, Kcnj5, Gnb5, Ndufa3, Ndufv1, Plcb4, Ndufb10, Cacna1c, Ndufs8, mt-Nd1, mt-Nd2, mt-Nd4, mt-Nd5, mt-Nd6, mt-Nd4l, Ndufb6	33	3.47	1.72E-08
Glycolysis / Gluconeogenesis	Dlat, Dld, Pdhb, Aldh9a1, Acss1, Pck1, Acss2, Aldob, Aldh2, Ldhb, Aldh1b1, Hk1, Aldh7a1, Fbp1, Adh1	15	3.47	0.001875
Biosynthesis of amino acids	Idh3g, Cs, Aco2, Gpt, Acy1, Got1, Shmt2, Idh3b, Aldob, Idh2, Bcat2, Got2, Idh3a, Ass1	14	2.77	0.046269

Pathway/Function Categories	Genes	Gene Count	Fold Enrichment	Adjusted *p*-value
**GO_Biological Process_Downregulated in str*Nrip1*$^{-/-}$**				
regulation of ryanodine-sensitive calcium-release channel activity	Calm1, Jph2, Calm3, Trdn, Jsrp1, Calm2, Akap6	7	9.1	0.012402
positive regulation of cation channel activity	Cacnb3, Kif5b, Casq1, Jph2, Trdn, Galr2, Ifngr2, Asph, Stim2, Stac3, Amigo1, Akap6, Kcnj11	13	4.17	0.025812
regulation of release of sequestered calcium ion into cytosol	Calm1, Casq1, Jph2, Calm3, Trdn, Jsrp1, Asph, Calm2, Aplnr, Mettl21c, Camk2d, Akap6, Capn3	13	4.01	0.039591
regulation of calcium ion transport into cytosol	Calm1, Casq1, Jph2, Calm3, Trdn, Jsrp1, Calcr, Asph, Calm2, Aplnr, Mettl21c, Camk2d, Bak1, Akap6, Capn3	15	3.63	0.032013
regulation of cation channel activity	Calm1, Cacnb3, Mef2c, Kif5b, Casq1, Homer1, Jph2, Scn1b, Calm3, Trdn, Jsrp1, Galr2, Ifngr2, Kcnab1, Asph, Park7, Calm2, Stim2, Stac3, Amigo1, Akap6, Kcne1l, Kcnj11	23	3.34	0.00085
striated muscle contraction	Casq1, Homer1, Scn1b, Jsrp1, Sgcd, Pgam2, Map2k6, Bin1, Nos1, Synm, Tpm1, Stac3, Adora1, Camk2d, Akap6, Myl1, Cav3, Actc1, Kcne1l	19	3.09	0.027624
positive regulation of ion transmembrane transport	Cacnb3, Kif5b, Casq1, Jph2, Trdn, Galr2, Ifngr2, Calcr, Asph, Park7, Nos1, Stim2, Stac3, Atp1b2, Aplnr, Amigo1, Akap6, Capn3, Kcne1l, Kcnj11	20	2.87	0.048499
extracellular matrix organization	Fap, Myf5, Hpn, Col18a1, Lamb1, Nid1, Sulf1, Aebp1, Col14a1, Adamts20, Smoc2, Kazald1, Col5a2, Col3a1, Fn1, Col5a1, Postn, Col15a1, Eln, Mmp15, Aplp2, Plod2, Smad3, Loxl2, Adamtsl2, Adamts2, Mpzl3, Serpinh1	28	2.78	0.002342
regulation of calcium ion transport	Calm1, Cacnb3, Casq1, Homer1, Jph2, Calm3, Trdn, Igf1, Jsrp1, Ace, Calcr, Bin1, Camk2a, Asph, Nos1, Calm2, Stim2, Stac3, Aplnr, Mettl21c, Orai1, Casr, Camk2d, Bak1, Akap6, Cav3, Capn3	27	2.59	0.013236
regulation of ion transmembrane transporter activity	Calm1, Cacnb3, Mef2c, Kif5b, Casq1, Homer1, Jph2, Scn1b, Calm3, Trdn, Jsrp1, Galr2, Ifngr2, Kcnab1, Asph, Park7, Calm2, Stim2, Stac3, Atp1b2, Amigo1, Camk2d, Akap6, Cav3, Kcne1l, Kcnj11	26	2.54	0.029026
extracellular structure organization	Fap, Myf5, Hpn, Col18a1, Lamb1, Nid1, Sulf1, Aebp1, Col14a1, Adamts20, Smoc2, Kazald1, Col5a2, Col3a1, Fn1, Col5a1, Postn, Col15a1, Eln, Mmp15, Aplp2, Plod2, Smad3, Acsl3, Loxl2, Adamtsl2, Adamts2, Mpzl3, Serpinh1	29	2.46	0.017084
regulation of cation transmembrane transport	Calm1, Cacnb3, Mef2c, Kif5b, Casq1, Homer1, Jph2, Scn1b, Calm3, Trdn, Jsrp1, Galr2, Ifngr2, Calcr, Bin1, Kcnab1, Asph, Park7, Nos1, Calm2, Stim2, Stac3, Atp1b2, Aplnr, Mettl21c, Amigo1, Camk2d, Akap6, Cav3, Capn3, Kcne1l, Kcnj11	32	2.32	0.020611
divalent metal ion transport	Calm1, Slc39a13, Cacnb3, Slc30a4, Casq1, Homer1, Jph2, Calm3, Trdn, Igf1, Jsrp1, Ace, Micu2, Calcr, Bin1, Camk2a, Asph, Slc30a2, Nos1, Nipa2, Panx1, Calm2, Stim2, Stac3, Cacna2d4, Gck, Zmpste24, Aplnr, Mettl21c, Orai1, Casr, Camk2d, Bak1, Mmgt1, Akap6, Cav3, Capn3	37	2.12	0.033952
muscle structure development	Fgf6, Myf5, Srpk3, Dyrk1b, Mef2c, Casq1, Homer1, Cacybp, Jph2, Igf1, Sgcd, Pdlim7, Cxcl14, Bmpr1a, Wnt5a, Col14a1, Cby1, Akirin1, Bin1, Col3a1, Igfbp5, Xirp2, Skil, Fxr1, Serp1, Ptgfrn, Sypl2, Smarcd3, Eln, Mylpf, Sap30, Barx2, Tpm1, Smad3, Gpc1, Fhod3, Hif1an, Synpo2l, Stac3, Mamstr, Zmpste24, Myorg, Mettl21c, Plpp7, Camk2d, Akap6, Cav3, Cyp26b1, Lgals1, Actc1, Bves, Capn3	52	2.1	0.000837

The table shows the significantly enriched ($p<0.05$, adjusted *p*-value by Benjamini-Hochberg FDR method) GOTERM_BP_DIRECT and Kyoto Encyclopedia of Genes and Genomes (KEGG)_PATHWAY pathways defined by the gene sets of upregulated or downregulated genes in str*Nrip1*$^{-/-}$ RNA-seq. There are no significantly downregulated KEGG_PATHWAY in str*Nrip1*$^{-/-}$.

Supplemental Table 4. Pathways enriched in the RNA-seq from str*Nrip1*$^{-/-}$ soleus.

Pathway/Function Categories	Genes	Gene Count	Fold Enrichment	Adjusted *p*-value
**KEGG_PATHWAY-Upregulated in str*Nrip1*$^{-/-}$**				
Propanoate metabolism	Dbt, Acox1, Aldh6a1, Mmut, Echs1, Hadha, Acss1, Acss2, Ldhb, Acat1, Bckdhb, Pccb, Pcca, Abat, Suclg2	15	19.55	1.2112E-14
Valine, leucine and isoleucine degradation	Dbt, Aldh6a1, Oxct1, Mmut, Echs1, Hadha, Aldh9a1, Hmgcs2, Hadh, Aldh2, Hibadh, Bcat2, Acadsb, Acat1, Bckdhb, Pccb, Aldh1b1, Acaa2, Pcca, Aldh7a1, Abat, Hadhb, Acadm, Aox3	24	18.77	1.798E-23
Beta-Alanine metabolism	Acox1, Aldh6a1, Echs1, Hadha, Aldh9a1, Aldh2, Aldh1b1, Aldh7a1, Abat	9	12.49	1.98272E-06
Butanoate metabolism	Oxct1, Echs1, Hadha, Hmgcs2, Hadh, Acat1, Abat	7	11.15	0.000183197
Tryptophan metabolism	Inmt, Gcdh, Echs1, Hadha, Aldh9a1, Hadh, Aldh2, Acat1, Aldh1b1, Maob, Kyat3, Aldh7a1, Aox3	13	11.9	2.52889E-09
Pyruvate metabolism	Aldh9a1, Acss1, Acss2, Aldh2, Ldhb, Me3, Acat1, Aldh1b1, Aldh7a1	9	10.19	1.39264E-05
Fatty acid metabolism	Acadvl, Acsl1, Acox1, Echs1, Hadha, Acadl, Hadh, Cpt2, Acadsb, Acat1, Acaa2, Hadhb, Acadm, Cpt1b	14	9.87	6.74249E-09
Histidine metabolism	Aldh9a1, Aldh2, Aldh1b1, Maob, Aldh7a1	5	9.35	0.014341312
Glyoxylate and dicarboxylate metabolism	Mmut, Acss1, Acss2, Acat1, Pccb, Pcca	6	8.6	0.005061792
Ascorbate and aldarate metabolism	Aldh9a1, Aldh2, Aldh1b1, Aldh7a1, Ugt1a6a	5	7.96	0.031903796
Lysine degradation	Gcdh, Echs1, Hadha, Aldh9a1, Hadh, Aldh2, Aass, Acat1, Aldh1b1, Bbox1, Aldh7a1	11	7.89	9.50306E-06
Fatty acid elongation	Echs1, Hadha, Hadh, Acaa2, Hadhb	5	7.42	0.045193872

Pathway/Function Categories	Genes	Gene Count	Fold Enrichment	Adjusted *p*-value
**KEGG_PATHWAY-Downregulated in str*Nrip1*$^{-/-}$**				
Glycolysis / Gluconeogenesis	Pgam2, Tpi1, Aldoa, Pkm, Gapdh, Eno3, Pgk1, Ldha	8	11.29	3.7028E-05
Biosynthesis of amino acids	Pgam2, Tpi1, Aldoa, Gpt2, Pkm, Gapdh, Eno3, Pgk1	8	9.66	0.00012547
Glucagon signaling pathway	Prkag3, Pgam2, Camk2a, Phkg1, Pkm, Phkb, Ldha	7	6.24	0.010271723
Carbon metabolism	Pgam2, Tpi1, Aldoa, Gpt2, Pkm, Gapdh, Eno3, Pgk1	8	6.12	0.003785428
Protein digestion and absorption	Kcnq1, Slc38a2, Col9a1, Col15a1, Atp1b2, Slc8a3	6	5.92	0.042370598

Gene	Fold Change
**KEGG_PATHWAY- Downregulated genes in glycolysis/gluconeogenesis pathway in str*Nrip1*$^{-/-}$ soleus**	
Aldoa	-2.5453186
Ldha	-0.7166512
Eno3	-0.9632278
Gapdh	-0.5658315
Pkm	-0.3338802
Pgam2	-0.6781837
Pgk1	-0.3327362
Tpi1	-0.6047095

The table shows the significantly enriched (p<0.05, adjusted p-value by Benjamini-Hochberg FDR method) Kyoto Encyclopedia of Genes and Genomes (KEGG)_PATHWAY pathways defined by the gene sets of upregulated or downregulated genes in str*Nrip1*$^{-/-}$ RNA-seq.

Supplemental Table 5. Physiological parameters and echocardiographic analysis post PWAB or sham procedure in control vs str*Nrip1*$^{-/-}$ male mice.

	Sham		PWAB	
	Control	str*Nrip1*$^{-/-}$	Control	str*Nrip1*$^{-/-}$
Body weight (g)	25.8 ± 0.7	26.3 ± 0.7	25.1 ± 0.8	26.0 ± 0.7
Biventricular weight (BV; mg)	107.1 ± 3.2	120.5 ± 3.4	149.7 ± 6.4 *	128.9 ± 2.5 †
Tibia length (mm)	17.2 ± 0.3	17.3 ± 0.2	17.1 ± 0.1	17.3 ± 0.1
BV / Tibia length (mg/mm)	6.24 ± 0.14	6.95 ± 0.15	8.76 ± 0.34 *	7.45 ± 0.15 †
Heart rate (bpm)	585.6 ± 16.7	589.2 ± 11.7	582.1 ± 6.6	588.6 ± 15.8
LVPWd (mm)	0.830 ± 0.024	0.875 ± 0.022	1.144 ± 0.028 *	0.940 ± 0.025 †
IVSd (mm)	0.818 ± 0.024	0.865 ± 0.025	1.153 ± 0.031 *	0.958 ± 0.027 †
LVIDd (mm)	3.373 ± 0.094	3.483 ± 0.119	3.065 ± 0.047 *	3.347 ± 0.058 †
LVPWs (mm)	1.292 ± 0.069	1.293 ± 0.050	1.579 ± 0.040 *	1.375 ± 0.057 †
IVSs (mm)	1.301 ± 0.064	1.299 ± 0.065	1.579 ± 0.036 *	1.376 ± 0.054 †
LVIDs (mm)	1.911 ± 0.097	1.967 ± 0.143	1.704 ± 0.092	1.918 ± 0.050
LVM (mg)	92.7 ± 4.6	98.8 ± 2.9	130.6 ± 5.0 *	110.9 ± 2.2 †
LVMI (mg/g Body weight)	3.45 ± 0.10	3.82 ± 0.05	5.11 ± 0.21 *	4.23 ± 0.13 †
RWT	0.49 ± 0.02	0.50 ± 0.03	0.75 ± 0.03 *	0.57 ± 0.02 †
FS (%)	43.42 ± 1.85	43.27 ± 2.11	44.57 ± 2.47	42.67 ± 1.30
Heart rate post zatebradine (bpm)	467.6 ± 25.9	444.6 ± 12.1	473.2 ± 18.2	458.7 ± 34.9
E wave (mm/sec)	684.8 ± 40.7	692.2 ± 59.6	778.6 ± 36.3	661.0 ± 54.3
A wave (mm/sec)	592.7 ± 33.7	610.0 ± 43.8	477.3 ± 24.5	508.8 ± 54.1
e’ wave (mm/sec)	30.85 ± 1.19	31.71 ± 2.46	22.21 ± 0.88 *	26.67 ± 2.28
E/A	1.157 ± 0.040	1.132 ± 0.038	1.650 ± 0.097 *	1.332 ± 0.064 †
E/e’	22.17 ± 0.89	22.03 ± 1.55	35.17 ± 1.75 *	24.92 ± 0.78 †
EDV (µL)	40.0 ± 1.4	40.7 ± 0.9	33.8 ± 0.8 *	38.4 ± 1.1 †
ESV (µL)	11.2 ± 0.5	11.9 ± 0.5	10.1 ± 0.4	11.3 ± 0.4
EF (%)	71.9 ± 1.3	70.8 ± 1.5	70.0 ± 1.0	70.4 ± 1.1
Peak velocity (m/sec)	1.11 ± 0.10	1.00 ± 0.06	3.78 ± 0.16 *	3.89 ± 0.37 #

Echocardiography was performed on male mice 16 weeks post PWAB or sham surgery (n=5-7 per each group). E wave, A wave, e’ wave evaluation were performed after zatebradine treatment. Values are mean ± SEM. * $p < 0.05$ vs control-sham, # $p < 0.05$ vs strRIP140$^{-/-}$ - sham, † $p < 0.05$ vs control-PWAB using 2-way ANOVA with Turkey’s multiple comparison test. PWAB, post-weaning aortic banding; LVPWd, left ventricular posterior wall thickness at end-diastole; IVSd, interventricular septum thickness at end-diastole; LVIDd, left ventricular internal dimension at end-diastole; LVPWs, left ventricular posterior wall thickness at end-systole; IVSs, interventricular septum thickness at end-systole; LVIDs, left ventricular internal dimension at end-systole; LVM, left ventricular mass; LVMI, left ventricular mass index; RWT, relative wall thickness; FS, fractional shortening; EDV, end-diastolic volume; ESV, end-systolic volume; EF, ejection fraction

Supplemental Table 6. Physiological parameters and echocardiographic analysis post PWAB or sham procedure in control vs str*Nrip1*$^{-/-}$ female mice.

	Sham		PWAB	
	Control	str*Nrip1*$^{-/-}$	Control	str*Nrip1*$^{-/-}$
Body weight (g)	21.5 ± 0.6	20.5 ± 0.4	21.1 ± 0.2	21.5 ± 0.5
Biventricular weight (BV; mg)	89.5 ± 0.7	96.6 ± 3.4	129.1 ± 3.2 *	115.1 ± 5.0 #
Tibia length (mm)	16.7 ± 0.2	16.9 ± 0.1	16.6 ± 0.1	16.8 ± 0.1
BV / Tibia length (mg/mm)	5.34 ± 0.05	5.71 ± 0.17	7.79 ± 0.22 *	6.94 ± 0.28 #†
Heart rate (bpm)	586.8 ± 9.4	578.3 ± 14.7	576.1 ± 12.5	579.3 ± 13.6
LVPWd (mm)	0.724 ± 0.018	0.752 ± 0.030	1.037 ± 0.016 *	0.918 ± 0.041 #†
IVSd (mm)	0.724 ± 0.018	0.758 ± 0.031	1.045 ± 0.013 *	0.918 ± 0.041 #†
LVIDd (mm)	3.360 ± 0.087	3.448 ± 0.063	3.146 ± 0.129	3.327 ± 0.136
LVPWs (mm)	1.199 ± 0.036	1.128 ± 0.043	1.445 ± 0.058 *	1.334 ± 0.048 †
IVSs (mm)	1.199 ± 0.036	1.128 ± 0.043	1.440 ± 0.058 *	1.334 ± 0.048 †
LVIDs (mm)	1.896 ± 0.107	1.991 ± 0.084	1.736 ± 0.048	1.954 ± 0.088
LVM (mg)	75.9 ± 1.5	84.2 ± 4.3	116.3 ± 3.6 *	104.4 ± 3.4 †
LVMI (mg/g Body weight)	3.45 ± 0.10	3.89 ± 0.25	5.41 ± 0.14 *	4.73 ± 0.10 #†
RWT	0.43 ± 0.02	0.44 ± 0.03	0.66 ± 0.02 *	0.53 ± 0.03 †
FS (%)	43.66 ± 2.47	41.10 ± 2.31	44.86 ± 0.65	41.28 ± 0.87
Heart rate post zatebradine (bpm)	475.5 ± 12.4	481.2 ± 14.8	460.6 ± 11.8	462.7 ± 12.4
E wave (mm/sec)	644.7 ± 34.6	631.1 ± 55.0	740.7 ± 32.7	723.5 ± 26.0
A wave (mm/sec)	514.7 ± 24.3	494.2 ± 45.5	434.8 ± 42.9	561.3 ± 22.3
e' wave (mm/sec)	31.38 ± 2.23	30.16 ± 1.87	22.12 ± 1.27 *	27.25 ± 1.16
E/A	1.255 ± 0.050	1.305 ± 0.086	1.746 ± 0.108 *	1.296 ± 0.057 †
E/e'	20.99 ± 1.57	21.09 ± 2.61	34.28 ± 1.42 *	26.64 ± 0.72 †
EDV (μL)	37.7 ± 1.2	39.4 ± 1.2	31.7 ± 1.4 *	37.6 ± 1.1 †
ESV (μL)	10.4 ± 0.3	11.1 ± 0.4	9.3 ± 0.8	10.7 ± 0.8
EF (%)	72.4 ± 0.7	71.9 ± 0.9	70.8 ± 1.2	71.5 ± 1.7
Peak velocity (m/sec)	0.77 ± 0.07	0.84 ± 0.08	3.82 ± 0.06 *	3.88 ± 0.17 #

Echocardiography was performed on female mice 16 weeks post PWAB or sham surgery (n=6-7 per each group). E wave, A wave, e' wave evaluation were done after zatebradine treatment. Values are mean ± SEM. * $p < 0.05$ vs control-sham, # $p < 0.05$ vs strRIP140$^{-/-}$-sham, † $p < 0.05$ vs control-PWAB using 2-way ANOVA with Turkey's multiple comparison test. PWAB, post-weaning aortic banding; LVPWd, left ventricular posterior wall thickness at end-diastole; IVSd, interventricular septum thickness at end-diastole; LVIDd, left ventricular internal dimension at end-diastole; LVPWs, left ventricular posterior wall thickness at end-systole; IVSs, interventricular septum thickness at end-systole; LVIDs, left ventricular internal dimension at end-systole; LVM, left ventricular mass; LVMI, left ventricular mass index; RWT, relative wall thickness; FS, fractional shortening; EDV, end-diastolic volume; ESV, end-systolic volume; EF, ejection fraction

Supplemental Table 7. Physiological parameters and echocardiographic analysis in control vs cs*Nrip1*$^{-/-}$ mice.

	Control	cs*Nrip1*$^{-/-}$
Body weight (g)	20.1 ± 1.3	21.0 ± 0.8
Tibia length (mm)	16.3 ± 0.2	16.3 ± 0.1
Biventricular weight (BV) (mg)	89.0 ± 4.0	101.8 ± 2.2*
BV / Tibia length (mg/mm)	5.45 ± 0.19	6.26 ± 0.12*
Heart rate (bpm)	606.8 ± 11.2	617.7 ± 20.2
LVPWd (mm)	0.733 ± 0.018	0.878 ± 0.044*
IVSd (mm)	0.758 ± 0.014	0.881 ± 0.053*
LVIDd (mm)	3.258 ± 0.067	3.543 ± 0.116
LVPWs (mm)	1.142 ± 0.040	1.270 ± 0.073
IVSs (mm)	1.163 ± 0.030	1.263 ± 0.085
LVIDs (mm)	1.989 ± 0.066	2.173 ± 0.119
LVM (mg)	75.81 ± 4.5	110.2 ± 11.4*
LVMI (mg/g Body weight)	3.70 ± 0.14	4.67 ± 0.28*
RWT	0.46 ± 0.01	0.50 ± 0.03
FS (%)	38.91 ± 1.29	38.67 ± 0.42

Echocardiography was performed on 8 week-old male mice (n=6 per each group). Values are mean ± SEM. * $p < 0.05$ vs control using two-tailed, unpaired t-test. LVPWd, left ventricular posterior wall thickness at end-diastole; IVSd, interventricular septum thickness at end-diastole; LVIDd, left ventricular internal dimension at end-diastole; LVPWs, left ventricular posterior wall thickness at end-systole; IVSs, interventricular septum thickness at end-systole; LVIDs, left ventricular internal dimension at end-systole; LVM, left ventricular mass; LVMI, left ventricular mass index; RWT, relative wall thickness; FS, fractional shortening

Supplemental Table 8. Physiological parameters and echocardiographic analysis post TAC/MI or sham procedure in control vs cs*Nrip1*$^{-/-}$ mice.

	**Sham**		**TAC/MI**	
	Control	cs*Nrip1*$^{-/-}$	Control	cs*Nrip1*$^{-/-}$
Body weight (g)	22.8 ± 0.9	22.5 ± 1.0	23.5 ± .5	23.7 ± 0.6
Tibia length (mm)	18.4 ± 0.1	18.2 ± 0.2	18.3 ± 0.1	18.4 ± 0.1
Biventricular weight (BV) (mg)	100.4 ± 2.0	111.5 ± 4.7	171.3 ± 5.3 *	143.6 ± 6.5 # †
BV / Tibia length (mg/mm)	5.45 ± 0.11	6.12 ± 0.19	9.35 ± 0.28 *	7.82 ± 0.33 # †
Heart rate (bpm)	644.3 ± 7.3	636.0 ± 14.1	632.6 ± 14.6	631.6 ± 19.8
EDV (μL)	38.8 ± 3.0	36.1 ± 2.1	97.7 ± 10.0 *	70.6 ± 6.0 # †
ESV (μL)	10.9 ± 0.9	9.9 ± 0.7	71.4 ± 9.0 *	41.1 ± 5.4 # †
EF (%)	71.4 ± 1.0	72.4 ± 1.4	27.8 ± 2.3 *	43.5 ± 3.3 # †
Peak velocity (m/sec)	1.00 ± 0.05	1.11 ± 0.04	3.73 ± 0.41 *	3.75 ± 0.34 #

Echocardiography was performed 4 weeks post TAC/MI or sham surgery (n=6-8 per each group). Values are mean ± SEM. * $p < 0.05$ vs control-sham, # $p < 0.05$ vs cs*Nrip1*$^{-/-}$-sham, † $p < 0.05$ vs control-TAC/MI using 2-way ANOVA with Tukey's multiple comparison test. TAC, transverse aortic constriction; MI, spall apical myocardial infarction; EDV, end-diastolic volume; ESV, end-systolic volume; EF, ejection fraction

Supplemental Table 9. Pathways enriched in the RNA-seq post TAC/MI or sham procedure in control vs cs*Nrip1*$^{-/-}$ cardiac ventricle.

Pathway/Function Categories	Genes	Gene Count	Fold Enrichment	Adjusted *p*-value
**KEGG_PATHWAY enriched in genes protected from downregulation by RIP140 deficiency during TAC/MI**				
Valine, leucine and isoleucine degradation	Dbt, Acaa2, Hadha, Bckdhb, Acadm, Mccc2, Hadh, Aldh9a1, Ivd, Mccc1, Bcat2, Bckdha, Hadhb, Auh, Mcee	15	9.01	4.56E-09
Fatty acid elongation	Acot1, Acaa2, Hadha, Acot7, Hadh, Acot3, Hadhb	7	8.12	0.001702
Oxidative phosphorylation	Cox6a2, Cox8b, Ndufs6, Ndufa3, Cox7a1, Atp5f1, mt-Nd4, Cox7c, mt-Nd6, mt-Nd3, Atp5e, Atp5j, Cox7b, mt-Cytb, Cox6c, Ndufb4, Ndufb11, mt-Nd4l, mt-Atp8, mt-Co3, Ndufs4, mt-Atp6, Uqcrb, mt-Nd5, Ndufa5, Ndufv2, mt-Co1, Cox7a2, mt-Nd2, mt-Nd1	30	7.95	2.63E-17
Propanoate metabolism	Dbt, Hadha, Bckdhb, Ldhb, Sucla2, Bckdha, Mcee, Suclg2	8	7.92	0.000513
Cardiac muscle contraction	Hrc, Cox6a2, Myh6, Cox8b, Cox7a1, Tnni3, Cox7c, Cox7b, mt-Cytb, Cox6c, mt-Co3, Uqcrb, Trdn, mt-Co1, Cox7a2	15	6.08	1.67E-06
Fatty acid degradation	Acaa2, Hadha, Cpt2, Acadm, Hadh, Aldh9a1, Eci1, Hadhb	8	5.28	0.011616
Thermogenesis	Cox6a2, Cpt2, Cox8b, Ndufs6, Ndufa3, Cox7a1, Atp5f1, mt-Nd4, Cox7c, mt-Nd6, mt-Nd3, Atp5e, Prkab1, Atp5j, Cox7b, mt-Cytb, Cox6c, Ndufb4, Ndufb11, mt-Nd4l, Cox16, mt-Atp8, mt-Co3, Ndufs4, mt-Atp6, Uqcrb, mt-Nd5, Ndufa5, Ndufv2, mt-Co1, Cox7a2, mt-Nd2, mt-Nd1, Rps6ka2	34	5.18	1.26E-13
Non-alcoholic fatty liver disease	Bcl2l11, Cox6a2, Cox8b, Ndufs6, Ndufa3, Cox7a1, Mlxipl, Cox7c, Prkab1, Cox7b, mt-Cytb, Cox6c, Ndufb4, Ndufb11, mt-Co3, Ndufs4, Uqcrb, Ndufa5, Ndufv2, mt-Co1, Cox7a2	21	4.84	1.68E-07
Parkinson disease	Cox6a2, Camk2a, Cox8b, Ndufs6, Ndufa3, Cox7a1, Atp5f1, mt-Nd4, Cox7c, mt-Nd6, mt-Nd3, Atp5e, Atp5j, Cox7b, mt-Cytb, Cox6c, Ndufb4, Ndufb11, mt-Nd4l, mt-Atp8, mt-Co3, Ndufs4, mt-Atp6, Uqcrb, mt-Nd5, Hspa5, Ndufa5, Ndufv2, mt-Co1, Cox7a2, mt-Nd2, mt-Nd1	32	4.47	6.32E-11
Prion disease	Cox6a2, Cox8b, Ndufs6, Ndufa3, Cox7a1, Atp5f1, mt-Nd4, Cox7c, mt-Nd6, mt-Nd3, Atp5e, Atp5j, Cox7b, mt-Cytb, Cox6c, Ndufb4, Ndufb11, mt-Nd4l, mt-Atp8, mt-Co3, Ndufs4, mt-Atp6, Uqcrb, mt-Nd5, Hspa5, Ndufa5, Ndufv2, mt-Co1, Cox7a2, mt-Nd2, mt-Nd1	31	4	2.9E-09
Retrograde endocannabinoid signaling	Kcnj5, Kcnj3, Ndufs6, Ndufa3, Grm1, mt-Nd4, mt-Nd6, mt-Nd3, Ndufb4, Ndufb11, mt-Nd4l, Ndufs4, mt-Nd5, Ndufa5, Ndufv2, mt-Nd2, mt-Nd1	17	4	0.000104
Metabolic pathways	Glo1, Sord, Selenbp1, Gstk1, Acot1, Dbt, Pfkfb1, Acaa2, Cox6a2, Fbp2, Hadha, Kyat3, Gstm7, Bckdhb, Dglucy, Ldhd, Cox8b, Ndufs6, Ndufa3, Cox7a1, Acadm, Atp5f1, Mccc2, Ldhb, Oplah, Adi1, Dgat2, Nampt, Gpt2, Hadh, Aldh9a1, Amy1, Gstt1, mt-Nd4, Ivd, Cad, Idh1, Chkb, Dguok, Cox7c, mt-Nd6, Mccc1, Acot3, mt-Nd3, Atp5e, Gpt, Bcat2, Cmbl, Atp5j, Cox7b, mt-Cytb, Fdft1, Sqor, Alad, Cox6c, Sucla2, Bckdha, Dut, Hadhb, Akr1b10, Pank1, Ndufb4, Ndufb11, mt-Nd4l, mt-Atp8, Galm, Gstm5, mt-Co3, Ndufs4, Gpam, mt-Atp6, Auh, Uqcrb, mt-Nd5, Ppat, Pla2g2d, Acp1, Ndufa5, Ndufv2, mt-Co1, Pla2g4e, Cox7a2, mt-Nd2, Mmab, mt-Nd1, Mcee, Pmvk, Pigyl, Entpd5, Dgke, Dot1l, Gstt2, Sds, Suclg2, Hnmt, Nnt, Chac1, Acsm5, Acyp2	99	2.17	9.08E-14

Pathway/Function Categories	Genes	Gene Count	Fold Enrichment	Adjusted *p*-value
**GO_Biological Process enriched in genes protected from downregulation by RIP140 deficiency during TAC/MI**				
electron transport coupled proton transport	mt-Nd4, mt-Cytb, mt-Nd5, mt-Co1	4	30.54	0.006331
positive regulation of heart contraction	Adra1a, Smtn, Atp5j, Trpm4, Adrb1, Rnf207, Apln, Crhr2, Strit1	9	7.47	0.006981
fatty acid beta-oxidation	Etfb, Acaa2, Hadha, Etfa, Cpt2, Decr1, Acadm, Hadh, Ivd, Eci1, Hadhb, Echdc2	12	7.16	0.000242
glutathione metabolic process	Glo1, Gstk1, Gstm7, Oplah, Gstt1, Idh1, Gstm5, Hmgn5, Gstt2, Chac1	10	6.7	0.005526
cardiac conduction	Pln, Kcnj5, Kcnj2, Tmem65, Trpm4, Tnni3k, Scn10a, Abcc9, Scn4b, Sptbn4	10	6.47	0.007672
regulation of cardiac muscle contraction	Pln, Kcnj2, Adra1a, Tnni3, Smtn, Trpm4, Tnni3k, Scn10a, Adrb1, Rnf207, Hdac4, Strit1	12	6.36	0.000937
brown fat cell differentiation	Selenbp1, Slc2a4, Lamb3, Nudt7, Flcn, Fndc5, Itga6, Adrb1, Dusp10	9	6.25	0.032692
acyl-CoA metabolic process	Acot1, Acaa2, Acot7, Ces1d, Dgat2, Acot3, Nudt7, Sucla2, Gpam, Mcee, Pmvk, Suclg2, Acsm5	13	5.98	0.000646
oxidative phosphorylation	Cox6a2, Ndufs6, Cox7a1, Atp5f1, Mlxipl, Dguok, Cox7c, Atp5j, Msh2, mt-Co3, Slc25a33, mt-Atp6, Ndufv2, mt-Co1, Cox7a2	15	5.96	7.88E-05
fatty acid catabolic process	Etfb, Acaa2, Hadha, Acot7, Etfa, Cpt2, Decr1, Ces1d, Acadm, Hadh, Ivd, Eci1, Hadhb, Echdc2	14	5.75	0.00037
cardiac muscle contraction	Pln, Kcnj5, Myh6, Kcnj2, Adra1a, Tnni3, Smtn, Map2k6, Trpm4, Tcap, Tnni3k, Scn10a, Tnnt1, Adrb1, Rnf207, Scn4b, Hdac4, Strit1	18	5.63	8.36E-06
heart contraction	Hrc, Pln, Kcnj5, Myh6, Kcnj2, Hopx, Adra1a, Tnni3, Tmem65, Smtn, Atp5j, Map2k6, Trpm4, Tcap, Tnni3k, Scn10a, Thrb, Tnnt1, Trdn, Abcc9, Adrb1, Rnf207, Scn4b, Apln, Sptbn4, Crhr2, Hdac4, Rps6ka2, Strit1	29	5.22	8.69E-10
regulation of heart rate	Kcnj5, Myh6, Kcnj2, Adra1a, Atp5j, Trpm4, Tnni3k, Scn10a, Adrb1, Scn4b, Sptbn4, Crhr2	12	5.09	0.010899
heart process	Hrc, Pln, Kcnj5, Myh6, Kcnj2, Hopx, Adra1a, Tnni3, Tmem65, Smtn, Atp5j, Map2k6, Trpm4, Tcap, Tnni3k, Scn10a, Thrb, Tnnt1, Trdn, Abcc9, Adrb1, Rnf207, Scn4b, Apln, Sptbn4, Crhr2, Hdac4, Rps6ka2, Strit1	29	4.99	2.85E-09
striated muscle contraction	Pln, Kcnj5, Myh6, Kcnj2, Adra1a, Grcc10, Tnni3, Lmod3, Smtn, Map2k6, Trpm4, Tcap, Tnni3k, Scn10a, Tnnt1, Adrb1, Rnf207, Scn4b, Hdac4, Strit1	20	4.99	9.72E-06
sulfur compound metabolic process	Glo1, Gstk1, Acot1, Acaa2, Acot7, Gstm7, Egflam, Ces1d, Oplah, Adi1, Stat5a, Dgat2, Gstt1, Idh1, Acot3, Nudt7, Sqor, Sucla2, Gstm5, Hs3st5, Gpam, Ghr, Mcee, Pmvk, Hmgn5, Gstt2, Suclg2, Chac1, Acsm5	29	4.19	2.15E-07
regulation of blood circulation	Hrc, Pln, Kcnj5, Myh6, Kcnj2, Hopx, Adra1a, Tnni3, Tmem65, Smtn, Atp5j, Trpm4, Tnni3k, Scn10a, Thrb, Abcc9, Adrb1, Rnf207, Scn4b, Apln, Sptbn4, Crhr2, Agtr1a, Hdac4, Strit1	25	4.08	7.92E-06
carboxylic acid catabolic process	Etfb, Adhfe1, Acaa2, Hadha, Acot7, Etfa, Bckdhb, Cpt2, Ldhd, Decr1, Ces1d, Acadm, Mccc2, Hadh, Ivd, Eci1, Bcat2, Hadhb, Echdc2, Ppat, Sds	21	4.01	0.0002
cellular respiration	Slc25a22, Cox6a2, Ndufs6, mt-Nd4, Dguok, Cox7c, Flcn, Sucla2, Coq10a, mt-Co3, Ndufa5, Ndufv2, mt-Co1, mt-Nd1, Suclg2	15	3.84	0.024733
ATP metabolic process	Cox6a2, Myh6, Ldhd, Ndufs6, Cox7a1, Atp5f1, Mlxipl, Dguok, Cox7c, Atp5e, Atp5j, Flcn, Msh2, mt-Co3, Slc25a33, mt-Atp6, Ndufv2, mt-Co1, Cox7a2, Entpd5, Hdac4	21	3.56	0.001466
ribose phosphate metabolic process	Acot1, Acaa2, Acot7, Ldhd, Ces1d, Atp5f1, Mccc2, Dgat2, Mlxipl, Cad, Dguok, Acot3, Atp5e, Nudt7, Atp5j, Flcn, Sucla2, Pank1, Gpam, mt-Atp6, Ppat, Mcee, Pmvk, Entpd5, Suclg2, Hdac4, Acsm5	27	3.31	0.000159
small molecule catabolic process	Glo1, Sord, Etfb, Adhfe1, Acaa2, Hadha, Acot7, Etfa, Bckdhb, Cpt2, Ldhd, Decr1, Ces1d, Acadm, Mccc2, Hadh, Ivd, Eci1, Bcat2, Hadhb, Echdc2, Galm, Ppat, Sds	24	3.18	0.001838
generation of precursor metabolites and energy	Slc25a22, Cox6a2, Ndufs6, Cox7a1, Acadm, Atp5f1, Mlxipl, mt-Nd4, Dguok, Cox7c, Atp5j, Flcn, Sucla2, Msh2, Coq10a, mt-Co3, Slc25a33, mt-Atp6, Ndufa5, Ndufv2, mt-Co1, Cox7a2, Adrb1, mt-Nd1, Entpd5, Suclg2, Hdac4, Phkg1	28	2.96	0.000948
circulatory system process	Hrc, Pln, P2ry1, Kcnj5, Myh6, Kcnj2, Hopx, Slc27a1, Adra1a, Tnni3, Slc22a5, Nampt, Tmem65, Smtn, Atp5j, Slc5a6, Ncald, Map2k6, Trpm4, Tcap, Tnni3k, Scn10a, Thrb, Tnnt1, Trdn, Abcc9, Adrb1, Rnf207, Scn4b, Mkks, Apln, Sptbn4, Crhr2, Agtr1a, Hdac4, Rps6ka2, C2cd4b, Strit1	38	2.95	7.72E-06
purine-containing compound metabolic process	Acot1, Acaa2, Acot7, Ldhd, Ces1d, Atp5f1, Mccc2, Dgat2, Mlxipl, Dguok, Acot3, Atp5e, Nudt7, Atp5j, Flcn, Sucla2, Pank1, Gpam, mt-Atp6, Nudt15, Mcee, Pmvk, Entpd5, Slc16a9, Suclg2, Hdac4, Acsm5	27	2.88	0.002657
organophosphate metabolic process	Acot1, P2ry1, Pfkfb1, Acaa2, Fbp2, Hadha, Slc27a1, Acot7, Ldhd, Ces1d, Atp5f1, Mccc2, Ttc7, Dgat2, Nampt, Fitm1, Mlxipl, Cad, Idh1, Chkb, Dguok, Acot3, Atp5e, Nudt7, Atp5j, Fdft1, Flcn, Sucla2, Dut, Erbb4, Pank1, Gpcpd1, Gpd2, Pitpnc1, Gpam, mt-Atp6, Ppat, Pla2g2d, Pla2g4e, Nudt15, Mcee, Pmvk, Pigyl, Enpp2, Entpd5, Dgke, Enpp5, Suclg2, Hdac4, Acsm5, Pik3ip1, Angptl3, Hdhd5	53	2.64	3.29E-07
monovalent inorganic cation transport	Kcnv2, Kcnd2, Kcnj5, Kcnip2, Kcnj2, Slc36a2, Kcnj3, Slc9a2, Atp5f1, mt-Nd4, Atp5e, Atp5j, mt-Cytb, Slc5a6, Tesc, Trpm4, Akap5, Scn10a, mt-Atp6, mt-Nd5, mt-Co1, Abcc9, Rnf207, Scn4b, Klhl3, Sptbn4	26	2.6	0.027169
muscle system process	Pln, Kcnj5, Myh6, Kcnj2, Adra1a, Ndufs6, Grcc10, Tnni3, Lmod3, Trip10, Smtn, Fbxo32, Map2k6, Tmod4, Trpm4, Tcap, Tnni3k, Scn10a, Tnnt1, Adrb1, Rnf207, Scn4b, Clcn1, Mkks, Hdac4, Strit1	26	2.55	0.037358
monocarboxylic acid metabolic process	Glo1, Etfb, Acot1, Ucp3, Acaa2, Hadha, Slc27a1, Acot7, Etfa, Gstm7, Cpt2, Ldhd, Decr1, Ndufs6, Ces1d, Npc1, Acadm, Ldhb, Dgat2, Hadh, Mlxipl, Ivd, Eci1, Acot3, Hadhb, Echdc2, Gpam, As3mt, Echdc3, Insig1, Entpd5, Prox1, Sds, Hdac4, Acsm5, Angptl3, Alkbh7	37	2.54	0.000651
oxidation-reduction process	Slc25a22, Etfb, Acaa2, Cox6a2, Hadha, Etfa, Cpt2, Decr1, Ndufs6, Acadm, Dgat2, Nqo2, Hadh, mt-Nd4, Ivd, Dguok, Cox7c, Eci1, Sqor, Flcn, Sucla2, Hadhb, Echdc2, Coq10a, mt-Co3, Ndufa5, Ndufv2, mt-Co1, Adrb1, mt-Nd1, Suclg2, Phkg1	32	2.39	0.014953
regulation of system process	Hrc, Pln, Kcnj5, Myh6, Kcnj2, Hopx, Adra1a, Grcc10, Tnni3, Slc22a5, Tmem65, Grm1, Trip10, Smtn, Atp5j, Fbxo32, Cry2, Trpm4, Tnni3k, Tenm4, Tppp, Scn10a, Thrb, Zfp488, Tnnt1, Abcc9, Adrb1, Rnf207, Scn4b, Apln, Sptbn4, Crhr2, Agtr1a, Hdac4, Strit1	35	2.28	0.014854

Supplemental Table 9. Continued

Pathway/Function Categories	Genes	Gene Count	Fold Enrichment	Adjusted *p*-value
**KEGG_PATHWAY enriched in genes protected from upregulation by RIP140 deficiency during TAC/MI**				
ECM-receptor interaction	Thbs4, Comp, Col1a2, Thbs1, Tnc, Col4a2, Col1a1, Itga9, Itga5, Col4a1, Itgb5, Fn1, Col6a1, Col6a2, Col4a3, Lamc1, Itga11, Col2a1, Col4a5, Col4a4, Col9a2, Itgb3, Cd44	23	6.69	1.64E-11
Protein digestion and absorption	Col8a1, Col5a2, Col1a2, Col4a2, Col1a1, Col14a1, Col4a1, Col16a1, Col3a1, Col6a1, Col5a1, Col6a2, Slc38a2, Col4a3, Col2a1, Eln, Col4a5, Col4a4, Col9a2, Col5a3, Col18a1, Slc3a2	22	5.2	1.25E-08
Amoebiasis	Col1a2, Hspb1, Col4a2, Col1a1, Tgfb2, Tgfb3, Col4a1, Fn1, Tlr4, Col3a1, Col4a3, Lamc1, Col4a5, Col4a4, Actn4, Actn1, Arg1, Itgam, Plcb4	19	4.67	1.58E-06
Focal adhesion	Thbs4, Comp, Col1a2, Thbs1, Tnc, Col4a2, Col1a1, Itga9, Itga5, Col4a1, Itgb5, Fn1, Flnc, Zyx, Rock2, Col6a1, Col6a2, Flt4, Col4a3, Lamc1, Rapgef1, Itga11, Col2a1, Ccnd2, Col4a5, Col4a4, Col9a2, Itgb3, Tln1, Actn4, Shc1, Actn1, Pip5k1b, Pdgfc, Cav3, Parvb	36	4.65	4.81E-13
Chronic myeloid leukemia	Tgfb2, Tgfb3, Tgfbr1, Gadd45g, Gab2, Bcl2l1, Runx1, Ptpn11, E2f3, Nfkbia, Cdkn1a, Bak1, Shc1	13	4.39	0.000637
AGE-RAGE signaling pathway in diabetic complications	Mmp2, Col1a2, Col4a2, Col1a1, Tgfb2, Tgfb3, Col4a1, Fn1, Col3a1, Tgfbr1, Plcg2, Col4a3, Plcd3, Col4a5, Serpine1, Col4a4, Plcb4	17	4.35	2.73E-05
Small cell lung cancer	Col4a2, Col4a1, Fn1, Gadd45g, Col4a3, Lamc1, Bcl2l1, Col4a5, Col4a4, E2f3, Nfkbia, Cdkn1a, Bak1, Traf2	14	3.98	0.00091
Hypertrophic cardiomyopathy	Myh7, Ace, Tgfb2, Tgfb3, Itga9, Itga5, Itgb5, Itga11, Itgb3, Lmna, Dmd, Prkab2, Des	13	3.74	0.003799
Complement and coagulation cascades	C4b, Cfh, Serping1, C1ra, F2r, Clu, Masp1, C1s1, Serpine1, Plat, C3, Itgam, C7	13	3.62	0.005452
Leishmaniasis	Tgfb2, Tgfb3, Tlr4, Fcgr3, Ifngr1, Irak4, C3, Nfkbia, Ncf1, Itgam	10	3.62	0.039655
Relaxin signaling pathway	Mmp2, Col1a2, Adcy7, Ednrb, Col4a2, Col1a1, Col4a1, Gnao1, Col3a1, Tgfbr1, Col4a3, Atf4, Col4a5, Col4a4, Nfkbia, Shc1, Plcb4, Gng4	18	3.59	0.000228
Dilated cardiomyopathy	Myh7, Adcy7, Tgfb2, Tgfb3, Itga9, Itga5, Itgb5, Itga11, Itgb3, Lmna, Dmd, Des	12	3.34	0.022982
HIF-1 signaling pathway	Nppa, Timp1, Tlr4, Plcg2, Pfkp, Ifngr1, Serpine1, Slc2a1, Hmox1, Il6ra, Cdkn1a, Ltbr, Eif4ebp1, Egln3	14	3.22	0.010344
PI3K-Akt signaling pathway	Thbs4, Comp, Col1a2, Nr4a1, Thbs1, Tnc, Col4a2, Col1a1, Itga9, Itga5, Col4a1, Itgb5, Fn1, Tlr4, Col6a1, F2r, Col6a2, Flt4, Osmr, Col4a3, Lamc1, Atf4, Bcl2l1, Itga11, Col2a1, Ccnd2, Col4a5, Col4a4, Pkn3, Col9a2, Itgb3, Il4ra, Sgk1, Fgfr1, Il6ra, Cdkn1a, Pkn1, Fgfr3, Pdgfc, Eif4ebp1, Ddit4, Fgf6, Gng4	43	3.07	2.76E-09
Proteoglycans in cancer	Mmp2, Hbegf, Col1a2, Thbs1, Col1a1, Tgfb2, Itga5, Itgb5, Fn1, Tlr4, Flnc, Rock2, Plcg2, Fzd1, Ptpn11, Arhgef12, Itgb3, Cd44, Fgfr1, Cdkn1a, Iqgap1, Msn, Cav3	23	2.97	0.000264
Osteoclast differentiation	Ctsk, Tgfb2, Tgfbr1, Tnfrsf1a, Plcg2, Fcgr3, Gab2, Ifngr1, Fosl2, Itgb3, Fcgr2b, Nfkbia, Ncf1, Traf2	14	2.95	0.026384
MicroRNAs in cancer	Thbs1, Tnc, Tgfb2, Itga5, Plcg2, Cyp1b1, Ccnd2, E2f3, Itgb3, Cd44, Hmox1, Cdkn1a, Bak1, Shc1, Slc45a3, Fgfr3, Reck, Ddit4	18	2.85	0.005812
Regulation of actin cytoskeleton	Enah, Itga9, Itga5, Itgb5, Fn1, Arpc1b, Rock2, F2r, Itga11, Fgd3, Gna13, Arhgef12, Itgb3, Fgfr1, Myh10, Actn4, Iqgap1, Actn1, Pip5k1b, Fgfr3, Msn, Pdgfc, Fgf6, Itgam	24	2.84	0.000358
Phagosome	Thbs4, Comp, Tubb2a, Thbs1, Itga5, Itgb5, Tlr4, C1ra, Fcgr3, Msr1, Ctss, Itgb3, Fcgr2b, C3, Dync1li1, Ncf1, Atp6v1b2, Itgam	18	2.63	0.016046
Human papillomavirus infection	Thbs4, Comp, Col1a2, Thbs1, Tnc, Col4a2, Col1a1, Itga9, Itga5, Col4a1, Itgb5, Fn1, Col6a1, Tnfrsf1a, Col6a2, Col4a3, Lamc1, Hes1, Fzd1, Itga11, Col2a1, Ccnd2, Col4a5, Col4a4, Col9a2, Itgb3, Lfng, Cdkn1a, Bak1, Eif4ebp1, Atp6v1b2	31	2.26	0.001658
Pathways in cancer	Mmp2, Adcy7, Ednrb, Col4a2, Tgfb2, Tgfb3, Col4a1, Fn1, Rock2, F2r, Tgfbr1, Gadd45g, Flt4, Plcg2, Col4a3, Lamc1, Hes1, Ifngr1, Fzd1, Bcl2l1, Runx1, Ccnd2, Col4a5, Col4a4, E2f3, Gna13, Arhgef12, Il4ra, Slc2a1, Fgfr1, Hmox1, Il6ra, Nfkbia, Cdkn1a, Bak1, Fgfr3, Egln3, Fgf6, Ccdc6, Plcb4, Traf2, Gng4	42	2.01	0.00099

Pathway/Function Categories	Genes	Gene Count	Fold Enrichment	Adjusted *p*-value
**GO_Biological Process enriched in genes protected from upregulation by RIP140 deficiency during TAC/MI**				
elastic fiber assembly	Mfap4, Lox, Fbln5, Emilin1, Ltbp3, Atp7a	6	17.63	0.000675
glomerular basement membrane development	Nid1, Sulf2, Col4a3, Sulf1, Col4a4	5	16.33	0.012024
collagen fibril organization	Comp, Lox, Col5a2, Fmod, Col1a2, Col1a1, Col14a1, Tgfb2, Scx, Adamts2, Loxl2, Col3a1, Loxl3, Col5a1, Tgfbr1, P4ha1, Aebp1, Emilin1, Cyp1b1, Col2a1, Atp7a	21	11.64	2.91E−14
response to muscle stretch	Ankrd1, Ankrd23, Csrp3, Nfkbia, Dmd, Cav3	6	10.37	0.032404
negative regulation of cartilage development	Frzb, Ctsk, Tgfb2, Tgfbr1, Ptpn11, Ccn4, Ltbp3, Adamts12	8	8.4	0.00696
type B pancreatic cell proliferation	Igfbp3, Nr4a1, Sfrp1, Nupr1, Errfi1, Sidt2, Igfbp5	7	8.23	0.034245
extracellular matrix assembly	Mfap4, Lox, Col1a2, Qsox1, Antxr1, Fbln5, Emilin1, Smpd3, Ltbp3, Atp7a, Rgcc	11	8.08	0.000146
trabecula formation	Mmp2, Adamts1, Col1a1, Sfrp1, Bmp10, Fbn2, Cav3	7	7.91	0.045492
platelet-derived growth factor receptor signaling pathway	Lox, Apod, Hip1, Csrnp1, Rapgef1, Ptpn11, Plat, Ptpn1, Itgb3, Smpd3, Iqgap1, Ndrg4, Ptpn2, Pdgfc	14	7.35	8.93E−06
vascular endothelial growth factor production	Adgrg1, Sulf2, Flt4, Sulf1, Atf4, Cyp1b1, Il6ra, C3, Sars	9	7.35	0.005685
face morphogenesis	Mmp2, Crispld2, Col1a1, Tgfb2, Scx, Tgfb3, Csrnp1, Ptpn11	8	7.12	0.02675
positive regulation of protein tyrosine kinase activity	Lrp8, Hbegf, Ace, App, Gprc5b, Prnp, Nedd9, Dok7, Ptpn1, Ncf1	10	7	0.002538
extracellular matrix organization	Postn, Comp, Col8a1, Mmp2, Mfap4, Lox, Col5a2, Crispld2, Adamtsl2, Fmod, Col1a2, Ccn2, Adamts1, Col4a2, Col1a1, Col14a1, Tgfb2, Scx, Col4a1, Adamts2, Nid1, Col16a1, Fn1, Loxl2, Col3a1, Qsox1, Spock2, Loxl3, Sulf2, Col5a1, Antxr1, Angptl7, Tgfbr1, P4ha1, Aebp1, Tnfrsf1a, App, Adamts9, Mmp14, Fbln5, Col4a3, Sulf1, Ctss, Emilin1, B4galt1, Cyp1b1, Col2a1, Eln, Col4a5, Col4a4, Itgb3, Smpd3, Adamts15, Ltbp3, Atp7a, Tgfbi, Colq, Col5a3, Reck, Col18a1, Ccdc80, Adamts12, Rgcc, Adamts4, Sh3pxd2b	65	6.95	6.04E−33
trabecula morphogenesis	Mmp2, Adamts1, Col1a1, Tgfb2, S1pr1, Sfrp1, Sbno2, Tgfbr1, Bmp10, Fbn2, Cav3	11	6.6	0.001388
collagen biosynthetic process	Ccn2, Col1a1, Scx, Tgfb3, Fn1, Col5a1, Rcn3, F2r, Emilin1, Il6ra, Errfi1, Arg1, Rgcc	13	6.59	0.000133
neuromuscular junction development	Sorbs2, Tnc, Col4a1, F2r, Etv5, App, Pdzrn3, Snta1, Dok7, Col4a5, Colq, Ppfibp1	12	6.53	0.000479
collagen metabolic process	Mmp2, Mfap4, Col1a2, Ccn2, P3h2, Ctsk, Col1a1, Scx, Tgfb3, Fn1, Col5a1, Rcn3, F2r, Mmp14, Ctss, Emilin1, Smpd3, Il6ra, Errfi1, Id1, Arg1, Rgcc	22	6.34	7.77E−09
negative regulation of smooth muscle cell proliferation	Nppb, Igfbp3, Apod, Comt, Klf4, Ctnnbip1, Hmox1, Cdkn1a, Ndrg4, Npr3, Igfbp5	11	5.99	0.003907
integrin-mediated signaling pathway	Itgbl1, Ccn2, Timp1, Itga5, Fn1, Col3a1, Zyx, Loxl3, Ptk2b, Ptpn11, Pcsk5, Adam15, Itgb3, Tln1, Sema7a	15	5.96	5.44E−05
regulation of cartilage development	Frzb, Ccn2, Ctsk, Tgfb2, Scx, Loxl2, Tgfbr1, Gdf6, Bmp10, Ptpn11, Smpd3, Ccn4, Ltbp3, Adamts12	14	5.88	0.000192
cellular response to acid chemical	Mmp2, Col5a2, Col1a2, Col1a1, Col4a1, Col16a1, Pkd2, Col3a1, Col6a1, Bcl2l1, Atp7a, Pdgfc, Castor1	13	5.7	0.000808
regulation of protein tyrosine kinase activity	Lrp8, Hbegf, Ace, App, Gprc5b, Prnp, Nedd9, Dok7, Ptpn1, Itgb3, Errfi1, Ncf1, Ptpn2	13	5.54	0.001157
regulation of collagen metabolic process	Mfap4, Ccn2, Scx, Tgfb3, Fn1, F2r, Emilin1, Il6ra, Errfi1, Rgcc	10	5.44	0.028566

Supplemental Table 9. Continued

Pathway/Function Categories	Genes	Gene Count	Fold Enrichment	Adjusted *p*-value
**GO_Biological Process enriched in genes protected from upregulation by RIP140 deficiency during TAC/MI**				
glomerulus development	Cfh, Nid1, Sulf2, Wwtr1, Col4a3, Sulf1, Hes1, Col4a4, Itgb3, Il6ra, Iqgap1	11	5.39	0.011623
tumor necrosis factor-mediated signaling pathway	Otulin, Tnfrsf1a, Ptk2b, Krt18, Krt8, Sphk1, Hipk1, Nfkbia, Actn4, Ptpn2, Traf2	11	5.3	0.01375
positive regulation of smooth muscle cell migration	Postn, Adamts1, Cyp1b1, Pcsk5, Itgb3, Ccn4, Atp7a, Mif, Iqgap1, Igfbp5	10	5.25	0.039981
chondrocyte differentiation	Comp, Ccn2, Scx, Loxl2, Sulf2, Tgfbr1, Gdf6, Sulf1, Ptpn11, Col2a1, Smpd3, Ccn4, Ltbp3, Ecm1, Runx3, Fgfr1, Arid5a, Adamts12	18	5.24	1.88E−05
myofibril assembly	Synpo2l, Ankrd23, Acta1, Csrp3, Bmp10, Nrap, Prkar1a, Krt8, Myh10, Cav3, Wdr1	11	5.21	0.016208
cartilage development	Comp, Frzb, Ccn2, Ctsk, Col1a1, Tgfb2, Stc1, Scx, Loxl2, Sulf2, Tgfbr1, Hand2, Gdf6, Sulf1, Bmp10, Ptpn11, Col2a1, Snai1, Smpd3, Ccn4, Ltbp3, Atp7a, Ecm1, Runx3, Fgfr1, Trpv4, Pkd1, Arid5a, Fgf6, Satb2, Adamts12	31	5.18	1.04E−10
muscle fiber development	Comp, Acta1, Lox, Rcan1, Uchl1, Gpx1, Flnc, Myof, Nrap, Dmd	10	5.16	0.047026
striated muscle cell development	Comp, Synpo2l, Ankrd23, Acta1, Lox, Sorbs2, Pi16, Rcan1, Lrrc10, Csrp3, Col14a1, Uchl1, Gpx1, Flnc, Myof, Pdlim5, Bmp10, Popdc2, Nrap, Prkar1a, Ccn4, Krt8, Lmna, Dmd, Myh10, Cav3, Wdr1	27	4.81	2.77E−08
smooth muscle cell migration	Postn, Igfbp3, Ace, Adamts1, Cyp1b1, Pcsk5, Plat, Itgb3, Ccn4, Atp7a, Mif, Iqgap1, Ndrg4, Igfbp5	14	4.78	0.002724
muscle cell migration	Thbs4, Postn, Igfbp3, Ace, Plekho1, Adamts1, Cyp1b1, Pcsk5, Plat, Itgb3, Ccn4, Atp7a, Mif, Iqgap1, Ndrg4, Igfbp5	16	4.75	0.000523
positive regulation of cell-substrate adhesion	Col8a1, Dbn1, Fbln2, Itga5, Nid1, Col16a1, Fn1, Spock2, Ptk2b, Nedd9, Emilin1, Pcsk5, Ninj1, Itgb3, Dmd, Disc1, Iqgap1, Plekha2, Ccdc80	19	4.73	4.12E−05
platelet activation	Comp, Cfh, Fn1, Tlr4, F2r, Gna13, Itgb3, Serpine2, Entpd2, C1qtnf1, Entpd1, Mertk	12	4.7	0.019014
response to acid chemical	Mmp2, Col5a2, Col1a2, Col1a1, Col4a1, Col16a1, Pkd2, Col3a1, Col6a1, Bcl2l1, Krt8, Atp7a, Pdgfc, Castor1	14	4.62	0.00417
regulation of epithelial to mesenchymal transition	Col1a1, Tgfb2, Tgfb3, Loxl2, Sfrp1, Tgfbr1, Wwtr1, Spry1, Snai1, Tbx20, Usf3, Dact3, Rgcc, Il17rd	14	4.62	0.00417
muscle cell development	Comp, Synpo2l, Ankrd23, Acta1, Lox, Sorbs2, Pi16, Rcan1, Lrrc10, Csrp3, Col14a1, Uchl1, Gpx1, Flnc, Myof, Pdlim5, Bmp10, Hes1, Popdc2, Nrap, Prkar1a, Ccn4, Krt8, Lmna, Dmd, Myh10, Cav3, Wdr1	28	4.57	4.03E−08
response to amino acid	Mmp2, Col5a2, Col1a2, Col1a1, Col4a1, Col16a1, Col3a1, Col6a1, Bcl2l1, Atp7a, Pdgfc, Castor1	12	4.52	0.028804
striated muscle hypertrophy	Nppa, Nppb, Sorbs2, Myh7, Pi16, Csrp3, Col14a1, Pdlim5, Hand2, Tnfrsf1a, Bmp10, Lmcd1, Ccn4, Lmna, Errfi1, Cav3, Igfbp5	17	4.46	0.000561
positive regulation of endothelial cell migration	Sparc, Hspb1, Flt4, Srpx2, Amot, Ptk2b, Amotl1, Itgb3, Fgfr1, Adgra2, Hmox1, Atoh8, Rhoj, Grn	14	4.42	0.00715
actin filament bundle assembly	Synpo2l, Ccn2, Pdlim1, Dpysl3, Fam107a, Dbn1, S1pr1, Rhoc, Zyx, Tgfbr1, Ppm1e, Ptk2b, Sorbs3, Ppfia1, Eln, Cald1, Myh10, Actn4, Id1, Actn1, Eps8, Rgcc, Sh3pxd2b	23	4.39	5.91E−06
cardiac muscle cell development	Sorbs2, Pi16, Lrrc10, Csrp3, Col14a1, Pdlim5, Bmp10, Popdc2, Nrap, Ccn4, Lmna, Myh10, Cav3	13	4.39	0.017474
negative regulation of cellular response to growth factor stimulus	Aspn, Adamtsl2, Fbn1, Sfrp1, Rasl11b, Sulf2, Sulf1, Fstl3, Emilin1, Fzd1, Spry1, Nbl1, Ptpn1, Skil, Adgra2, Htra3, Hipk2, Ptprf, Fbn2, Adamts12, Il17rd	21	4.38	3.07E−05
actin filament bundle organization	Synpo2l, Ccn2, Pdlim1, Dpysl3, Fam107a, Dbn1, S1pr1, Rhoc, Zyx, Tgfbr1, Ppm1e, Ptk2b, Sorbs3, Ppfia1, Eln, Cald1, Myh10, Actn4, Id1, Actn1, Eps8, Rgcc, Sh3pxd2b	23	4.31	8.67E−06
response to transforming growth factor beta	Ankrd1, Lox, Aspn, Adamtsl2, Col1a2, Col4a2, Dbn1, Tgfb2, Scx, Tgfb3, Ltbp4, Fbn1, Col3a1, Zyx, Rasl11b, Tgfbr1, Emilin1, Spry1, Skil, Ltbp3, Runx3, Htra3, Hipk2, Adam9, Fbn2, Cav3, Il17rd	27	4.27	4.69E−07
regulation of smooth muscle cell proliferation	Mmp2, Nppb, Igfbp3, Hbegf, Adamts1, Apod, S1pr1, Sulf1, Comt, Klf4, Itgb3, Smpd3, Ccn4, Tgm2, Irak4, Ctnnbip1, Hmox1, Il6ra, Cdkn1a, Shc1, Ndrg4, Npr3, Igfbp5	23	4.25	1.11E−05
transforming growth factor beta receptor signaling pathway	Lox, Aspn, Adamtsl2, Col1a2, Tgfb2, Tgfb3, Ltbp4, Fbn1, Col3a1, Zyx, Rasl11b, Tgfbr1, Emilin1, Spry1, Skil, Ltbp3, Htra3, Hipk2, Adam9, Fbn2, Cav3, Il17rd	22	4.23	2.69E−05
response to BMP	Comp, Gdf15, Fstl1, Tgfb2, Scx, Fbn1, Sfrp1, Gdf6, Sulf1, Bmp10, Hes1, Fstl3, Fzd1, Col2a1, Nbl1, Smpd3, Skil, Htra3, Hipk2, Id1, Adamts12, Zfp423	22	4.17	3.43E−05
positive regulation of epithelial cell migration	Rtn4, Sparc, Hbegf, Hspb1, Tgfb2, Flt4, Srpx2, Plcg2, Amot, Ptk2b, Amotl1, Itgb3, Fgfr1, Adgra2, Hmox1, Adam9, Atoh8, Rhoj, Grn	19	4.17	0.000336
intrinsic apoptotic signaling pathway in response to DNA damage	Phlda3, Nupr1, Tnfrsf1a, Clu, Bcl2l1, Snai1, Skil, Hipk1, Plscr1, Hmox1, Cdkn1a, Mif, Hipk2, Bak1, Ddit4	15	4.16	0.007074
actomyosin structure organization	Synpo2l, Ankrd23, Acta1, Ccn2, Pdlim1, Csrp3, S1pr1, Rhoc, Zyx, Tgfbr1, Ppm1e, Anln, Bmp10, Ptk2b, Sorbs3, Ppfia1, Eln, Nrap, Prkar1a, Krt8, Myh10, Cav3, Rgcc, Wdr1, Sh3pxd2b, Frmd5	26	4.15	1.85E−06
transmembrane receptor protein serine/threonine kinase signaling pathway	Comp, Lox, Aspn, Adamtsl2, Gdf15, Fstl1, Col1a2, Tgfb2, Scx, Tgfb3, Ltbp4, Fbn1, Col3a1, Sfrp1, Zyx, Rasl11b, Tgfbr1, Wwtr1, Gdf6, Akap2, Sulf1, Bmp10, Hes1, Fstl3, Emilin1, Fzd1, Spry1, Nbl1, Smpd3, Skil, Ltbp3, Jade2, Tbx20, Pakap, Htra3, Hipk2, Adam9, Inhbb, Id1, Atoh8, Inhba, Fbn2, Cav3, Il17rd, Zfp423	45	4.12	1.43E−12
angiogenesis	Tnfrsf12a, Rtn4, Sparc, Hbegf, Ace, Ccn2, Nr4a1, Adamts1, Hspb1, Col4a2, Otulin, Cfh, Tgfb2, S1pr1, Itga5, Col4a1, Serpinf1, Adgrg1, Loxl2, Gpx1, Ptgis, Angptl7, Tgfbr1, Tnfrsf1a, Flt4, Srpx2, Adamts9, Amot, Fbln5, Col4a3, Sulf1, Ptk2b, Ptprb, Emilin1, Plcd3, B4galt1, Cyp1b1, Chil1, Klf4, Amotl1, Hspb6, Sox18, Gna13, Sox17, Itgb3, Sphk1, Ecm1, Fgfr1, Clic4, Adgra2, Tbx20, Hmox1, C3, Hipk2, Id1, Sars, Shc1, Kctd10, Rhoj, Reck, Col18a1, Rgcc, Grn	63	4.03	4.73E−18
positive regulation of smooth muscle cell proliferation	Mmp2, Hbegf, Adamts1, S1pr1, Sulf1, Itgb3, Smpd3, Ccn4, Tgm2, Irak4, Hmox1, Il6ra, Shc1, Igfbp5	14	3.96	0.027177
biomineral tissue development	Comp, Lox, Aspn, Col1a2, Col1a1, Tmem119, S1pr1, Tgfb3, Sbno2, Atf4, Ptk2b, Fam20a, Smpd3, Ltbp3, Ecm1, Sgms2, Fbn2, Ahsg	18	3.95	0.001623
ERBB signaling pathway	Nppa, Rtn4, Hbegf, Hip1, App, Ptk2b, Ptpn11, Ptpn12, Errfi1, Ptprf, Iqgap1, Ncf1, Shc1, Ptpn2	14	3.88	0.033943
cellular response to growth factor stimulus	Ankrd1, Comp, Lox, Sparc, Aspn, Adamtsl2, Gdf15, Fstl1, Col1a2, Ccn2, Nr4a1, Hspb1, Col4a2, Dbn1, Tgfb2, Scx, Tgfb3, Ltbp4, Fbn1, Col3a1, Sfrp1, Zyx, Rasl11b, Sulf2, Myof, Tgfbr1, Flt4, Gdf6, Sulf1, Bmp10, Hes1, Fstl3, Emilin1, Fzd1, Rapgef1, Spry1, Ptpn11, Col2a1, Klf4, Nbl1, Ptpn1, Myo1c, Itgb3, Smpd3, Skil, Sphk1, Ltbp3, Atp7a, Grb10, Fgfr1, Ptpn12, Adgra2, Dmd, Zfp36, Htra3, Hipk2, Adam9, Id1, Dync1li1, Ptprf, Iqgap1, Shc1, Ehd4, Fbn2, Cav3, Ddit4, Fgf6, Micall1, Adamts12, Pde8a, Il17rd, Zfp423	72	3.81	1.55E−19
regulation of extrinsic apoptotic signaling pathway	Tnfrsf12a, Tgfb2, Gpx1, Sfrp1, Tgfbr1, Bcl2l1, Col2a1, Skil, Runx3, Rnf34, Lmna, Fgfr1, Hmox1, Ltbr, Inhba, Atf3, Tnfrsf23, Gas1, Traf2	19	3.8	0.001463
respiratory tube development	Lox, Mgp, Sparc, Adamtsl2, Ace, Ccn2, Tnc, Ctsz, Adamts2, Loxl3, Rcn3, Flt4, Mmp14, Hes1, Spry1, Chil1, Pcsk5, Smpd3, Ltbp3, Atp7a, Fgfr1, Errfi1, Id1, Man2a1, Igfbp5	25	3.79	2.68E−05
response to mechanical stimulus	Ankrd1, Ankrd23, Mmp2, Csrp3, Col1a1, Scx, Pkd2, Tlr4, Ptpn11, Chil1, Piezo2, Serpine2, Nfkbia, Dmd, Cnn2, Pkd1, Cav3	17	3.73	0.007358
negative regulation of DNA-binding transcription factor activity	Nlrc3, Dap, Otulin, Pkd2, Nupr1, Ptgis, Hand2, Anxa4, Prnp, Hes1, Cyp1b1, Cmklr1, Klf4, Ctnnbip1, Hmox1, Nfkbia, Id1, Pkd1, Sik1	19	3.72	0.002004
positive regulation of peptidyl-tyrosine phosphorylation	Thbs4, Crlf1, Lrp8, Hbegf, Ace, Itga5, Tlr4, Tnfrsf1a, App, Gprc5b, Prnp, Ptk2b, Hes1, Nedd9, Dok7, Ptpn11, Ptpn1, Itgb3, Il6ra, Mif, Iqgap1, Ncf1, Ehd4	23	3.69	0.000165
tissue migration	Acta1, Rtn4, Sparc, Hbegf, Nr4a1, Hspb1, Tgfb2, Stc1, Serpinf1, Loxl2, Gpx1, Tgfbr1, Flt4, Srpx2, Adamts9, Plcg2, Amot, Anln, Bmp10, Ptk2b, Cyp1b1, Ptpn11, Klf4, Amotl1, Pkn3, Itgb3, Fgfr1, Adgra2, Hmox1, Adam9, Pkn1, Atoh8, Rhoj, Rgcc, Grn	35	3.63	9.99E−08
tissue remodeling	Thbs4, Mmp2, Timp1, Ctsk, Tgfb2, Tmem119, S1pr1, Tgfb3, Gpnmb, Sfrp1, F2r, Hand2, Flt4, Mmp14, Ptk2b, Ctss, Itgb3, Ltbp3, Atp7a, Tgm2, Bak1, Ahsg, Igfbp5	23	3.61	0.000247
circulatory system process	Nppa, Postn, Comp, Mmp2, Svep1, Nppb, Hbegf, Myh7, Ace, Col1a2, Ccn2, Ednrb, Csrp3, Tgfb2, Stc1, Gnao1, Gpx1, F2r, App, Edn3, Wwtr1, Snta1, Amot, Bmp10, Ptgs1, Popdc2, Kcna5, Scpep1, Smpd3, Slc2a1, Ctnnbip1, Tbx20, Hmox1, Dmd, Mif, Shc1, Trpv4, Gjc1, Cav3, Npr3, Aoc3, Myl1	42	2.51	0.000105

Supplemental Table 9. Continued

Pathway/Function Categories	Genes	Gene Count	Fold Enrichment	Adjusted *p*-value
**GO_Biological Process enriched in genes protected from upregulation by RIP140 deficiency during TAC/MI**				
ossification	Comp, Mmp2, Lox, Aspn, Igfbp3, Col1a2, Ccn2, Col1a1, Tgfb2, Tmem119, Stc1, Scx, S1pr1, Tgfb3, Kremen1, Sfrp1, Sbno2, Hand2, Wwtr1, Mmp14, Atf4, Ptk2b, Fzd1, Ptpn11, Col2a1, Smpd3, Ccn4, Ltbp3, Ecm1, Runx3, Lmna, Fgfr1, Ctnnbip1, Igsf10, Id1, Sgms2, Fbn2, Ahsg, Satb2, Cebpd, Sh3pxd2b, Igfbp5	42	3.57	2.03E-09
regeneration	Mmp2, Rtn4, Ace, Tnc, Apod, Kremen1, Gpx1, Sulf2, Ifrd1, Ptgfrn, Klf4, Hmox1, Igsf10, Cdkn1a, Mif, Ptprf, Grn	17	3.47	0.019703
regulation of cellular response to growth factor stimulus	Lox, Aspn, Adamtsl2, Fstl1, Fbn1, Sfrp1, Rasl11b, Sulf2, Myof, Sulf1, Hes1, Fstl3, Emilin1, Fzd1, Spry1, Nbl1, Ptpn1, Myo1c, Skil, Grb10, Fgfr1, Adgra2, Dmd, Htra3, Hipk2, Ptprf, Fbn2, Cav3, Adamts12, Il17rd, Zfp423	31	3.45	4.74E-06
positive regulation of supramolecular fiber organization	Synpo2l, Ccn2, Map1b, Arpc1b, Rhoc, Tgfbr1, App, Ppm1e, Kirrel, Bmp10, Ptk2b, Sorbs3, Hspa1a, Myo1c, Cdc42ep4, Id1, Trpv4, Cav3, Slain2, Rgcc, Wdr1, Sh3pxd2b	22	3.44	0.001117
negative regulation of vasculature development	Sparc, Adamts1, Col4a2, Tgfb2, Serpinf1, Angptl7, Adamts9, Amot, Fbln5, Col4a3, Sulf1, Emilin1, Klf4, Sars, Shc1, Rgcc	16	3.41	0.045404
mesenchyme development	Acta1, Rtn4, Frzb, Meox1, Ednrb, Col1a1, Tgfb2, Scx, Tgfb3, Loxl2, Sfrp1, Sema6b, Loxl3, Tgfbr1, Hand2, Sema3f, Edn3, Wwtr1, Sema3g, Hes1, Spry1, Adam15, Snai1, Fgfr1, Sema7a, Tbx20, Usf3, Dact3, Rgcc, Il17rd	30	3.4	1.19E-05
cardiocyte differentiation	Sorbs2, Pi16, Lrrc10, Csrp3, Col14a1, Tgfb2, Pdlim5, Hand2, Bmp10, Hes1, Popdc2, Nrap, Sox18, Sox17, Ccn4, Lmna, Myh10, Cav3, Sik1	19	3.36	0.00933
ameboidal-type cell migration	Rtn4, Sparc, Hbegf, Pdlim1, Ednrb, Nr4a1, Hspb1, Timp1, Tgfb2, Stc1, Serpinf1, Loxl2, Gpx1, Sema6b, Tgfbr1, Hand2, Sema3f, Edn3, Flt4, Srpx2, Adamts9, Plcg2, Amot, Anln, Sema3g, Bmp10, Ptk2b, Cyp1b1, Ptpn11, Klf4, Amotl1, Pkn3, Sox17, Itgb3, Fgfr1, Adgra2, Sema7a, Hmox1, Adam9, Iqgap1, Pkn1, Atoh8, Rhoj, Rgcc, Grn	45	3.34	3.16E-09
extrinsic apoptotic signaling pathway	Tnfrsf12a, Lcn2, Tgfb2, Gpx1, Sfrp1, Tgfbr1, Bcl2l1, Col2a1, Krt18, Krt8, Skil, Runx3, Rnf34, Lmna, Fgfr1, Hipk1, Hmox1, Bak1, Ltbr, Inhba, Atf3, Tnfrsf23, Gas1, Traf2	24	3.31	0.000688
positive regulation of cell migration	Thbs4, Postn, Mmp2, Rtn4, Sparc, Hbegf, Adamts1, Hspb1, Fam107a, Col1a1, Tgfb2, S1pr1, Itga5, Gpnmb, Fn1, Sema6b, Rhoc, F2r, Tgfbr1, App, Sema3f, Edn3, Flt4, Srpx2, Plcg2, Amot, Mmp14, Lbp, Sema3g, Ptk2b, Nedd9, Cyp1b1, Cmklr1, Pcsk5, Amotl1, Myo1c, Itgb3, Snai1, Ccn4, Sphk1, Atp7a, Pla2g7, Fgfr1, Adgra2, Sema7a, Hmox1, Mif, Adam9, Actn4, Iqgap1, Trpv4, Atoh8, Rhoj, Pdgfc, Col18a1, Aoc3, Grn, Igfbp5	58	3.28	3.52E-12
regulation of vasculature development	Tnfrsf12a, Sparc, Adamts1, Hspb1, Col4a2, Tgfb2, Itga5, Serpinf1, Ptgis, Angptl7, Tnfrsf1a, Srpx2, Adamts9, Amot, Fbln5, Col4a3, Sulf1, Ptk2b, Emilin1, Cyp1b1, Chil1, Klf4, Hspb6, Itgb3, Sphk1, Ecm1, Adgra2, Hmox1, Il6ra, C3, Hipk2, Id1, Sars, Shc1, Rhoj, Reck, Rgcc, Grn	38	3.28	3.28E-07
negative regulation of cytokine production	Nlrc3, Adcy7, Serpinb1c, Apod, Tgfb2, Tgfb3, Gpnmb, Tspo, Fn1, Tlr4, Ssc5d, Tnfrsf1a, Anxa4, Prnp, Lbp, Cmklr1, Klf4, Homer2, Fgfr1, Hmox1, Zfp36, Errfi1, Inhbb, Sars, Ppm1b, Arg1, Mertk, Rgcc	28	3.25	0.000105
regulation of peptidyl-tyrosine phosphorylation	Thbs4, Crlf1, Lrp8, Hbegf, Ace, Itga5, Tlr4, Sfrp1, Tnfrsf1a, App, Gprc5b, Prnp, Ptk2b, Hes1, Nedd9, Dok7, Ptpn11, Ptpn1, Itgb3, Il6ra, Errfi1, Mif, Iqgap1, Ncf1, Ehd4, Ptpn2, Pdgfc	27	3.25	0.000183
respiratory system development	Lox, Mgp, Sparc, Adamtsl2, Ace, Ccn2, Tnc, Ctsz, Adamts2, Loxl3, Rcn3, Flt4, Mmp14, Hes1, Spry1, Chil1, Smpd3, Ltbp3, Atp7a, Fgfr1, Errfi1, Id1, Man2a1, Igfbp5	24	3.24	0.001057
actin filament organization	Synpo2l, Acta1, Ccn2, Pdlim1, Dpysl3, Fam107a, Enah, Dbn1, Mical2, S1pr1, Arpc1b, Rhoc, Zyx, Mical3, Hip1, Tgfbr1, Ppm1e, Kirrel, Akap2, Ptk2b, Nedd9, Pdlim3, Sorbs3, Ppfia1, Elmo1, Eln, Nrap, Fat1, Myo1c, Mprip, Msrb1, Cald1, Cdc42ep4, Pakap, Myh10, Actn4, Id1, Actn1, Trpv4, Cav3, Eps8, Rgcc, Wdr1, Sh3pxd2b	44	3.2	2.39E-08
positive regulation of angiogenesis	Hspb1, Itga5, Ptgis, Tnfrsf1a, Srpx2, Ptk2b, Cyp1b1, Chil1, Klf4, Hspb6, Itgb3, Sphk1, Ecm1, Hmox1, C3, Hipk2, Rhoj, Grn	18	3.19	0.035403
transmembrane receptor protein tyrosine kinase signaling pathway	Nppa, Svep1, Lox, Rtn4, Igfbp3, Hbegf, Gdf15, Igfbp6, Ccn2, Hspb1, Col4a2, Csrp3, Apod, Col1a1, Dok1, Col4a1, Sulf2, Myof, Hip1, Csrnp1, App, Flt4, Col4a3, Sulf1, Ptk2b, Nedd9, Emilin1, Rapgef1, Sla, Spry1, Ptpn11, Col4a5, Plat, Ptpn1, Myo1c, Itgb3, Smpd3, Grb10, Fgfr1, Ptpn12, Adgra2, Errfi1, Ptprf, Iqgap1, Ncf1, Shc1, Ndrg4, Ptpn2, Pdgfc, Eif4ebp1, Ddit4, Rhoq, Fgf6, Ahsg, Mertk, Adamts12, Igfbp5, Igfbp2	58	3.16	1.74E-11
negative regulation of cellular component movement	Igfbp3, Dpysl3, Timp1, Dbn1, Apod, Stc1, Serpinf1, Adgrg1, Col3a1, Sfrp1, Sema6b, Sema3f, Adamts9, Sulf1, Sema3g, Bmp10, Emilin1, Cyp1b1, Klf4, Adam15, Nbl1, Clic4, Sema7a, Adarb1, Mif, Actn4, Cnn2, Actn1, Ndrg4, Reck, Rgcc, Igfbp5, Frmd5	33	3.09	2.38E-05
renal system development	Crlf1, Ace, Adamts1, Cfh, Tgfb2, Fbn1, Col4a1, Nid1, Pkd2, Sfrp1, Sulf2, Tgfbr1, Anxa4, Wwtr1, Col4a3, Sulf1, Bmp10, Hes1, Spry1, Pcsk5, Bicc1, Col4a4, Sox17, Itgb3, Gcnt1, Fgfr1, Ctnnbip1, Il6ra, Iqgap1, Pkd1, Gcnt4	31	3.07	7.84E-05
peptidyl-tyrosine phosphorylation	Thbs4, Crlf1, Lrp8, Hbegf, Ace, Itga5, Tlr4, Sfrp1, Tnfrsf1a, App, Gprc5b, Flt4, Prnp, Ptk2b, Hes1, Nedd9, Sla, Dok7, Ptpn11, Ptpn1, Itgb3, Fgfr1, Il6ra, Errfi1, Mif, Iqgap1, Ncf1, Ehd4, Ptpn2, Pdgfc	30	3.01	0.000201
positive regulation of cytoskeleton organization	Synpo2l, Ccn2, Map1b, Azin1, Arpc1b, Rhoc, Tgfbr1, Ppm1e, Kirrel, Bmp10, Ptk2b, Sorbs3, Hspa1a, Myo1c, Cdc42ep4, Id1, Trpv4, Cav3, Slain2, Rgcc, Wdr1, Sh3pxd2b	22	2.98	0.012443
urogenital system development	Crlf1, Mmp2, Ace, Adamts1, Tnc, Cfh, Tgfb2, Fbn1, Col4a1, Nid1, Serpinf1, Pkd2, Sfrp1, Sulf2, Tgfbr1, Anxa4, Wwtr1, Col4a3, Sulf1, Bmp10, Hes1, Spry1, Pcsk5, Bicc1, Col4a4, Sox17, Itgb3, Gcnt1, Fgfr1, Ctnnbip1, Il6ra, Iqgap1, Pkd1, Gcnt4	34	2.97	3.98E-05
skeletal system morphogenesis	Comp, Mmp2, Ccn2, Col1a1, Tgfb2, Tmem119, Stc1, Scx, Sfrp1, Tgfbr1, Csrnp1, Mmp14, Sulf1, Col2a1, Smpd3, Ltbp3, Trpv4, Pkd1, Fbn2, Fgf6, Satb2, Gas1, Sh3pxd2b	23	2.95	0.008949
bone development	Comp, Lox, Sparc, Col1a1, Tmem119, Stc1, Scx, Fbn1, Sulf2, Mmp14, Sulf1, Ptpn11, Col2a1, Ttc9, Smpd3, Ccn4, Ltbp3, Trpv4, Pdgfc, Sh3pxd2b	20	2.92	0.043556
regulation of ERK1 and ERK2 cascade	Ccn2, Gpnmb, Cavin3, Fn1, Tlr4, F2r, Hand2, App, Flt4, Ptk2b, Emilin1, Rapgef1, Spry1, Ptpn11, Chil1, Klf4, Ptpn1, Itgb3, Sema7a, C3, Dmd, Errfi1, Mif, Shc1, Trpv4, Ndrg4, Ptpn2, Pdgfc, Atf3, Pde8a	30	2.83	0.000793
negative regulation of cell migration	Igfbp3, Dpysl3, Timp1, Apod, Stc1, Serpinf1, Adgrg1, Col3a1, Sfrp1, Adamts9, Sulf1, Bmp10, Emilin1, Cyp1b1, Klf4, Adam15, Nbl1, Clic4, Adarb1, Mif, Cnn2, Ndrg4, Reck, Rgcc, Igfbp5	25	2.75	0.012098
positive regulation of protein kinase activity	Lrp8, Hbegf, Gdf15, Ace, Igfbp6, Tgfb2, Fbn1, Pkd2, Tlr4, F2r, Tgfbr1, App, Gprc5b, Edn3, Clu, Prnp, Ptk2b, Nedd9, Taok3, Dok7, Ptpn11, Chil1, Ccnd2, Ptpn1, Itgb3, Fgfr1, Il6ra, Cdkn1a, Mif, Adam9, Iqgap1, Ncf1, Shc1, Pkd1, Pdgfc, Rgcc, Traf2	37	2.67	0.000155
regulation of DNA-binding transcription factor activity	Nlrc3, Lrp8, Dap, Otulin, Csrp3, Pkd2, Tlr4, Nupr1, Ptgis, Hand2, App, Anxa4, Clu, Abra, Prnp, Hes1, Fzd1, Ifrd1, Cyp1b1, Clock, Cmklr1, Klf4, Sphk1, Ctnnbip1, Hmox1, Nfkbia, Hipk2, Lrrfip1, Id1, Slco3a1, Pkd1, Sik1, Rgcc, Traf2	34	2.63	0.000729
reactive oxygen species metabolic process	Ccn2, Gpx3, Tspo, Pkd2, Tlr4, Gpx1, Ptgis, App, Sod3, Clu, Fbln5, Ptk2b, Cyp1b1, Klf4, Rab27a, Bnip3, Smpd3, Sh3pxd2a, Atp7a, Cdkn1a, Ncf1, Shc1, Ddit4, Sh3pxd2b	24	2.62	0.041836
regulation of Wnt signaling pathway	Nppa, Frzb, Otulin, Col1a1, Kremen1, Sfrp1, Sulf2, App, Gprc5b, Wwtr1, Sulf1, Fzd1, Rapgef1, Bicc1, Sox17, Ccn4, Grb10, Dkk3, Adgra2, Ctnnbip1, Disc1, Ppm1b, Tnks, Reck, Dact3	25	2.61	0.031353
cellular response to organonitrogen compound	Mmp2, Col5a2, Gdf15, Col1a2, Nr4a1, Csrp3, Col1a1, Col4a1, Col16a1, Pkd2, Gck, Tlr4, Gnao1, Col3a1, Sfrp1, Col6a1, App, Prnp, Atf4, Bcl2l1, Rapgef1, Lpin2, Ptpn11, Anxa5, Ptpn1, Myo1c, Lpin3, Smpd3, Atp7a, Sgk1, Grb10, Inhbb, Ptprf, Shc1, Ptpn2, Pdgfc, Eif4ebp1, Rhoq, Ahsg, Castor1, Igfbp5	41	2.58	7.42E-05
regulation of peptidase activity	Ccn2, Nr4a1, Serpinb1c, Dap, Timp1, Serping1, Serpinf1, Gpx1, Hip1, Rcn3, Antxr1, F2r, App, Col4a3, Prelid1, Acer2, Rps6ka3, Anxa8, Klf4, Perp, Ecm1, Rnf34, Serpine2, Fam162a, Bak1, Picalm, Reck, Ahsg, Tnfrsf23, Grn	30	2.57	0.005773
circulatory system process	Nppa, Postn, Comp, Mmp2, Svep1, Nppb, Hbegf, Myh7, Ace, Col1a2, Ccn2, Ednrb, Csrp3, Tgfb2, Stc1, Gnao1, Gpx1, F2r, App, Edn3, Wwtr1, Snta1, Amot, Bmp10, Ptgs1, Popdc2, Kcna5, Scpep1, Smpd3, Slc2a1, Ctnnbip1, Tbx20, Hmox1, Dmd, Mif, Shc1, Trpv4, Gjc1, Cav3, Npr3, Aoc3, Myl1	42	2.51	0.000105
cell junction organization	Svep1, Xirp2, Sparc, Lrp8, Sorbs2, Ace, Fam107a, Tnc, Dbn1, Apod, Tgfb2, Tgfb3, Itga5, Col4a1, Col16a1, Fn1, Rhoc, Pdlim5, F2r, Tgfbr1, Etv5, App, Sema3f, Srpx2, Pdzrn3, Snta1, Prnp, Mmp14, Ptk2b, Fzd1, Rapgef1, Dok7, Ppfia1, Sparcl1, Col4a5, Perp, Myo1c, Itgb3, Snai1, Tln1, Hipk1, Nlgn2, Colq, C3, Ppfibp1, Disc1, Myh10, Actn4, Cdh11, Ptprf, Iqgap1, Actn1, Trpv4, Picalm, Gjc1, Cldn15, Wdr1	57	2.5	4.55E-07
regulation of metal ion transport	Nppa, Fxyd6, Kif5b, Ace, Tgfb2, Stc1, Fxyd5, Tspo, Pkd2, Gck, Gnao1, F2r, Edn3, Plcg2, Snta1, Prnp, Atf4, Ptk2b, Hes1, Kcna5, Itgb3, Atp7a, Homer2, Sgk1, Serpine2, Dmd, Mif, Fhl1, Bak1, Cav3, Hecw2, Sik1, Nkain4, Plcb4	34	2.5	0.002247
negative regulation of cell population proliferation	Nppb, Nlrc3, Sparc, Igfbp3, Frzb, P3h2, Apod, Fam129b, Tgfb2, Tgfb3, Btg2, Gpnmb, Serpinf1, Tspo, Adgrg1, Sfrp1, Nupr1, F2r, Tgfbr1, App, Prnp, Col4a3, Sulf1, Ptk2b, Hes1, Rapgef1, B4galt1, Comt, Cyp1b1, Spry1, Klf4, Hspa1a, E2f3, Slit3, Prkar1a, Runx3, Lmna, Serpine2, Ctnnbip1, Klf13, Hmox1, Cdkn1a, Adarb1, Ptprf, Bak1, Pkn1, Strn, Atoh8, Inhba, Ndrg4, Ptpn2, Arg1, Npr3, Rgcc, Gas1, Igfbp5	56	2.49	7.32E-07
regulation of actin cytoskeleton organization	Synpo2l, Ankrd23, Ccn2, Fam107a, Dbn1, Tgfb2, S1pr1, Pam, Arpc1b, Rhoc, Tgfbr1, Ppm1e, Kirrel, Bmp10, Ptk2b, Sorbs3, Ppfia1, Eln, Myo1c, Itgb3, Cdc42ep4, Id1, Iqgap1, Cav3, Eps8, Rgcc, Wdr1, Sh3pxd2b	28	2.47	0.026142

Supplemental Table 9. Continued

Pathway/Function Categories	Genes	Gene Count	Fold Enrichment	Adjusted *p*-value
**GO_Biological Process enriched in genes protected from upregulation by RIP140 deficiency during TAC/MI**				
regulation of system process	Nppa, Mmp2, Nppb, Rtn4, Hbegf, Myh7, Pi16, Ace, Ccn2, Ednrb, Dbn1, Csrp3, Col14a1, Tgfb2, Stc1, Tlr4, Gnao1, F2r, Hand2, Tnfrsf1a, App, Edn3, Snta1, Bmp10, Ptk2b, Ctss, Ptgs1, Popdc2, Comt, Kcna5, Ptpn11, Klf4, Lmcd1, Ccn4, Sphk1, Lmna, Fgfr1, Cald1, Rab11fip5, Nlgn2, Dmd, Errfi1, Inhbb, Shc1, Inhba, C1qtnf1, Gjc1, Cav3, Igfbp5	49	2.46	1.51E-05
blood circulation	Nppa, Postn, Comp, Mmp2, Nppb, Hbegf, Myh7, Ace, Col1a2, Ccn2, Ednrb, Csrp3, Tgfb2, Stc1, Gnao1, Gpx1, F2r, App, Edn3, Snta1, Amot, Bmp10, Ptgs1, Popdc2, Kcna5, Scpep1, Smpd3, Ctnnbip1, Tbx20, Hmox1, Dmd, Mif, Shc1, Trpv4, Gjc1, Cav3, Npr3, Aoc3, Myl1	39	2.46	0.000567
regulation of apoptotic signaling pathway	Tnfrsf12a, Hspb1, Tgfb2, Gpx1, Sfrp1, Nupr1, Tgfbr1, Clu, Prelid1, Bcl2l1, Col2a1, Bnip3, Ptpn1, Snai1, Skil, Runx3, Rnf34, Lmna, Fgfr1, Plscr1, Hmox1, Mif, Inhbb, Fam162a, Bak1, Ltbr, Inhba, Ptpn2, Atf3, Tnfrsf23, Gas1, Traf2	32	2.45	0.007532
regulation of neuron projection development	Ankrd1, Tnfrsf12a, Rtn4, Lrp8, Adamts1, Hspb1, Dpysl3, Dbn1, Ctsz, Kremen1, Inpp5j, Map1b, Serpinf1, Fn1, Sfrp1, Sema6b, Pdlim5, Gprc5b, Sema3f, Trak2, Sema3g, Ptk2b, Hes1, Enc1, Fzd1, Dennd5a, Rapgef1, Ifrd1, Klf4, Skil, Sphk1, Sgk1, Fgfr1, Serpine2, Nlgn2, Sema7a, Dmd, Mif, Disc1, Id1, Ptprf, Iqgap1, Trpv4, Picalm, Ndrg4, Hecw2, Grn	47	2.44	4.08E-05
positive regulation of kinase activity	Lrp8, Hbegf, Gdf15, Ace, Igfbp6, Tgfb2, Fbn1, Pkd2, Tlr4, F2r, Tgfbr1, App, Gprc5b, Edn3, Flt4, Clu, Prnp, Ptk2b, Nedd9, Taok3, Dok7, Ptpn11, Chil1, Ccnd2, Ptpn1, Itgb3, Fgfr1, Il6ra, Cdkn1a, Mif, Adam9, Iqgap1, Ncf1, Shc1, Pkd1, Pdgfc, Mertk, Rgcc, Traf2	39	2.44	0.000669
cellular response to nitrogen compound	Mmp2, Col5a2, Sorbs2, Gdf15, Col1a2, Nr4a1, Csrp3, Col1a1, Col4a1, Col16a1, Pkd2, Gck, Tlr4, Gnao1, Col3a1, Sfrp1, Col6a1, App, Prnp, Atf4, Bcl2l1, Rapgef1, Lpin2, Ptpn11, Anxa5, Ptpn1, Myo1c, Lpin3, Smpd3, Atp7a, Sgk1, Grb10, Inhbb, Ptprf, Shc1, Ptpn2, Pdgfc, Eif4ebp1, Rhoq, Ahsg, Castor1, Igfbp5, Traf2, Ly6c1	44	2.43	0.000133
negative regulation of cell differentiation	Postn, Rtn4, Frzb, Col5a2, Pi16, Ednrb, Dpysl3, Csrp3, Ctsz, Tgfb2, Nmrk2, Tmem119, Fbn1, Kremen1, Inpp5j, Tspo, Adgrg1, Col3a1, Sfrp1, Sema6b, Loxl3, Col5a1, Tgfbr1, Hand2, Fgl2, App, Sema3f, Wwtr1, Trak2, Sema3g, Ptk2b, Hes1, Fstl3, Dennd5a, Ifrd1, Spry1, Ptpn11, Itgb3, Snai1, Ccn4, Skil, Il4ra, Ltbp3, Runx3, Fgfr1, Sema7a, Klf13, Nfkbia, Zfp36, Usf3, Actn4, Id1, Trpv4, Ptpn2, Cav3, Ctr9, Adamts12, Dact3, Jdp2, Il17rd, Igfbp5	61	2.42	3.81E-07
regulation of protein kinase activity	Nppa, Lrp8, Hbegf, Gdf15, Ace, Igfbp6, Hspb1, Tgfb2, Fbn1, Pkd2, Tlr4, Uchl1, Sfrp1, F2r, Tgfbr1, App, Gprc5b, Ppm1e, Edn3, Wwtr1, Clu, Prnp, Ptk2b, Nedd9, Ptprb, Taok3, Dok7, Spry1, Ptpn11, Chil1, Ccnd2, Ptpn1, Itgb3, Prkar1a, Fgfr1, Il6ra, Cdkn1a, Adarb1, Errfi1, Mif, Adam9, Iqgap1, Ncf1, Shc1, Pkn1, Pkd1, Ptpn2, Pdgfc, Cav3, Rgcc, Traf2	51	2.4	1.67E-05
visual system development	Col8a1, Mfap5, Col5a2, Cfh, Tgfb2, Col4a1, Col5a1, Angptl7, Tgfbr1, Atf4, Cyp1b1, Spry1, Klf4, Ninj1, Fat1, Skil, Hipk1, Clic4, C3, Hipk2, Inhbb, Myh10, Bak1, Inhba, Rhoj, Man2a1, Mertk, Gas1, Sh3pxd2b	29	2.39	0.033403
leukocyte migration	Thbs4, Ednrb, Apod, Tgfb2, S1pr1, Itga9, App, Edn3, Mmp14, Lbp, Ptk2b, Emilin1, B4galt1, Cmklr1, Nbl1, Itgb3, Smpd3, Gcnt1, Ecm1, Pla2g7, Irak4, Mif, Ch25h, Trpv4, Msn, Aoc3, Eps8, Wdr1	28	2.39	0.047512
regulation of actin filament-based process	Synpo2l, Ankrd23, Ccn2, Fam107a, Dbn1, Csrp3, Tgfb2, Stc1, S1pr1, Pam, Arpc1b, Rhoc, Tgfbr1, Ppm1e, Kirrel, Bmp10, Ptk2b, Sorbs3, Ppfia1, Eln, Myo1c, Itgb3, Cdc42ep4, Id1, Cnn2, Iqgap1, Cav3, Eps8, Rgcc, Wdr1, Sh3pxd2b	31	2.38	0.019331
regulation of cytokine production	Postn, Nlrc3, Adcy7, Serpinb1c, Hspb1, Apod, Tgfb2, Tgfb3, Gpnmb, Tspo, Fn1, Tlr4, Ssc5d, Sulf2, F2r, Tnfrsf1a, App, Gprc5b, Flt4, Anxa4, Plcg2, Clu, Prnp, Lbp, Sulf1, Atf4, Ifngr1, Cyp1b1, Tril, Ptpn11, Cmklr1, Trim16, Klf4, Ccn4, Sphk1, Il4ra, Homer2, Fgfr1, Sema7a, Hmox1, Il6ra, C3, Zfp36, Errfi1, Mif, Inhbb, Sars, Ppm1b, Iqgap1, Trpv4, Arid5a, Arg1, Mertk, Rgcc, Traf2	55	2.37	7.05E-06
response to peptide	Mmp2, Gdf15, Ednrb, Nr4a1, Csrp3, Gck, Tlr4, App, Prnp, Lpin2, Ptpn11, Trim16, Anxa5, Ptpn1, Myo1c, Lpin3, Smpd3, Sgk1, Grb10, Nfkbia, Inhbb, Ptprf, Shc1, Trpv4, Ptpn2, Eif4ebp1, Rhoq, Ahsg, Igfbp5	29	2.36	0.043389
sensory system development	Col8a1, Mfap5, Col5a2, Cfh, Tgfb2, Col4a1, Col5a1, Angptl7, Tgfbr1, Atf4, Cyp1b1, Spry1, Klf4, Ninj1, Fat1, Skil, Hipk1, Clic4, C3, Hipk2, Inhbb, Myh10, Bak1, Inhba, Rhoj, Man2a1, Mertk, Gas1, Sh3pxd2b	29	2.36	0.043389
negative regulation of protein phosphorylation	Nppa, Igfbp3, Hspb1, Dok1, Inpp5j, Tlr4, Uchl1, Sfrp1, Ppm1e, Kirrel, Wwtr1, Prnp, Ptprb, Emilin1, Rapgef1, Taok3, Spry1, Klf4, Ptpn1, Prkar1a, Dmd, Cdkn1a, Ppp1r15a, Adarb1, Errfi1, Bak1, Pkn1, Ptpn2, Cav3, Fam129a, Ddit4, Atf3	32	2.35	0.018534
positive regulation of response to external stimulus	Nppa, Thbs4, Ace, Hspb1, S1pr1, Fn1, Tlr4, Nupr1, Tnfrsf1a, App, Gprc5b, Edn3, Lbp, Ptk2b, Ctss, Clock, Cmklr1, Ccn4, Pla2g7, Tgm2, Napepld, Fgfr1, Plscr1, C3, Nfkbia, Mif, Trpv4, Arg1, Aoc3, Ap1g1, Grn	31	2.32	0.030678
negative regulation of phosphorylation	Nppa, Igfbp3, Hspb1, Dok1, Inpp5j, Tlr4, Uchl1, Sfrp1, Nupr1, Ppm1e, Kirrel, Wwtr1, Prnp, Ptprb, Emilin1, Rapgef1, Taok3, Spry1, Klf4, Ptpn1, Prkar1a, Grb10, Dmd, Cdkn1a, Ppp1r15a, Adarb1, Errfi1, Bak1, Pkn1, Ptpn2, Cav3, Midn, Fam129a, Ddit4, Atf3	35	2.31	0.009651
response to inorganic substance	Mmp2, Fibin, Adcy7, Rasa4, Nr4a1, Lcn2, Pam, Pkd2, Fn1, Loxl2, Gpx1, App, Sod3, Plcg2, Prnp, Slc38a2, Fbln5, Ptk2b, Cyp1b1, Anxa5, Cp, Bnip3, Smpd3, Krt8, Sphk1, Atp7a, Clic4, Hmox1, Adam9, Iqgap1, Ncf1, Pde8a, Syt12, Traf2	34	2.31	0.013053
negative regulation of transport	Igfbp3, Ace, Apod, Tgfb2, Stc1, Map1b, Pkd2, Fn1, Gck, Gnao1, Sfrp1, F2r, Tnfrsf1a, Edn3, Atf4, Ptk2b, Hes1, Ptgs1, Comt, Ptpn11, Anxa5, Itgb3, Atp7a, Grb10, Rab11fip5, Serpine2, Hmox1, Ube2g2, Cttnbp2nl, Inhbb, Cnn2, Picalm, Cav3, Hecw2, Midn, Rhoq, Entpd1, Rgcc, Plcb4	39	2.29	0.003504
negative regulation of immune system process	Nlrc3, Apod, Tgfb2, Tgfb3, Fbn1, Serping1, Gpnmb, Gpx1, Col3a1, Sfrp1, Loxl3, Fgl2, Prnp, Ptk2b, Hes1, Fstl3, Emilin1, Nbl1, Prkar1a, Il4ra, Runx3, Tsc22d3, Klf13, Hmox1, Nfkbia, Zfp36, Mif, Ppm1b, Pkn1, Ptpn2, Arg1, Ctr9, Mertk, Grn	34	2.22	0.03106
regulation of proteolysis	Ccn2, Nr4a1, Serpinb1c, Dap, Timp1, Ctsz, Ltbp4, Serping1, Serpinf1, Gpx1, Nupr1, Hip1, Rcn3, Antxr1, F2r, App, Clu, Mmp14, Col4a3, Prelid1, Ptk2b, Acer2, Rps6ka3, Anxa8, Klf4, Hspa1a, Rnf19b, Plat, Perp, Ecm1, Rnf34, Sh3d19, Serpine2, Ube2g2, Hipk2, Adam9, Disc1, Fam162a, Bak1, Picalm, Pkd1, Spon1, Reck, Ahsg, Tnfrsf23, Fbxw11, Gas1, Grn	48	2.21	0.000557
positive regulation of transferase activity	Lrp8, Hbegf, Gdf15, Ace, Igfbp6, Tgfb2, Fbn1, Pkd2, Tlr4, F2r, Tgfbr1, App, Gprc5b, Edn3, Flt4, Clu, Prnp, Ptk2b, Nedd9, Taok3, Dok7, Ptpn11, Chil1, Ccnd2, Klf4, Ptpn1, Itgb3, Fgfr1, Il6ra, Cdkn1a, Mif, Adam9, Iqgap1, Ncf1, Shc1, Pkd1, Tnks, Pdgfc, Mertk, Rgcc, Traf2	41	2.21	0.004488
regulation of cytoskeleton organization	Synpo2l, Ankrd23, Ccn2, Fam107a, Dbn1, Tgfb2, S1pr1, Pam, Inpp5j, Map1b, Azin1, Arpc1b, Rhoc, Tgfbr1, Ppm1e, Kirrel, Bmp10, Ptk2b, Sorbs3, Ppfia1, Eln, Hspa1a, Myo1c, Itgb3, Sgk1, Clip3, Cdc42ep4, Id1, Dync1li1, Iqgap1, Trpv4, Pkd1, Chmp4c, Cav3, Slain2, Eps8, Rgcc, Wdr1, Sh3pxd2b	39	2.21	0.007862
axon development	Mmp2, Tnfrsf12a, Rtn4, Kif5b, Tnc, Enah, Dbn1, Apod, Tgfb2, Kremen1, Map1b, Fn1, Uchl1, Sema6b, App, Sema3f, Trak2, Sema3g, Ifrd1, Ptpn11, Klf4, Raph1, Slit3, Skil, Runx3, Sema7a, Adarb1, Mif, Disc1, Myh10, Cdh11, Ptprf, Picalm, Gas1, Grn	35	2.2	0.028909
multicellular organismal homeostasis	Dio2, Ccn2, Ednrb, Ctsk, Col14a1, Lcn2, Tmem119, Scx, S1pr1, Tlr4, Gpx1, Rcn3, F2r, Wwtr1, Atf4, Ptk2b, Ctss, Ptgs1, Ptpn11, Col2a1, Cmklr1, Itgb3, Il4ra, Ltbp3, Homer2, Slc2a1, Napepld, Grb10, Id1, Trpv4, Pdgfc, Npr3, Ahsg, Prkab2, Zfp423	35	2.16	0.041426
regulation of defense response	Nppa, Nlrc3, Lrp8, Ace, Adcy7, Ednrb, Otulin, Apod, Serping1, Serpinf1, Tlr4, Gpx1, Nupr1, Ptgis, Sbno2, Tnfrsf1a, Fgl2, Gprc5b, Lbp, Ctss, Clock, Ccn4, Sphk1, Apobec3, Tgm2, Napepld, Fgfr1, Plscr1, Sema7a, C3, Nfkbia, Zfp36, Mif, Ppm1b, Trpv4, Ptpn2, Arg1, Aoc3, Ahsg, Adamts12, Ap1g1, Grn	42	2.13	0.008837
regulation of ion transport	Nppa, Fxyd6, Rtn4, Kif5b, Ace, Tgfb2, Stc1, Fxyd5, Tspo, Pkd2, Gck, Gnao1, F2r, App, Edn3, Plcg2, Snta1, Prnp, Prelid1, Atf4, Ptk2b, Hes1, Ctss, Ifngr1, Kcna5, Itgb3, Atp7a, Homer2, Sgk1, Fgfr1, Serpine2, Nlgn2, Dmd, Mif, Fhl1, Bak1, Arg1, Cav3, Hecw2, Entpd1, Sik1, Nkain4, Wdr1, Syt12, Plcb4	45	2.11	0.004814
positive regulation of cell development	Ankrd1, Tnfrsf12a, Rtn4, Lrp8, Ace, Adamts1, Hspb1, Dpysl3, Dbn1, Map1b, Serpinf1, Tspo, Fn1, Star, Etv5, App, Gprc5b, Gdf6, Bmp10, Ptk2b, Hes1, Nedd9, Enc1, Fzd1, Rapgef1, Nbl1, Itgb3, Ccn4, Skil, Sphk1, Sgk1, Fgfr1, Jade2, Serpine2, Nlgn2, Sema7a, Dmd, Mif, Disc1, Ptprf, Iqgap1, Picalm, Ndrg4, Man2a1, Grn	45	2.11	0.005023
negative regulation of cellular component organization	Nlrc3, Rtn4, Ace, Dpysl3, Fam107a, Dbn1, Apod, Ctsz, S1pr1, Kremen1, Inpp5j, Map1b, Tspo, Gpx1, Nupr1, Sema6b, Antxr1, Tnfrsf1a, Sema3f, Trak2, Clu, Prnp, Mmp14, Prelid1, Sema3g, Hes1, Emilin1, Dennd5a, Hist1h1c, Ifrd1, Ppfia1, Arhgap24, Bnip3, Sgk1, Lmna, Clip3, Ctnnbip1, Sema7a, Tbx20, Id1, Dync1li1, Ptprf, Trpv4, Picalm, Tnks, Eif4ebp1, Cav3, Eps8, Rgcc	49	2.08	0.002582
regulation of transferase activity	Nppa, Lrp8, Hbegf, Gdf15, Ace, Igfbp6, Hspb1, Tgfb2, Fbn1, Pkd2, Tlr4, Uchl1, Sfrp1, F2r, Tgfbr1, App, Gprc5b, Ppm1e, Edn3, Flt4, Wwtr1, Clu, Prnp, Ptk2b, Nedd9, Ptprb, Taok3, Dok7, Spry1, Ptpn11, Chil1, Ccnd2, Klf4, Ptpn1, Itgb3, Prkar1a, Fgfr1, Il6ra, Cdkn1a, Zfp36, Adarb1, Errfi1, Mif, Adam9, Iqgap1, Ncf1, Shc1, Pkn1, Pkd1, Tnks, Ptpn2, Pdgfc, Cav3, Midn, Mertk, Rgcc, Traf2	57	2.05	0.000517
response to abiotic stimulus	Ankrd1, Dio2, Ankrd23, Mmp2, Mfap4, Hspb7, Csrp3, Col1a1, Tgfb2, Scx, Tgfb3, Pam, Pkd2, Tlr4, Loxl2, Gpx1, Col3a1, Sfrp1, Myof, Ptgis, App, Sod3, Ctss, Bcl2l1, Clock, Ptpn11, Chil1, Ccnd2, Piezo2, Hspa1a, Plat, Fosl2, Bnip3, Lrrc8c, Krt8, Rnf34, Tsc22d3, Rdh11, Sgk1, Lmna, Rab11fip5, Serpine2, Hmox1, Nfkbia, Dmd, Cdkn1a, Fam162a, Cnn2, Bak1, Pkn1, Trpv4, Pkd1, Ndrg4, Eif4ebp1, Lrrc8a, Cav3, Ddit4, Sik1, Aldh18a1, Rgcc, Fndc1	61	2.04	0.000238
positive regulation of organelle organization	Synpo2l, Kif5b, Ccn2, Hspa1l, Map1b, Azin1, Arpc1b, Rhoc, Tgfbr1, App, Ppm1e, Edn3, Kirrel, Ube2c, Bmp10, Ptk2b, Resf1, Sorbs3, Hspa1a, Bnip3, Myo1c, Smpd3, Sphk1, Lmna, Cdc42ep4, Fam162a, Id1, Bak1, Trpv4, Msn, Tnks, Arid5a, Cav3, Slain2, Ctr9, Jdp2, Rgcc, Grn, Wdr1, Sh3pxd2b	40	2.04	0.040139
regulation of apoptotic process	Ankrd1, Comp, Crlf1, Mmp2, Tnfrsf12a, Lox, Igfbp3, Lrp8, Frzb, Ace, Ccn2, Ednrb, Nr4a1, Hspb1, Dap, Timp1, Phlda3, Fam129b, Ctsz, Tgfb2, Scx, Btg2, Itga5, Tspo, Fn1, Tlr4, Gpx1, Sfrp1, Nupr1, Hip1, Star, Ptgis, F2r, Tgfbr1, Hand2, Tnfrsf1a, App, Flt4, Clu, Prnp, Col4a3, Prelid1, Atf4, Ptk2b, Bcl2l1, Acer2, B4galt1, Grk5, Rps6ka3, Cyp1b1, Col2a1, Ccnd2, Klf4, Anxa5, Hspb6, Krt18, Bnip3, E2f3, Ptpn1, Snai1, Skil, Sphk1, Atp7a, Runx3, Rnf34, Tsc22d3, Tgm2, Sgk1, Lmna, Fgfr1, Plscr1, Clip3, Hmox1, Cdkn1a, Zfp36, Mif, Hipk2, Inhbb, Fam162a, Actn4, Id1, Ptprf, Bak1, Shc1, Ltbr, Inhba, Ptpn2, Dnajc5, Atf3, Col18a1, Tnfrsf23, Mertk, Rgcc, Gas1, Grn, Fndc1, Traf2	97	2.01	5.06E-08
regulation of programmed cell death	Ankrd1, Comp, Crlf1, Mmp2, Tnfrsf12a, Lox, Igfbp3, Lrp8, Frzb, Ace, Ccn2, Ednrb, Nr4a1, Hspb1, Dap, Timp1, Phlda3, Fam129b, Ctsz, Tgfb2, Scx, Btg2, Itga5, Tspo, Fn1, Tlr4, Gpx1, Sfrp1, Nupr1, Hip1, Star, Ptgis, F2r, Tgfbr1, Hand2, Tnfrsf1a, App, Flt4, Plcg2, Clu, Prnp, Col4a3, Prelid1, Atf4, Ptk2b, Bcl2l1, Acer2, B4galt1, Grk5, Rps6ka3, Cyp1b1, Col2a1, Ccnd2, Klf4, Anxa5, Hspb6, Krt18, Bnip3, E2f3, Ptpn1, Snai1, Skil, Sphk1, Atp7a, Runx3, Rnf34, Tsc22d3, Tgm2, Sgk1, Lmna, Fgfr1, Plscr1, Clip3, Hmox1, Cdkn1a, Zfp36, Mif, Hipk2, Inhbb, Fam162a, Actn4, Id1, Ptprf, Bak1, Shc1, Ltbr, Inhba, Ptpn2, Dnajc5, Atf3, Col18a1, Tnfrsf23, Mertk, Rgcc, Gas1, Grn, Fndc1, Traf2	98	2	5.46E-08

The table shows the significantly enriched (p<0.05, adjusted p-value) GOTERM_BP_DIRECT and Kyoto Encyclopedia of Genes and Genomes (KEGG)_PATHWAY pathways defined by the gene sets protected from downregulation by RIP140 deficiency during TAC/MI

Supplemental Table 10. The percentage of genes in pathways protected from downregulation/upregulation by RIP140 deletion during TAC/MI.

KEGG_Pathway	**% of genes protected from downregulation by RIP140 deletion during TAC/MI**
Fatty acid elongation	63.63636
Cardiac muscle contraction	50
Valine, leucine and isoleucine degradation	46.875
Retrograde endocannabinoid signaling	45.94595
Oxidative phosphorylation	44.77612
2-Oxocarboxylic acid metabolism	44.44444
Thermogenesis	41.97531
Fructose and mannose metabolism	40
Metabolic pathways	37.35849
Fatty acid degradation	36.36364
Propanoate metabolism	34.78261
Mismatch repair	33.33333
Biosynthesis of amino acids	29.41176
Pyruvate metabolism	29.41176
Fatty acid metabolism	28.57143
Butanoate metabolism	27.27273
Tryptophan metabolism	26.66667
Peroxisome	25.92593
Carbon metabolism	23.07692
Citrate cycle (TCA cycle)	17.64706
Glyoxylate and dicarboxylate metabolism	9.090909

GO_Biological Process	**% of genes protected from downregulation by RIP140 deletion during TAC/MI**
electron transport coupled proton transport	75
glutathione metabolic process	66.66667
regulation of striated muscle contraction	61.90476
heart contraction	59.18367
heart process	59.18367
regulation of heart rate	57.14286
coenzyme A metabolic process	57.14286
cellular modified amino acid metabolic process	56
branched-chain amino acid metabolic process	50
nucleoside triphosphate metabolic process	47.61905
organophosphate catabolic process	47.61905
ribonucleoside triphosphate biosynthetic process	47.05882
fatty acid beta-oxidation using acyl-CoA dehydrogenase	44.44444
ATP biosynthetic process	43.75
monovalent inorganic cation transport	43.63636
actin-mediated cell contraction	42.85714
lipid catabolic process	41.66667
fatty acid beta-oxidation	41.37931
cellular lipid catabolic process	41.30435
proton transmembrane transport	40.90909
sulfur compound metabolic process	40.27778
2-oxoglutarate metabolic process	40
monocarboxylic acid catabolic process	39.47368
monocarboxylic acid metabolic process	37.5
ubiquinone biosynthetic process	37.5
oxidative phosphorylation	37.2093
carbohydrate derivative metabolic process	37.06897
cellular amino acid catabolic process	36.84211
ATP synthesis coupled proton transport	36.36364
acyl-CoA metabolic process	36.11111
thioester metabolic process	36.11111
oxoacid metabolic process	33.54839
mitochondrial respiratory chain complex assembly	33.33333
mitochondrion organization	33.33333
cellular ketone metabolic process	33.33333
carboxylic acid metabolic process	33.33333
succinate metabolic process	33.33333
generation of precursor metabolites and energy	30.85106
ATP synthesis coupled electron transport	30.76923
dicarboxylic acid metabolic process	30.76923
aerobic respiration	30.76923
mitochondrial transport	30.23256
NADH dehydrogenase complex assembly	29.41176
mitochondrial respiratory chain complex I assembly	29.41176
nucleoside phosphate biosynthetic process	28.88889
cellular respiration	28.57143
mitochondrial electron transport, NADH to ubiquinone	28.57143
oxidation-reduction process	28.44828
cellular amino acid metabolic process	28.26087
acetyl-CoA metabolic process	20
tricarboxylic acid cycle	18.18182
short-chain fatty acid metabolic process	14.28571
acyl-CoA biosynthetic process	0
thioester biosynthetic process	0

Supplemental Table 10. Continued

KEGG_Pathway	**% of genes protected from upregulation by RIP140 deletion during TAC/MI**
ECM-receptor interaction	69.69697
Protein digestion and absorption	66.66667
PI3K-Akt signaling pathway	60.56338
Dilated cardiomyopathy (DCM)	54.54545
Hypertrophic cardiomyopathy (HCM)	54.16667
Complement and coagulation cascades	54.16667
HIF-1 signaling pathway	53.84615
Focal adhesion	52.94118
Relaxin signaling pathway	51.42857
Osteoclast differentiation	46.66667
TGF-beta signaling pathway	45.83333
Platelet activation	43.33333
Regulation of actin cytoskeleton	42.10526
Phospholipase D signaling pathway	41.93548
Apoptosis	41.37931
Hippo signaling pathway	41.17647
Phagosome	40.90909
Estrogen signaling pathway	39.28571
MAPK signaling pathway	38.98305
Rap1 signaling pathway	38.09524
EGFR tyrosine kinase inhibitor resistance	36.84211
Hippo signaling pathway - multiple species	33.33333
Axon guidance	31.57895
Bacterial invasion of epithelial cells	26.08696
Fc gamma R-mediated phagocytosis	23.33333

GO_Biological Process	**% of genes protected from upregulation by RIP140 deletion during TAC/MI**
collagen fibril organization	84
collagen-activated tyrosine kinase receptor signaling pathway	75
platelet-derived growth factor receptor signaling pathway	73.68421
cellular response to acid chemical	72.22222
negative regulation of cell growth	70.27027
positive regulation of protein tyrosine kinase activity	69.23077
tumor necrosis factor-mediated signaling pathway	68.75
response to muscle stretch	66.66667
regulation of protein tyrosine kinase activity	66.66667
vascular endothelial growth factor production	66.66667
regulation of extrinsic apoptotic signaling pathway	65.51724
negative regulation of cellular response to growth factor stimulus	64.51613
negative regulation of cartilage development	63.63636
trabecula formation	63.63636
regulation of vascular endothelial growth factor production	63.63636
collagen biosynthetic process	61.90476
regulation of cell growth	61.64384
respiratory tube development	60.97561
regeneration	60.71429
BMP signaling pathway	60.60606
striated muscle cell development	60
cartilage development	59.64912
epidermal growth factor receptor signaling pathway	59.09091
regulation of actomyosin structure organization	59.09091
integrin-mediated signaling pathway	58.33333
cell growth	57.77778
regulation of collagen biosynthetic process	56.25
positive regulation of cell-matrix adhesion	56.25
smooth muscle cell proliferation	56.09756
ERBB signaling pathway	56
regulation of transmembrane receptor protein serine/threonine kinase signaling pathway	56
respiratory system development	55.81395
connective tissue development	55.55556
SMAD protein signal transduction	55
peptidyl-tyrosine dephosphorylation	54.54545
response to tumor necrosis factor	54.28571
cellular response to growth factor stimulus	54.19847
regulation of smooth muscle cell migration	54.16667
enzyme linked receptor protein signaling pathway	54.14365
cell-matrix adhesion	54
positive regulation of nitric oxide biosynthetic process	53.84615
striated muscle cell differentiation	53.62319
negative regulation of blood vessel morphogenesis	53.57143
positive regulation of peptidyl-tyrosine phosphorylation	53.48837
positive regulation of stress-activated MAPK cascade	53.33333
regulation of Wnt signaling pathway	53.19149
positive regulation of inflammatory response	52.94118
positive regulation of actin filament bundle assembly	52.94118
cell adhesion mediated by integrin	52.63158
negative regulation of ERK1 and ERK2 cascade	52.63158
regulation of collagen metabolic process	52.63158

Supplemental Table 10. Continued

GO_Biological Process	% of genes protected from upregulation by RIP140 deletion during TAC/MI
regulation of cytokine production involved in immune response	52.38095
regulation of actin filament bundle assembly	52.17391
response to transforming growth factor beta	52.08333
muscle cell differentiation	51.89873
mesenchyme development	51.78571
negative regulation of DNA-binding transcription factor activity	51.42857
positive regulation of cell-substrate adhesion	51.35135
circulatory system development	51.08225
cellular response to transforming growth factor beta stimulus	51.06383
regulation of inflammatory response	50.76923
positive regulation of cell population proliferation	50
cell-cell junction organization	50
positive regulation of smooth muscle cell migration	50
negative regulation of developmental process	49.15254
negative regulation of cell population proliferation	49.12281
positive regulation of apoptotic process	48.62385
reactive oxygen species biosynthetic process	48
cellular component assembly involved in morphogenesis	48
regulation of DNA-binding transcription factor activity	47.14286
regulation of anatomical structure morphogenesis	47
negative regulation of cellular component movement	46.47887
activation of protein kinase activity	44.18605
regulation of response to external stimulus	44.13793
regulation of protein kinase activity	43.75
cell morphogenesis involved in differentiation	43.75
regulation of ossification	43.75
positive regulation of response to external stimulus	43.05556
regulation of cell adhesion mediated by integrin	42.85714
actin filament-based process	42.77457
movement of cell or subcellular component	42.73504
negative regulation of response to external stimulus	42.62295
negative regulation of cell motility	42.62295
platelet activation	42.30769
cell-substrate junction organization	42.30769
regulation of kinase activity	42.27642
cell morphogenesis	41.43646
positive regulation of neurogenesis	41.41414
regulation of cytokine production	41.22137
positive regulation of developmental process	41.2
actin filament organization	40.74074
positive regulation of organelle organization	40.40404
heart morphogenesis	40.35088
cytosolic calcium ion transport	39.39394
branching morphogenesis of an epithelial tube	39.39394
negative regulation of supramolecular fiber organization	39.28571
positive regulation of sprouting angiogenesis	38.46154
regulation of protein localization to membrane	38.23529
sprouting angiogenesis	38.23529
positive regulation of leukocyte chemotaxis	38.09524
axonogenesis	38.02817
cardiac chamber development	37.83784
regulation of interleukin-1 beta production	37.5
cardiac septum development	37.5
regulation of protein-containing complex assembly	37.17949
regulation of cellular component size	36.92308
positive regulation of endocytosis	36.66667
regulation of actin cytoskeleton organization	36.58537
regulation of protein kinase B signaling	36.58537
positive regulation of protein secretion	36.17021
positive regulation of cell adhesion	36.14458
regulation of protein localization to plasma membrane	36
ventricular septum morphogenesis	35.71429
positive regulation of blood vessel endothelial cell migration	35
positive regulation of protein-containing complex assembly	34.88372
small GTPase mediated signal transduction	34.72222
interleukin-1 beta production	33.33333
protein depolymerization	33.33333
cardiac chamber morphogenesis	33.33333
regulation of blood vessel endothelial cell migration	32.14286
regulation of hemostasis	31.57895
pri-miRNA transcription by RNA polymerase II	31.25
vascular endothelial growth factor signaling pathway	30.76923
regulation of interleukin-1 production	30
positive regulation of osteoblast differentiation	30
regulation of blood coagulation	27.77778
positive regulation of protein kinase B signaling	27.58621
homotypic cell-cell adhesion	26.31579
cell migration involved in sprouting angiogenesis	26.31579
negative regulation of coagulation	23.07692
regulation of actin filament length	22.5
cell junction disassembly	22.22222
atrioventricular valve morphogenesis	18.18182
synapse pruning	16.66667

Supplemental Table 11. Pathways enriched in overlapped genes between upregulated genes in cs*Nrip1*$^{-/-}$ from RNA-seq and genes with H3K27ac up in cs*Nrip1*$^{-/-}$ from CUT&RUN-seq.

Pathway/Function Categories	Genes	Gene Count	Fold Enrichment	Adjusted *p*-value
**GO_Biological Process enriched in overlapped genes**				
glutathione metabolic process	Gpx3, Mgst2, Glo1, Gclm, Mgst1, Sod1, Gsta1	7	10.33	0.009824
negative regulation of MAPK cascade	Itgb1bp1, Cnksr3, Sh3rf2, Dmd, Prkn, Dynlt1b, Inpp5k, Dusp8, Tlr4, Mecom, Dusp4	11	5.67	0.008375
cellular modified amino acid metabolic process	Gpx3, Cpt1a, Mgst2, Glo1, Gclm, Shmt1, Mgst1, Slc27a1, Sod1, Gsta1	10	5.59	0.025995
regulation of cellular carbohydrate metabolic process	Acacb, Prkn, Hsd11b1, Inpp5k, Obp2a, Ppp1r3g, P2ry1, Nln, Gnb3, Prkg1	10	5.38	0.036356
lipid modification	Fabp3, Echdc2, Eci2, Cpt1a, Mtmr11, Acacb, Inpp5f, St3gal2, Inpp5k, Obp2a, Adipor2, Agtr1a	12	5.32	0.005928
regulation of carbohydrate metabolic process	Acacb, Gpd1, Prkn, Hsd11b1, Inpp5k, Obp2a, Ppp1r3g, P2ry1, Nln, Gnb3, Prkg1	11	4.95	0.030262
cellular carbohydrate metabolic process	Acacb, Phkg1, Prkn, Hsd11b1, Inpp5k, Hk1, Obp2a, Ppp1r3g, P2ry1, Nln, Ip6k1, Gnb3, Prkg1	13	4.13	0.03557
carbohydrate metabolic process	Pgm5, Cpt1a, Acacb, Phkg1, St3gal2, Glo1, Gpd1, Prkn, Hsd11b1, Inpp5k, Hk1, Obp2a, Ppp1r3g, P2ry1, Nln, Ip6k1, Gnb3, Prkg1	18	3.58	0.006563
lipid biosynthetic process	Tecrl, Fabp3, Dctn6, Ptgds, Acacb, Ccn1, Acsm5, St3gal2, Pnpla1, Hsd11b1, Obp2a, Cyp27a1, Prxl2b, Slc27a1, Gpam, Sgms1, Ip6k1, Sod1, Ugcg, Prkg1	20	3.09	0.016621
organophosphate metabolic process	Gphn, Fabp3, Mtmr11, Acacb, Ctps, Inpp5f, Pfkfb3, Nampt, Plbd2, Acsm5, Gpd1, Pank1, Prkn, Shmt1, Hsd11b1, Inpp5k, Hk1, P2ry1, Suclg2, Slc27a1, Gpam, Nt5e, Sgms1, Ip6k1, Gnb3, Prkg1	26	2.93	0.001841
oxoacid metabolic process	Syk, Tecrl, Fabp3, Echdc2, Eci2, Cpt1a, Ptgds, Acacb, Mgst2, Acsm5, Glo1, Gpd1, Prune2, Gclm, Shmt1, Hk1, Obp2a, Cyp27a1, Prxl2b, Suclg2, Tlr4, Slc27a1, Gpam, Pcbd1, Adipor2, Asrgl1, Gsta1	27	2.87	0.001762
regulation of cellular catabolic process	Fez2, Ehmt2, Hfe, Cpt1a, Cttn, Herc1, Pttg1ip, Acacb, Pan3, Nampt, Bmf, Gpd1, Sh3rf2, Prkn, Hsd11b1, Bnip3l, Foxo3, Obp2a, Tent5b, Ambra1, Tnrc6a, Klhl22, Tent5a	23	2.83	0.014695
carboxylic acid metabolic process	Syk, Tecrl, Fabp3, Echdc2, Eci2, Cpt1a, Ptgds, Acacb, Mgst2, Acsm5, Glo1, Gpd1, Gclm, Shmt1, Hk1, Obp2a, Cyp27a1, Prxl2b, Suclg2, Tlr4, Slc27a1, Gpam, Pcbd1, Adipor2, Asrgl1, Gsta1	26	2.8	0.00434
organic acid metabolic process	Syk, Tecrl, Fabp3, Echdc2, Eci2, Cpt1a, Ptgds, Acacb, Mgst2, Acsm5, Glo1, Gpd1, Prune2, Gclm, Shmt1, Hk1, Obp2a, Cyp27a1, Prxl2b, Suclg2, Tlr4, Slc27a1, Gpam, Pcbd1, Adipor2, Asrgl1, Gsta1	27	2.76	0.003615
cellular lipid metabolic process	Tecrl, Fabp3, Echdc2, Eci2, Cpt1a, Mtmr11, Ptgds, Acacb, Inpp5f, Plbd2, Ccn1, Mgst2, Acsm5, St3gal2, Gpd1, Pnpla1, Inpp5k, Obp2a, Prxl2b, Slc27a1, Gpam, Adipor2, Sgms1, Ip6k1, Gnb3, Gsta1, Ugcg, Agtr1a	28	2.73	0.002736
regulation of catabolic process	Fez2, Ehmt2, Hfe, Nedd4l, Cpt1a, Cttn, Herc1, Pttg1ip, Acacb, Pan3, Nampt, Arel1, Bmf, Gpd1, Sh3rf2, Prkn, Hsd11b1, Bnip3l, Foxo3, Obp2a, Tent5b, Ambra1, Fbxl20, Tnrc6a, Klhl22, Tent5a	26	2.69	0.009072
lipid metabolic process	Tecrl, Fabp3, Dctn6, Echdc2, Eci2, Cpt1a, Mtmr11, Ptgds, Acacb, Inpp5f, Plbd2, Ccn1, Mgst2, Acsm5, St3gal2, Gpd1, Pnpla1, Hsd11b1, Inpp5k, Obp2a, Cyp27a1, Prxl2b, Ambra1, Slc27a1, Gpam, Adipor2, Sgms1, Ip6k1, Gnb3, Sod1, Gsta1, Ugcg, Prkg1, Agtr1a	34	2.51	0.001296
small molecule metabolic process	Syk, Tecrl, Fabp3, Echdc2, Eci2, Cpt1a, Ptgds, Coq8a, Acacb, Ctps, Nampt, Mgst2, Acsm5, Glo1, Gpd1, Prune2, Pank1, Gclm, Prkn, Shmt1, Inpp5k, Hk1, Obp2a, Cyp27a1, Ppp1r3g, P2ry1, Prxl2b, Nln, Suclg2, Tlr4, Slc27a1, Car4, Gpam, Nt5e, Pcbd1, Adipor2, Ip6k1, Gnb3, Sod1, Asrgl1, Gsta1, Prkg1	42	2.44	0.000111
phosphorus metabolic process	Syk, Gphn, Fabp3, Hfe, Ick, Sh3bp5, Itgb1bp1, Mtmr11, Coq8a, Acacb, Dtnbp1, Pdgfd, Cnksr3, Phkg1, Hbegf, Ctps, Inpp5f, Pfkfb3, Nampt, Plbd2, Ccn1, Acsm5, Gpd1, Sh3rf2, Vldlr, Prune2, Pank1, Dmd, Prkn, Shmt1, Pdp1, Hsd11b1, Sbk1, Inpp5k, Clk3, Dusp8, Hk1, P2ry1, Eya3, Ambra1, Suclg2, Tlr4, Tns2, Slc27a1, Gpam, Nt5e, Phka2, Adipor2, Sgms1, Sema4d, Ip6k1, Dusp4, Gnb3, Sod1, Grm1, Nek7, Lrp8, Prkg1, Agtr1a, Usp15	60	2.18	5.34E-06
phosphate-containing compound metabolic process	Syk, Gphn, Fabp3, Hfe, Ick, Sh3bp5, Itgb1bp1, Mtmr11, Coq8a, Acacb, Dtnbp1, Pdgfd, Cnksr3, Phkg1, Hbegf, Ctps, Inpp5f, Pfkfb3, Nampt, Plbd2, Ccn1, Acsm5, Gpd1, Sh3rf2, Vldlr, Pank1, Dmd, Prkn, Shmt1, Pdp1, Hsd11b1, Sbk1, Inpp5k, Clk3, Dusp8, Hk1, P2ry1, Eya3, Ambra1, Suclg2, Tlr4, Tns2, Slc27a1, Gpam, Nt5e, Phka2, Adipor2, Sgms1, Sema4d, Ip6k1, Dusp4, Gnb3, Sod1, Grm1, Nek7, Lrp8, Prkg1, Agtr1a, Usp15	59	2.16	1.06E-05
homeostatic process	Syk, Unc13b, Fabp3, Hfe, Nedd4l, Gcnt2, Acacb, Tprkb, P2rx5, Gramd1b, Slc46a2, Dmd, Gclm, Prkn, Pnpla1, Slc9a3, Inpp5k, Hk1, Foxo3, Obp2a, Ppp1r3g, P2ry1, Slc24a2, Tlr4, Tns2, Slc27a1, Car4, Gpam, Mecom, Sp1, Adipor2, Tesc, Ip6k1, Fth1, Gnb3, Sod1, Grm1, Nek7, Btbd9, Ugcg, Rif1, Prkg1, Agtr1a	43	2.03	0.010597
cellular catabolic process	Fez2, Gpx3, Ehmt2, Echdc2, Hfe, Eci2, Nedd4l, Cpt1a, Cttn, Herc1, Pttg1ip, Acacb, Map1lc3a, Pan3, Inpp5f, Nampt, Plbd2, Arel1, Mgst2, Bmf, Zranb1, Glo1, Man1a, Gpd1, Sh3rf2, Prune2, Prkn, Shmt1, Pnpla1, Fbxo31, Hsd11b1, Bnip3l, Foxo3, Obp2a, Tent5b, Ambra1, Nt5e, Fbxl20, Siah1a, Tnrc6a, Asrgl1, Klhl22, Tent5a	43	2.03	0.010746

Pathway/Function Categories	Genes	Gene Count	Fold Enrichment	Adjusted *p*-value
**KEGG_Pathway enriched in overlapped genes**				
Mitophagy - animal	Prkn, Bnip3l, Foxo3, Ambra1, Sp1, Usp15	6	6.83	0.021227

The table shows the significantly enriched (p<0.05, adjusted p-value) GOTERM_BP_DIRECT and Kyoto Encyclopedia of Genes and Genomes (KEGG)_PATHWAY pathways defined by the overlapped gene sets between upregulated genes in cs*Nrip1*$^{-/-}$ from RNA-seq and genes with H3K27ac up in cs*Nrip1*$^{-/-}$ from CUT&RUN-seq.

Supplemental Table 12. Pathways enriched in overlapped genes between protected downregulation genes by RIP140 deletion post TAC/MI from RNA-seq and genes with H3K27ac up in cs*Nrip1*$^{-/-}$ from CUT&RUN-seq.

Pathway/Function Categories	Genes	Gene Count	Fold Enrichment	Adjusted *p*-value
**GO_Biological Process enriched in overlapped genes**				
adult heart development	Myh7, Adra1a, Myh10, Tcap	4	23.59	0.038145
regulation of cardiac muscle contraction	Smtn, Scn10a, Kcnj2, Strit1, Trpm4, Adrb1, Dmd, Adra1a	8	9.71	0.003241
regulation of heart rate	Scn10a, Kcnj2, Myh7, Trpm4, Scn4b, Popdc2, Adrb1, Dmd, Adra1a, Crhr2	10	8.6	0.000507
negative regulation of cellular response to growth factor stimulus	Nbl1, Apln, Ptprf, Htra1, Hipk2, Hjv, Crim1, Htra3, Sulf2	9	7.82	0.004562
acyl-CoA metabolic process	Acsm5, Ces1d, Mcee, Acot7, Sucla2, Gpam, Dgat2, Suclg2	8	7.77	0.017688
positive regulation of stress-activated MAPK cascade	Tnfrsf19, Sphk1, Tpd52l1, Tlr4, Gadd45a, Hipk2, Crhr2, Traf2, Dixdc1	9	6.76	0.01541
positive regulation of ion transport	Map2k6, Arc, Kcnj2, Camk2a, P2ry1, Kcnip2, Strit1, Trdn, Agtr1a, Scn4b, Tesc, Adrb1, Dmd, Crhr2, Dtnbp1	15	4.2	0.006651
actin filament-based process	Asap3, Enah, Smtn, Nedd9, Kcnj2, Mical2, Parvb, Myh7, Strit1, Trpm4, Scn4b, Adrb1, Frmd5, Lmod3, Myh10, Amot, Tcap, Mical3, Mprip, Cavin3, Crhr2, Rhoq, Ppfia1, Dixdc1, Ezr, Dtnbp1	26	2.95	0.00167
positive regulation of protein phosphorylation	Sphk1, Map2k6, Nedd9, Tpd52l1, Tlr4, Ptpn11, Tab1, Gadd45a, Ccnd2, P2ry1, Slco3a1, Adam9, Hipk2, Epha4, Thbs4, Slc27a1, Ghr, Agtr1a, Lrp8, Adra1a, Grm1, Il15, Mprip, Traf2, Hbegf, Dtnbp1	26	2.83	0.003712
regulation of protein kinase activity	Map2k6, Nedd9, Tpd52l1, Hspb1, Tlr4, Ptpn11, Tab1, Prkar1b, Gadd45a, Ccnd2, Adam9, Epha4, Slc27a1, Ghr, Agtr1a, Lrp8, Adra1a, Grm1, Traf2, Hbegf, Dtnbp1	21	2.79	0.047563
neuron projection development	Enah, Sphk1, Nbl1, Arc, Tnc, Sema3f, Cecr2, Ptprf, Hspb1, Ptpn11, Camk2a, Bcl11b, Slit3, Lrtm1, Whrn, Epha4, Thbs4, Cdh23, Ankrd1, Lrp8, Dmd, Fkbp4, Foxo6, Gorasp1, Myh10, Clic5, Kremen1, Tprn, Inpp5j, Adamts1, Disc1, Dixdc1, Dtnbp1	33	2.7	0.000403
positive regulation of phosphorylation	Sphk1, Map2k6, Nedd9, Tpd52l1, Apln, Tlr4, Ptpn11, Tab1, Gadd45a, Ccnd2, P2ry1, Slco3a1, Adam9, Hipk2, Epha4, Thbs4, Slc27a1, Ghr, Agtr1a, Lrp8, Adra1a, Grm1, Il15, Mprip, Traf2, Hbegf, Dtnbp1	27	2.69	0.006131
cellular response to endogenous stimulus	Insig1, Nbl1, Apln, Ptprf, Tlr4, Ptpn11, Htra1, Thrb, Camk2a, Tab1, P2ry1, Scx, Adam9, Trpm4, Hipk2, Epha4, Slc27a1, Ghr, Ankrd1, Agtr1a, Gpam, Fkbp4, Grb14, Hjv, Crim1, Htra3, Stat5a, Sulf2, Rhoq, Col4a2, Ezr, Dtnbp1	32	2.49	0.00358
positive regulation of phosphate metabolic process	Sphk1, Map2k6, Nedd9, Tpd52l1, Apln, Tlr4, Ptpn11, Tab1, Gadd45a, Ccnd2, P2ry1, Slco3a1, Adam9, Hipk2, Epha4, Thbs4, Slc27a1, Ghr, Agtr1a, Lrp8, Adra1a, Grm1, Il15, Mprip, Traf2, Hbegf, Dtnbp1	27	2.47	0.027311
regulation of anatomical structure morphogenesis	Sparc, Sphk1, Tenm4, Nedd9, Arc, Sema3f, Ptprf, Hspb1, Gadd45a, Parvb, P2ry1, Scx, Hipk2, Epha4, Agtr1a, Lrp8, Gorasp1, Myh10, Amot, Adamts1, Crhr2, Disc1, Rhoq, Dixdc1, Col4a2, Ezr, Dtnbp1	27	2.47	0.027855
cation transport	Kcnd2, Scn10a, Arc, Kcnj3, Kcnj2, Homer2, Camk2a, Plcb4, Kcnip2, Abcc9, Strit1, Trpm4, Trdn, Dhrs7c, Cdh23, Agtr1a, Scn4b, Tesc, Adrb1, Syt12, Dmd, Fkbp4, Slc25a42, Slc22a3, Entpd1, Adra1a, Slc22a4, Crhr2, Dtnbp1	29	2.42	0.019275
ion transport	Kcnd2, Map2k6, Scn10a, Ces1d, Arc, Kcnj3, Kcnj2, Homer2, Camk2a, Plcb4, P2ry1, Slco3a1, Kcnip2, Abcc9, Strit1, Trpm4, Trdn, Dhrs7c, Cdh23, Slc27a1, Agtr1a, Scn4b, Tesc, Adrb1, Syt12, Dmd, Fkbp4, Slc25a42, Slc22a3, Entpd1, Adra1a, Slc22a4, Grm1, Slc16a7, Clic5, Crhr2, Dtnbp1	37	2.37	0.001682
regulation of protein phosphorylation	Sphk1, Map2k6, Nedd9, Tpd52l1, Hspb1, Tlr4, Ptpn11, Tab1, Prkar1b, Gadd45a, Ccnd2, P2ry1, Slco3a1, Adam9, Hipk2, Epha4, Thbs4, Slc27a1, Ghr, Agtr1a, Lrp8, Dmd, Adra1a, Grm1, Il15, Mprip, Inpp5j, Traf2, Hbegf, Dtnbp1	30	2.26	0.048164
neurogenesis	Atxn1, Tppp, Enah, Ppp2r3a, Sphk1, Nbl1, Tenm4, Arc, Tnc, Sema3f, Cecr2, Ptprf, Hspb1, Tlr4, Ptpn11, Thrb, Camk2a, Bcl11b, Mdga1, Slit3, Lrtm1, P2ry1, Kcnip2, Hipk2, Whrn, Epha4, Chac1, Thbs4, Cdh23, Ankrd1, Lrp8, Dmd, Fkbp4, Foxo6, Gorasp1, Myh10, Clic5, Kremen1, Tprn, Inpp5j, Adamts1, Disc1, Dixdc1, Dtnbp1	44	2.17	0.001391
positive regulation of molecular function	Asap3, Sphk1, Map2k6, Nedd9, Tpd52l1, Arc, Tlr4, Ptpn11, Camk2a, Tab1, Gadd45a, Perp, Ccnd2, Slco3a1, Strit1, Adam9, Trdn, Hipk2, Epha4, Slc27a1, Ghr, Agtr1a, Tesc, Dap, Lrp8, Adrb1, Dmd, Adra1a, Tnks, Fam129b, Grm1, Pdp1, Crhr2, Traf2, Rcn3, Hbegf	36	2.16	0.018449
plasma membrane bounded cell projection organization	Enah, Sphk1, Nbl1, Arc, Tnc, Sema3f, Cecr2, Ptprf, Hspb1, Ptpn11, Camk2a, Bcl11b, Slit3, Lrtm1, Parvb, Whrn, Epha4, Ift81, Thbs4, Cdh23, Ankrd1, Lrp8, Dmd, Fkbp4, Foxo6, Gorasp1, Myh10, Clic5, Kremen1, Tprn, Inpp5j, Adamts1, Disc1, Rhoq, Dixdc1, Ezr, Dtnbp1, Ttc30a1	38	2.15	0.011676
generation of neurons	Atxn1, Enah, Ppp2r3a, Sphk1, Nbl1, Tenm4, Arc, Tnc, Sema3f, Cecr2, Ptprf, Hspb1, Ptpn11, Thrb, Camk2a, Bcl11b, Mdga1, Slit3, Lrtm1, Kcnip2, Hipk2, Whrn, Epha4, Thbs4, Cdh23, Ankrd1, Lrp8, Dmd, Fkbp4, Foxo6, Gorasp1, Myh10, Clic5, Kremen1, Tprn, Inpp5j, Adamts1, Disc1, Dixdc1, Dtnbp1	40	2.13	0.008064
positive regulation of protein metabolic process	Rnf19b, Ppp2r3a, Sphk1, Map2k6, Nedd9, Tpd52l1, Tlr4, Ptpn11, Tab1, Gadd45a, Samd4, Jdp2, Perp, Ccnd2, P2ry1, Slco3a1, Adam9, Hipk2, Epha4, Thbs4, Slc27a1, Ghr, Arel1, Agtr1a, Dap, Lrp8, Adra1a, Grm1, Il15, Mprip, Disc1, Traf2, Rcn3, Hbegf, Ezr, Dtnbp1	36	2.13	0.025694
positive regulation of cellular protein metabolic process	Rnf19b, Sphk1, Map2k6, Nedd9, Tpd52l1, Tlr4, Ptpn11, Tab1, Gadd45a, Samd4, Jdp2, Perp, Ccnd2, P2ry1, Slco3a1, Adam9, Hipk2, Epha4, Thbs4, Slc27a1, Ghr, Agtr1a, Dap, Lrp8, Adra1a, Grm1, Il15, Mprip, Disc1, Traf2, Rcn3, Hbegf, Ezr, Dtnbp1	34	2.13	0.047283
phosphate-containing compound metabolic process	Atxn1, Acsm5, Ppp2r3a, Sphk1, Map2k6, Aak1, Nedd9, Ces1d, Tpd52l1, Apln, Phkg1, Ptprf, Hspb1, Mast4, Gpd2, Tlr4, Ptpn11, Pla2g4e, Camk2a, Tab1, Adcy7, Prkar1b, Gadd45a, Mcee, Ccnd2, P2ry1, Slco3a1, Adam9, Hipk2, Epha4, Thbs4, Slc27a1, Ghr, Agtr1a, Acot7, Coq8a, Sucla2, Lrp8, Fitm1, Gpam, Dmd, Cish, Entpd1, Adra1a, Tnks, Grm1, Pdp1, Il15, Pank1, Nampt, Stat5a, Mprip, Inpp5j, Pitpnc1, Traf2, Rhoq, Dgat2, Hbegf, Suclg2, Dtnbp1	60	2.12	1.69E-05
cell projection organization	Enah, Sphk1, Nbl1, Arc, Tnc, Sema3f, Cecr2, Ptprf, Hspb1, Ptpn11, Camk2a, Bcl11b, Slit3, Lrtm1, Parvb, Whrn, Epha4, Ift81, Thbs4, Cdh23, Ankrd1, Lrp8, Dmd, Fkbp4, Foxo6, Gorasp1, Myh10, Clic5, Kremen1, Tprn, Inpp5j, Adamts1, Disc1, Rhoq, Dixdc1, Ezr, Dtnbp1, Ttc30a1	38	2.12	0.016218
protein phosphorylation	Sphk1, Map2k6, Aak1, Nedd9, Tpd52l1, Phkg1, Hspb1, Mast4, Tlr4, Ptpn11, Camk2a, Tab1, Prkar1b, Gadd45a, Ccnd2, P2ry1, Slco3a1, Adam9, Hipk2, Epha4, Thbs4, Slc27a1, Ghr, Agtr1a, Lrp8, Dmd, Adra1a, Tnks, Grm1, Il15, Stat5a, Mprip, Inpp5j, Traf2, Hbegf, Dtnbp1	36	2.12	0.029993
anatomical structure morphogenesis	Tppp, Sparc, Insig1, Enah, Ppp2r3a, Sphk1, Nbl1, Tenm4, Nedd9, Arc, Tnc, Sema3f, Cecr2, Ptprf, Hspb1, Egflam, Ptpn11, Thrb, Mical2, Camk2a, Pcsk5, Tab1, Bcl11b, Prkar1b, Gadd45a, Slit3, Lrtm1, Parvb, Perp, P2ry1, Col8a1, Myh7, Scx, Adam9, Hipk2, Whrn, Epha4, Crispld2, Sh3pxd2a, Thbs4, Sbno2, Cdh23, Adamts15, Ankrd1, Agtr1a, Lrp8, Dmd, Lmod3, Gorasp1, Myh10, Amot, Tcap, Clic5, Stat5a, Sulf2, Tprn, Adamts1, Crhr2, Disc1, Rhoq, Dixdc1, Col4a2, Hbegf, Ezr, Dtnbp1	65	2.1	4.61E-06
phosphorus metabolic process	Atxn1, Acsm5, Ppp2r3a, Sphk1, Map2k6, Aak1, Nedd9, Ces1d, Tpd52l1, Apln, Phkg1, Ptprf, Hspb1, Mast4, Gpd2, Tlr4, Ptpn11, Pla2g4e, Camk2a, Tab1, Adcy7, Prkar1b, Gadd45a, Mcee, Ccnd2, P2ry1, Slco3a1, Adam9, Hipk2, Epha4, Thbs4, Slc27a1, Ghr, Agtr1a, Acot7, Coq8a, Sucla2, Lrp8, Fitm1, Gpam, Dmd, Cish, Entpd1, Adra1a, Tnks, Grm1, Pdp1, Il15, Pank1, Nampt, Stat5a, Mprip, Inpp5j, Pitpnc1, Traf2, Rhoq, Dgat2, Hbegf, Suclg2, Dtnbp1	60	2.1	2.28E-05
phosphorylation	Atxn1, Sphk1, Map2k6, Aak1, Nedd9, Tpd52l1, Apln, Phkg1, Hspb1, Mast4, Tlr4, Ptpn11, Camk2a, Tab1, Prkar1b, Gadd45a, Ccnd2, P2ry1, Slco3a1, Adam9, Hipk2, Epha4, Thbs4, Slc27a1, Ghr, Agtr1a, Coq8a, Lrp8, Dmd, Adra1a, Tnks, Grm1, Il15, Stat5a, Mprip, Inpp5j, Traf2, Hbegf, Dtnbp1	39	2.04	0.029648

Pathway/Function Categories	Genes	Gene Count	Fold Enrichment	Adjusted *p*-value
**KEGG_Pathway enriched in overlapped genes**				
Adrenergic signaling in cardiomyocytes	Ppp2r3a, Camk2a, Adcy7, Plcb4, Myh7, Agtr1a, Scn4b, Adrb1, Adra1a	9	4.52	0.01486

The table shows the significantly enriched (p<0.05, adjusted p-value) GOTERM_BP_DIRECT and Kyoto Encyclopedia of Genes and Genomes (KEGG)_PATHWAY pathways defined by the overlapped gene sets between protected downregulation genes by RIP140 deletion post TAC/MI from RNA-seq and genes with H3K27ac up in cs*Nrip1*$^{-/-}$ from CUT&RUN-seq.

Supplemental Table 13. Physiological parameters and echocardiographic analysis post TAC or sham procedure in control vs cs*Nrip1*$^{-/-}$ male mice.

	Sham		TAC	
	Control	cs*Nrip1*$^{-/-}$	Control	cs*Nrip1*$^{-/-}$
Heart rate (bpm)	457.8 ± 28.3	434.0 ± 59.8	496.4 ± 39.5	468.3 ± 47.4
LVPWd (mm)	0.673 ± 0.048	0.656 ± 0.106	1.032 ± 0.104 *	1.072 ± 0.158 #
IVSd (mm)	0.865 ± 0.131	0.847 ± 0.116	1.314 ± 0.193 *	1.283 ± 0.216 #
LVIDd (mm)	3.783 ± 0.402	3.896 ± 0.383	4.880 ± 0.800 *	4.128 ± 0.253 †
LVPWs (mm)	1.005 ± 0.104	0.968 ± 0.125	1.179 ± 0.142	1.262 ± 0.240 #
IVSs (mm)	1.466 ± 0.201	1.470 ± 0.127	1.539 ± 0.222	1.516 ± 0.266
LVIDs (mm)	2.314 ± 0.338	2.400 ± 0.440	4.431 ± 0.860 *	3.484 ± 0.315 #†
LV Mass corrected (mg weight)	85.65 ± 18.9	83.83 ± 17.3	224.55 ± 56.9 *	174.43 ± 39.0 #†
EDV (μL)	65.9 ± 13.3	66.8 ± 14.8	117.1 ± 41.3 *	76.2 ± 11.0 †
ESV (μL)	19.2 ± 6.9	21.3 ± 8.1	93.9 ± 39.3 *	51.2 ± 12.0 #†
EF (%)	70.1 ± 4.7	69.0 ± 7.6	22.7 ± 8.6 *	33.1 ± 7.0 #†

Echocardiography was performed on male mice 8 weeks post TAC or sham surgery (n=9-13 per each group). Values are mean ± SEM. * $p < 0.05$ vs control-sham, # $p < 0.05$ vs cs*Nrip1*$^{-/-}$-sham, † $p < 0.05$ vs control-TAC using 2-way ANOVA with Turkey's multiple comparison test. TAC, transverse aortic constriction; LVPWd, left ventricular posterior wall thickness at end-diastole; IVSd, interventricular septum thickness at end-diastole; LVIDd, left ventricular internal dimension at end-diastole; LVPWs, left ventricular posterior wall thickness at end-systole; IVSs, interventricular septum thickness at end-systole; LVIDs, left ventricular internal dimension at end-systole; EDV, end-diastolic volume; ESV, end-systolic volume; EF, ejection fraction

Supplemental Table 14. Primer sequence

For mouse	Forward	Reverse
Acadm	ATGACGGAGCAGCCAATGAT	TAATGGCCGCCACATCAGAG
Acadvl	ATCTCTGCCCAGCGACTTT	TTCTGGCTTGTCCAGAACTG
Bcat2	CGGAACGAGCCTCTACGTG	GCTTGTGTGACCATACCAACA
Bckdha	CTCCTGTTGGGACGATCTGG	CATTGGGCTGGATGAACTCAA
Cd36	TGGAGTGGTGATGTTTGTTGC	AGCCAGTGTATATGTAGGCTCA
Col3a1	TCCCCTGGAATCTGTGAATC	TGAGTCGAATTGGGGAGAAT
Cox2	TGAAGACGTCCTCCACTCATG	CCCTGGTCGGTTTGATGTTA
Cox4i1	TACTTCGGTGTGCCTTCGA	TGACATGGGCCACATCAG
Cox6a2	GCCCTCTGCTCCCTTAACTG	GGGGATTGTGGAAAAGCGTG
Cox7a1	CTGCTGAGGACGCAAAATGA	TTCTCTGCCACACGGTTTTCT
Cpt1b	ATGTCTACCTCCGAAGCAGGA	GCTGCTTGCACATTTGTGTTC
Dgat1	GCGACGGCTACTGGGATCTG	TGCATTACTCAGGATCAGCATCA
Dgat2	GCATTTGACTGGAACACGCC	CTGGTGGTCAGCAGGTTGTG
Ech1	GCTACCGCGATGACAGTTTC	TCAGAGATCGAAGGCTGATGTT
Fabp3	AAGTGGAACGGGCAGGAGA	GAGGAGCGGGCGGTCAG
Gk	CCGTTACTCCACATGGAAGAAAGCTGTG	GCATCTTGGAAATCCGTGAGGTGG
Hadha	TGCATTTGCCGCAGCTTTAC	GTTGGCCCAGATTTCGTTCA
Hadhb	GCCAACAGACTGAGGAAGGA	ACACTGGCAAGGCTGGATT
Idh2	GGAGAAGCCGGTAGTGGAGAT	GGTCTGGTCACGGTTTGGAA
Ivd	GGACGGCGAGTTTCCAGTT	CTCCTCGTTTAGCCCGTTGA
Ldhb	AGTCTCCCGTGCATCCTCAA	AGGGTGTCCGCACTCTTCCT
Lipe	CCAGCCTGAGGGCTTACTG	CTCCATTGACTGTGACATCTCG
Myh6	GGTCCACATTCTTCAGGATTCTCT	CCTTCTCTGACTTTCGGAGGTACT
Ndufv1	CACTGGTGCAGGCTCAGAC	GGCTTTCACAATGTCTGTCG
Nppa	AGTGCGGTGTCCAACACAGA	GACCTCATCTTCTACCGGCATCT
Nppb	GCTGCTTTGGGCACAAGATAG	GCAGCCAGGAGGTCTTCCTA
Nrip1	GAGGCCCGGAGAATCTGAAG	TTTCGTTGCTCACCAAACGC
Pdk4	CCGCTGTCCATGAAGCA	GCAGAAAAGCAAAGGACGTT
Plin1	GGGACCTGTGAGTGCTTCC	GTATTGAAGAGCCGGGATCTTTT
Plin2	GACCTTGTGTCCTCCGCTTAT	CAACCGCAATTTGTGGCTC
Plin3	ATGTCTAGCAATGGTACAGATGC	CGTGGAACTGATAAGAGGCAGG
Plin4	GTGTCCACCAACTCACAGATG	GCACCATTCCTTTTGCAGCAT
Plin5	GGATCACTTCCTGCCCATGAC	ATCCTCCACCGAACCCACTTC
Pnpla2	CAACGCCACTCACATCTACGG	TGAAGGAGGGATGCAGAGGAC
Rplp0	TGGAAGTCCAACTACTTCCTCAA	ATCTGCTGCATCTGCTTGGAG
Scd1	GCAAGGTAATGTGGCTTTGGCTGA	TTAGCACTTGCCCATGTCTCTGGT
Sdha	GAGCTGCATTTGGCCTTT	CAGCATTGATACCTCCCTGT
Slc27a1	AAGGTTCTTGCATCCTATGCTC	TGGATCTTGAAGGTGCCTGT
Tgfb2	CTTCGACGTGACAGACGCT	GCAGGGGCAGTGTAAACTTATT
Tnni3	TCTGCCAACTACCGAGCCTAT	CTCTTCTGCCTCTCGTTCCAT
For human		
NRIP1	GGATCAGGTACTGCCGTTGAC	CTGGACCATTACTTTGACAGGTG
36B4	TCTACAACCCTGAAGTGCTTGAT	GATAGAATGGGGTACTGATGCAA
